# Small RNA-Based Antiviral Defense in the Phytopathogenic Fungus *Colletotrichum higginsianum*

**DOI:** 10.1371/journal.ppat.1005640

**Published:** 2016-06-02

**Authors:** Sonia Campo, Kerrigan B. Gilbert, James C. Carrington

**Affiliations:** 1 Donald Danforth Plant Science Center, St. Louis, Missouri, United States of America; 2 Center for Research in Agricultural Genomics, CSIC-IRTA-UAB-UB, Campus UAB, Bellaterra (Cerdanyola del Vallès), Barcelona, Spain; Chinese Academy of Sciences, CHINA

## Abstract

Even though the fungal kingdom contains more than 3 million species, little is known about the biological roles of RNA silencing in fungi. The *Colletotrichum* genus comprises fungal species that are pathogenic for a wide range of crop species worldwide. To investigate the role of RNA silencing in the ascomycete fungus *Colletotrichum higginsianum*, knock-out mutants affecting genes for three RNA-dependent RNA polymerase (RDR), two Dicer-like (DCL), and two Argonaute (AGO) proteins were generated by targeted gene replacement. No effects were observed on vegetative growth for any mutant strain when grown on complex or minimal media. However, Δ*dcl1*, Δ*dcl1*Δ*dcl2* double mutant, and Δ*ago1* strains showed severe defects in conidiation and conidia morphology. Total RNA transcripts and small RNA populations were analyzed in parental and mutant strains. The greatest effects on both RNA populations was observed in the Δ*dcl1*, Δ*dcl1*Δ*dcl2*, and Δ*ago1* strains, in which a previously uncharacterized dsRNA mycovirus [termed *Colletotrichum higginsianum non-segmented dsRNA virus 1* (ChNRV1)] was derepressed. Phylogenetic analyses clearly showed a close relationship between ChNRV1 and members of the segmented *Partitiviridae* family, despite the non-segmented nature of the genome. Immunoprecipitation of small RNAs associated with AGO1 showed abundant loading of 5’U-containing viral siRNA. *C*. *higginsianum* parental and Δ*dcl1* mutant strains cured of ChNRV1 revealed that the conidiation and spore morphology defects were primarily caused by ChNRV1. Based on these results, RNA silencing involving ChDCL1 and ChAGO1 in *C*. *higginsianum* is proposed to function as an antiviral mechanism.

## Author Summary

*Colletotrichum* sp. comprises a diverse group of fungal pathogens that attack over 3000 plant species worldwide. Understanding the underlying mechanisms that govern fungal development and pathogenicity may enable more effective and sustainable approaches to crop disease management and control. In most organisms, RNA silencing is an important mechanism to control endogenous and exogenous RNA. RNA silencing utilizes small regulatory molecules (small RNAs) produced by proteins called Dicer (DCL), and exercise their function though effector proteins named Argonaute (AGO). Here, we investigated the role of RNA silencing machinery in the fungus *Colletotrichum higginsianum*, by generating deletions in genes encoding RNA silencing components. Severe defects were observed in both conidiation and conidia morphology in the Δ*dcl1*, Δ*dcl1*Δ*dcl2*, and Δ*ago1* strains. Analysis of transcripts and small RNAs revealed an uncharacterized dsRNA virus persistently infecting *C*. *higginsianum*. The virus was shown (1) to be de-repressed in the Δ*dcl1*, Δ*dcl1*Δ*dcl2* and Δ*ago1* strains, and (2) to cause the conidiation and spore mutant phenotypes. Our results indicate that *C*. *higginsianum* employs RNA silencing as an antiviral mechanism to suppress viruses and their debilitating effects.

## Introduction

RNA-mediated silencing mechanisms regulate gene expression at the transcriptional and post-transcriptional level [1]. Although pathways have proliferated and specialized in various lineages, a core RNA silencing mechanism is conserved among plants, animals, fungi and other eukaryotes [2]. Canonical RNA silencing involves highly base-paired or double-stranded RNA (dsRNA) that is processed into 21–30-nucleotide small RNAs by the activity of one or more ribonucleaseIII-like enzymes called Dicer or Dicer-like (DCL) [3]. Small RNAs are incorporated into an RNA-induced silencing complex (RISC) that contains a member of the Argonaute (AGO) protein family [4]. The small RNA programs the complex to recognize target RNA(s) through base pair complementarity, while the AGO protein functions as an effector to modulate the abundance or activity of the target [5,6].

The fungal kingdom comprises an enormous, diverse group of organisms. Two major fungal RNA silencing pathways have been described: the quelling and the meiotic silencing by unpaired DNA (MSUD) pathways [7,8]. Both appear to be effective genome defense mechanisms that operate during asexual (quelling) [9,10] and sexual development (MSUD) [11]. Fungal RNA silencing pathways function in genome protection and have been proposed to be involved in pathogenicity [12], development [13] and antiviral defense [14].

Fungal viruses, or mycoviruses, are widespread. Most characterized mycoviruses have dsRNA genomes packaged in spherical particles (*Totiviridae*, *Partititiviridae*, *Chrysoviridae*) or a (+)single stranded (ssRNA) genome without the ability to form particles (*Hypoviridae*, *Endornaviridae*) [15]. Both (-)ssRNA [16] and ssDNA mycoviruses [17] have also been described. Mycoviruses do not have an extracellular phase, but rather are transmitted vertically by spores or horizontally by hyphal anastomosis [15]. Although mycoviral infections are generally associated with cryptic (non-symptomatic) and latent (expressed under specific conditions) infections, some mycoviruses cause debilitating phenotypes in their host, making mycoviruses a potential tool for the control of fungal plant pathogens [18–20]. In other cases, mycoviruses have coevolved mutualistically with their hosts [21,22]. *Cryphonectria parasitica* is a model filamentous fungus for the study of virus-host interactions [23,24], and provided the first example that RNA silencing functions as an antiviral defence mechanism in fungi [14]. *C*. *parasitica* has four RNA-dependent RNA Polymerases, two Dicer, and four Argonaute genes, but only *dcl-2* and *agl-2* have been shown to have roles in antiviral defense [14,25,26]. Genetic studies have revealed that *C*. *parasitica* antiviral defense is active against members from the *Hypoviridae*, *Reoviridae* [14,25], *Partitiviridae* [27], *Totiviridae* [28] and *Megabirnaviridae* [29] families. Indirect evidence of an RNA silencing-mycovirus interaction has also been described in fungi with killer viruses. In *Saccharomyces cerevisiae* the M satellites of the dsRNA mycovirus L-A from the *Totiviridae* family produce a toxin that kills uninfected neighbour cells but renders the host immune to the toxin [30]. Strains with active RNA silencing suppress the virus and lose the advantage provided by the virus [31]. The incompatibility between the killer virus and the RNA silencing machinery might explain the existence of several RNA silencing-deficient fungi [31,32].

The genus *Colletotrichum* is considered one of the most economically important groups of plant pathogens, causing anthracnose disease in over 3,200 species of monocot and dicot plants [33], with some infections leading to post-harvest losses of up to 100%. *Colletotrichum higginsianum* infects plants of the *Brassicaceae* family, including *Arabidopsis thaliana*, and is emerging as a model for the study of plant-pathogen interactions in dicotyledonous species [34]. *C*. *higginsianum* is a hemibiotrophic fungus that forms an intracellular hyphae during the initial symptomless biotrophic stage before entering a destructive necrotrophic colonization phase. *C*. *higginsianum* has a relatively small haploid genome that was recently sequenced, the ability to be cultured axenically, and stable transformation methods that allow for the analysis of gene function by targeted disruption [35].

The primary goal of this study was to identify and analyze the role of the RNA silencing machinery in the fungal pathogen *C*. *higginsianum*. Knock-out mutants and high-throughput sequencing was used to functionally characterize transcriptomes and small RNA populations in *C*. *higginsianum* mycelia. ChAGO1 and ChDCL1 were determined to be critical for maintaining low levels of accumulation of a novel dsRNA virus, designated as ChNRV1. Production of viral small RNAs was ChDCL1-dependent and loading into ChAGO1 demonstrated a strong preference for 21-nt, 5’U sequences.

## Results and Discussion

### *Colletotrichum higginsianum* RNA silencing machinery

**Fig 1:**
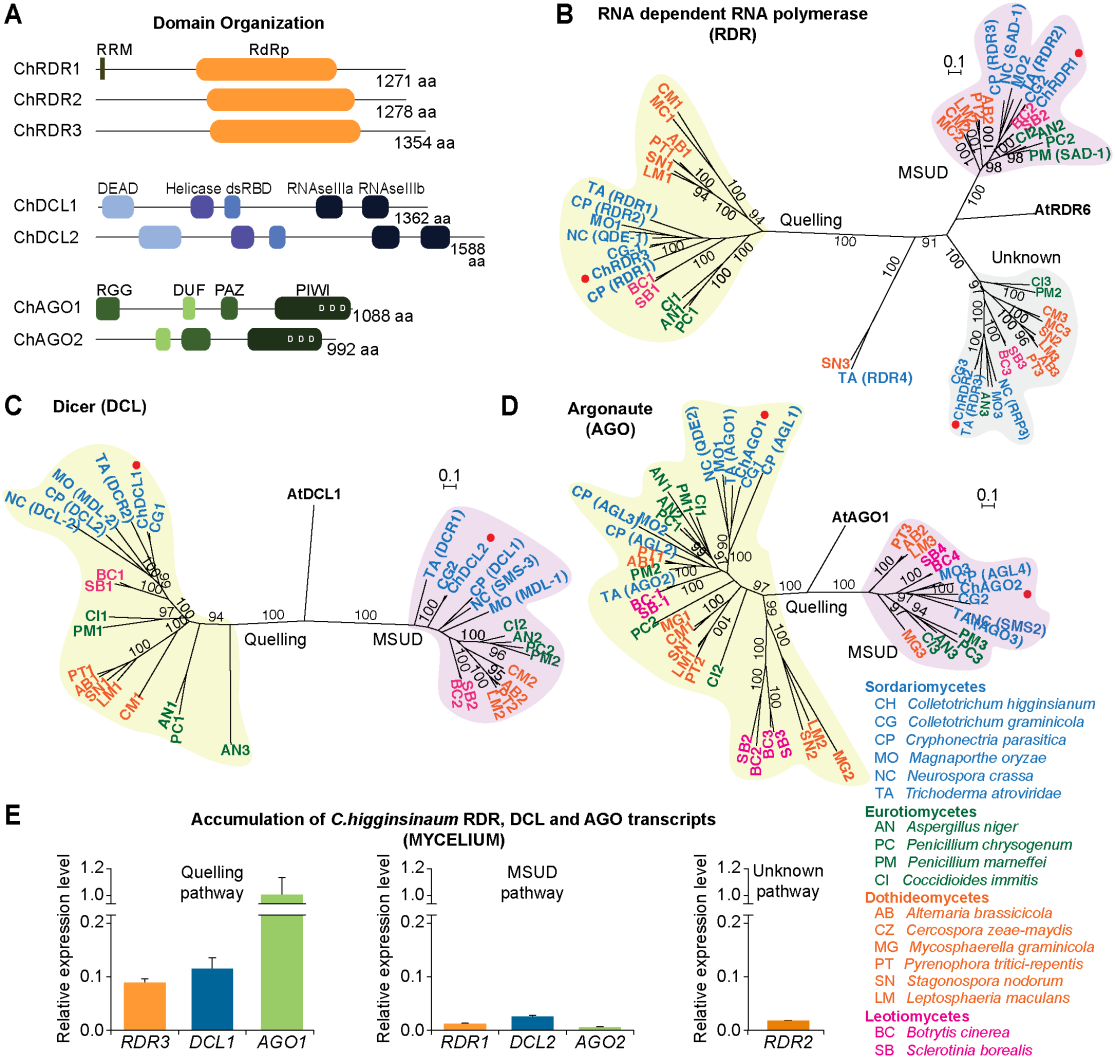
RNA silencing in the ascomycete fungus *Colletotrichum higginsianum*. (**A**) Domain organization of RNA Dependent RNA polymerases (RDR), Dicer (DCL) and Argonaute (AGO) proteins in *C*. *higginsianum*. RRM, RNA Recognition motif. dsRBD, dsRNA Binding Domain. The conserved aspartic acid residues required for AGO catalytic activity in the PIWI domain are indicated (DDD). RGG, arginine-glycine-glycine rich domain. (**B-D**) Phylogenetic analysis of RDR (**B**), DCL (**C**) and AGO (**D**) protein sequences. Rooted maximum likelihood neighbor joining trees were constructed by alignment of full-length protein sequences from representative members of the Ascomycota clade (Sordariomycetes in blue, Eurotiomycetes in green, Dothideomycetes in orange, Leotiomycetes in pink). *C*. *higginsianum* proteins are indicated with red dots. For the sake of clarity, only maximum likelihood bootstraps values higher than 90% are shown. Two main groups are labeled, the Quelling pathway (shaded in green) and the Meiotic-Silencing by Unpaired DNA (MSUD) pathway (shaded in purple). Accession numbers for protein sequences used in the alignment are in S1 Table. Phylogenetic trees were generated using RAxML under the model LG+G+F of amino acid substitution. Scale bar in each panel represents 0.1 amino acid substitutions per site. (**E**) Expression analysis of *RDR*, *DCL* and *AGO* genes in *C*. *higginsianum* mycelium. Silencing genes are grouped into the Quelling pathway (left panel), MSUD pathway (middle panel) and Unknown pathway (right panel). Three biological replicates were used for each gene; values were normalized to the mean of *ACTIN* and *TUBULIN* genes and the mean expression of each RNA silencing gene is represented as a relative value compared to *AGO1*.

Genes encoding three homologs of RNA-dependent RNA Polymerase (RDR1, RDR2 and RDR3), two homologs of Dicer (DCL1 and DCL2) and two homologs of Argonaute (AGO1 and AGO2) were identified in the *Colletotrichum higginsianum* genome (Fig 1A). All three RDRs contained an RNA-dependent RNA Polymerase domain (RdRP). Both DCLs contain an RNA helicase domain, double-stranded RNA-binding domain (dsRBD) and two RNaseIII catalytic domains. Neither DCL contained a PAZ (Piwi/Argonaute/Zwille) domain, which binds the two-nucleotide, 3’ overhangs on canonical DCL substrates [36,37]. Dicer-like proteins in some eukaryotes lack the PAZ domain [38–40] but retain the capacity to generate small RNAs, suggesting the possibility of other substrate recognition mechanisms (S1A Fig). Both *C*. *higginsianum* AGO proteins possess conserved PAZ and PIWI domains, and a conserved MID domain required for 5’ anchoring of the guide RNA. The catalytic triad residues (Aspartic-Aspartic-Aspartic), required for slicer activity [41], are present in the PIWI domain of both proteins (S1B Fig). Additionally, AGO1 contains an amino-terminal RGG box, with 12 copies of the Arginine-Glycine-Glycine motif (Fig 1A). The RGG domain functions in nucleic-acid binding and protein-protein interactions, and is present in some other eukaryotic AGO proteins [39,42,43].

The evolutionary relationships of *C*. *higginsianum* RDRs, DCLs and AGOs with those of other fungal species from the Ascomycota clade was assessed by phylogenetic analysis of protein sequences for each family. Members of *C*. *higginsianum* RDR, DCL, and AGO families grouped clearly with known members that function in quelling or MSUD pathways (Fig 1B and 1D). RDR3, DCL1, and AGO1 grouped with quelling factors, while RDR1, DCL2, and AGO2 grouped with proteins from the MSUD pathway. RDR2 was identified in a separate group with RRP3 from *Neurospora crassa* and RDR4 from *C*. *parasitica* (Fig 1B), the function of which are not clear [44]. Expression of *RRP3* and *RDR4* is elevated by the introduction of dsRNA [45] or in response to viral infection [26], respectively.

Expression levels of *C*. *higginsianum* silencing genes were determined using quantitative real time PCR (qRT-PCR). Based on the phylogenetic analysis, we hypothesized that genes in the quelling and MSUD pathways would be expressed during asexual and sexual stages of fungal development, respectively. *Colletotrichum* is generally recognized as an asexual genus, although some species are able to adopt a sexual form, which are classified under the genus name *Glomerella* [33]. *Colletotrichum* asexual morphs are generally associated with disease symptoms, while sexual stages tend to develop in moribund or dead host tissues [33]. The *C*. *higginsianum* strain IMI349063A used in this study has not been observed to reproduce sexually [35], thus, gene expression analysis was limited to mycelia, conidia, and germinated conidia. *RDR3*, *DCL1* and *AGO1* had the highest levels of expression in mycelia (Fig 1E, left panel), conidia, and germinated conidia (S2 Fig, left panels), which is consistent with their predicted role in the quelling pathway during vegetative growth and asexual reproduction. Conversely, transcripts for *RDR1*, *DCL2* and AGO2 (MSUD pathway) were expressed at detectable but relatively low levels in mycelia, conidia, and germinated conidia (Figs 1E and S2, middle panels). Thus, while a role for *RDR1*, *DCL2* and *AGO2* during sexual reproduction is anticipated, it is possible that these genes have roles in asexual developmental phases. Transcripts for *RDR2* were expressed at intermediate levels in mycelia, but relatively high in conidia and germinated conidia (Fig 1E and S2 Fig, right panels).

### Loss of DCL1 or AGO1 leads to reduced conidiation and altered conidia morphology

**Fig 2:**
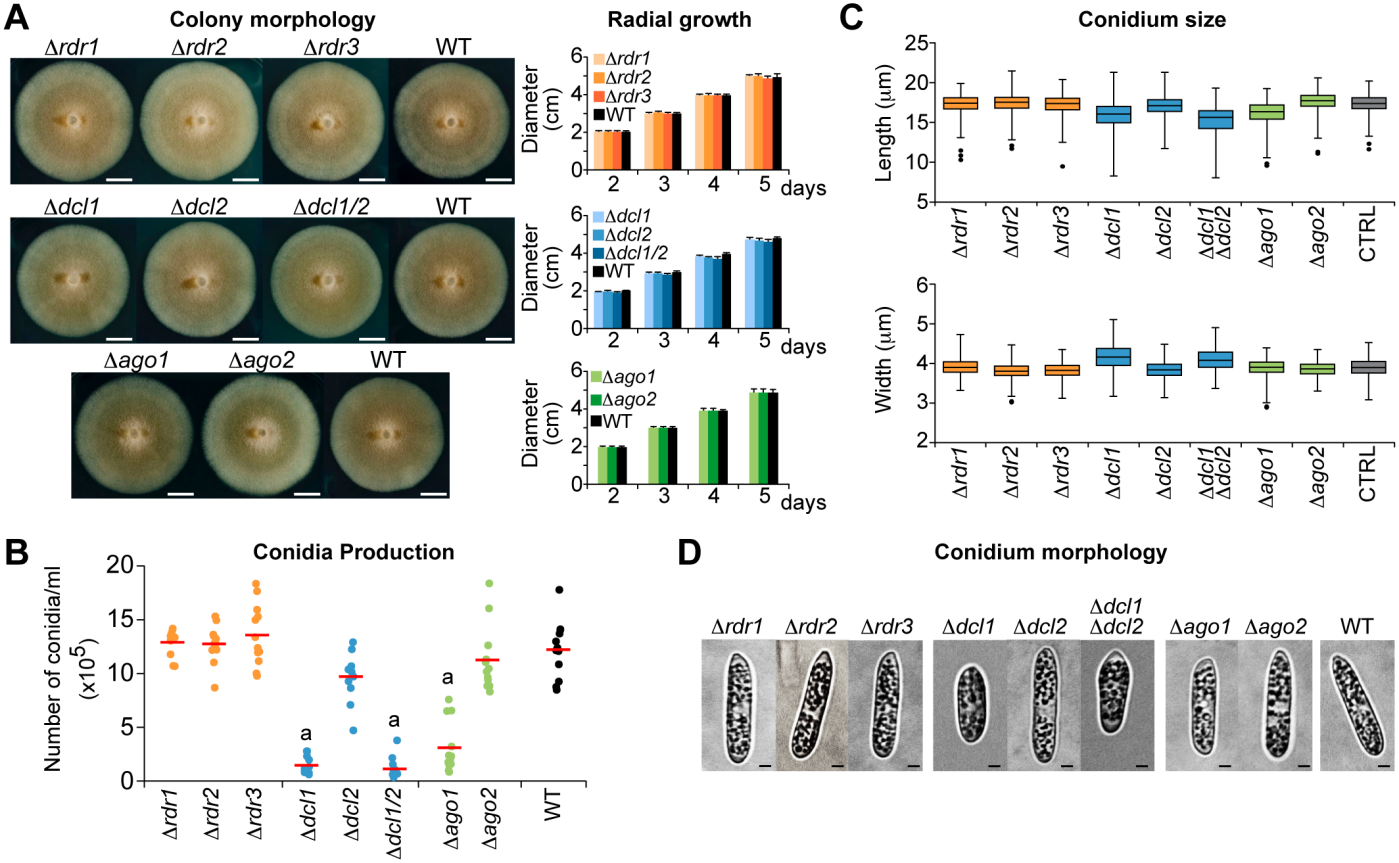
Phenotype analysis of *C*. *higginsianum* RNA silencing-mutant strains. (**A**) Colony morphology and radial growth phenotype for the *C*. *higginsianum* RNA silencing machinery mutants and Control strains on Mathur’s medium. A representative example of colony morphology after 6 days of growth (left panel), and radial growth measurements from days 2 to 5 (right panel) (mean +/- SE). Scale bar = 1 cm (**B**) Conidia production in the Control and RNA silencing mutant strains. Conidia were collected after 7 days of growth in Mathur’s medium and counted with a hemocytomer. Values plotted are from three biological replicates for each of four unique transformants; red bar indicates the mean. Strains with significantly different conidia production from the Control strain are indicated (“a”: p = 0.05). (**C**) Box plots representing length (upper panel) and width (lower panel) of conidia. Conidia were collected, observed and measured by light microscopy using a confocal microscope. At least 200 conidia were measured for each strain. Error bars represent the first and third quartile. The horizontal line within the box represents the median value (ie. 50th percentile). Black dots represent outliers. (**D**) Confocal images of conidia from the *C*. *higginsianum* Control and RNA silencing mutant strains. Scale bar = 2 μm.

To identify functions of the RNA silencing components in *C*. *higginsianum*, single deletion mutants with loss of *RDR* (S3 Fig), *DCL* (S4 Fig) and *AGO* (S5 Fig) genes were generated by targeted gene replacement. A double *DCL1*/*DCL2* deletion mutant was also generated (S4 Fig). Single-site gene disruptions were confirmed by Southern blot and RT-PCR analysis, and four independent deletion mutants were selected for further analysis (S3 and S5 Figs). Previous work in *Mucor circinelloides* [46], *Magnaporthe oryzae* [47], *Candida albicans* [48], *Botrytis cinerea* [12], and *Trichoderma atroviride* [13] demonstrated that disruptions of *DCL1* orthologs were sufficient to slow vegetative growth. However, in other fungi, such as *C*. *parasitica* [14] and *Saccharomyces castellii* [38], comparable mutations did not affect growth. To evaluate *C*. *higginsianum* silencing mutants, mycelial growth and morphology on conidiation (Mathur’s), complete (PDA), and minimal (CDA) media were measured. For all single and double mutants, colony morphology and growth were indistinguishable from that of the wild-type strain (Figs 2A and S6). The Δ*dcl1*, Δ*dcl2*, and Δ*dcl1*Δ*dcl2* mutants were also exposed to oxidative stress (H_2_O_2_ and Methyl Viologen), cell wall stress (Calcofluor white), salt-related stress (NaCl, and LiCl), osmotic stress (sorbitol, and sucrose) and nutrient deprivation (carbon and nitrogen). Growth of the Δ*dcl1*, Δ*dcl2*, and Δ*dcl1*Δ*dcl2* mutant strains was indistinguishable from that of the wild-type on all media (S7 Fig), suggesting that small RNA-mediated processes are not necessary for vegetative growth under several abiotic stress conditions. These results are in agreement with those reported for silencing mutants of the yeasts *S*. *castellii* [38] and *C*. *neoformans* [49].

Conidiation was analyzed for all mutant strains after growth in Mathur’s media for seven days. Compared to the control strains, conidiation was significantly reduced in the Δ*dcl1*, Δ*dcl1*Δ*dcl2*, and Δ*ago1* strains by 8.8-fold, 11.6-fold, and 4-fold, respectively (Fig 2B). There was no significant difference between the Δ*dcl1* and Δ*dcl1*Δ*dcl2* strains, indicating that conidiation effect was due to DCL1 with little or no redundancy with DCL2. *DCL* genes have been implicated in conidiation in other fungi [13,50]. In *B*. *cinerea*, at least partial redundancy of the two DCL proteins was observed [12] where the double mutant *dcl1dcl2* had a stronger, negative effect on sporulation than did the single mutants *dcl1* and *dcl2*. Argonautes were also shown to affect spore production in *M*. *circinelloides* [51], in which an *ago-1* mutant strain produced ~50% fewer spores than the control strain.

Conidia produced by the Δ*dcl1*, Δ*dcl1*Δ*dcl2*, and Δ*ago1* mutants were shorter and wider than those produced by the controls strain (Fig 2C). Most conidia from the Δ*dcl1* and Δ*dcl1*Δ*dcl2* strains exhibited an abnormal, slightly rounded morphology (Fig 2D), and were more variable in length and width than those control strains. Deletion mutants lacking *RDR1*, *RDR2*, or *RDR3* genes had no measurable growth or conidiation phenotypes (Fig 2B). However, it is not clear whether *RDR1*, *RDR2* and *RDR3* are not functional during growth and conidiation, or are functional but redundant, as combinatorial mutants were not analyzed.

### Loci not present in the *C*. *higginsianum* genome are a major source of small RNA and transcript RNA

**Fig 3:**
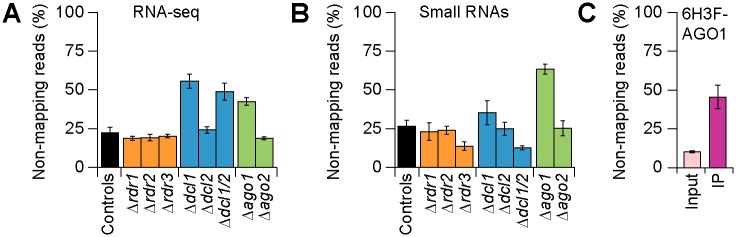
Transcript and small RNA reads unmapped to the *C*. *higginsianum* genome. Percentage of reads not aligned to the *C*. *higginsianum* genome in the Control and RNA silencing mutant strains, from (**A**) transcript and (**B**) small RNA libraries. (**C**) Percentage of small RNAs reads unaligned to the *C*. *higginsianum* genome from Δ*ago1*/6His-3FLAG-AGO1 Input and IP libraries. 6H3F, 6His-3FLAG.

The deletion mutant analysis revealed that DCL1 and AGO1 both affect conidia production and asexual spore morphology in *C*. *higginsianum*. To identify loci that are affected by RNA silencing factors, in particular DCL1 and AGO1, transcripts and small RNA from all mutant strains (Fig 3) were analyzed. In addition, small RNAs were analyzed after immunoprecipitation (IP) of 6His-3FLAG (6H3F) epitope-tagged versions of AGO1 (6H3F-AGO1) and AGO2 (6H3F-AGO2), which were expressed in Δ*ago1* and Δ*ago2* strains, respectively (S8 Fig). Transformants containing and expressing 6H3F-AGO1 had significantly increased conidia production relative to the original Δ*ago1* background, indicating the tagged version of AGO1 complemented the loss of *AGO1* (S8B and S8D Fig).

Transcript RNA reads were de-multiplexed and aligned to the *C*. *higginsianum* genome using Bowtie2 (S2 Table) [52]. Strikingly, 40±3% of the RNA-seq reads in Δ*ago1*, 54±5% of the reads in Δ*dcl1*, and 46±6% of the reads in Δ*dcl1*Δ*dcl2* did not map to the *C*. *higginsianum* genome (Fig 3A and S2 Table). Small RNA libraries were also de-multiplexed, trimmed of the 3’adaptor sequence, and aligned to the *C*. *higginsianum* genome using the aligner Bowtie (S3 Table) [53]. Here, 63±3% of the small RNA reads in Δ*ago1*, and 46±8% of AGO1-bound small RNAs did not map to the *C*. *higginsianum* genome (Fig 3B and 3C, S3 Table).

High-throughput sequencing usually yields a proportion of reads that do not map to the target genome such as sequencing errors, sequence variants, and chimeric sequences [54]. Draft genome sequences present further difficulties; the available *C*. *higginsianum* IMI349063A genome sequence contains approximately 48.2 Mbp of a predicted 53.4 Mbp nuclear genome across 10,235 contigs, where 5.2% of genes are split across multiple contigs, 4% of genes are truncated and the assembly does not include the mitochondrial genome [35]. Further, exogenous species (viral or bacterial) may be present in the target sample. Several infectious agents have been identified from high-throughput sequencing reads that did not map reference sequences [55–57]. Since the increase in unmapped transcript and small reads was specific to the Δ*dcl1*, Δ*dcl1*Δ*dcl2*, Δ*ago1* strains, as well as the small RNA reads from the 6F3H-AGO1 IP, we hypothesized that the *C*. *higginsianum* silencing machinery is important for the regulation of one or more loci of an undetermined origin.

### *C*. *higginsianum* strain IMI349063A is infected with a dsRNA mycovirus

#### Identification of unknown RNA molecules using *de novo* transcriptome assembly of RNA-seq data

To identify transcript(s) missing from the reference genome sequence we created a *de novo* transcript assembly using a combined set of all reads from the four Δ*dcl1* replicates and the program Trinity [58]. A total of 22,237 contigs were assembled with an average length of 509 bp per contig. From this assembly 26 non-redundant contigs were identified as originating from the *C*. *higginsianum* mitochondrial genome. Additionally, a 4,077 bp contig was identified as an additional copy of the large subunit rRNA gene. The mitochondrial and rRNA transcript sequences were added to the existing *C*. *higginsianum* genome reference sequence for subsequent whole-genome analyses (S1 Text).

**Fig 4:**
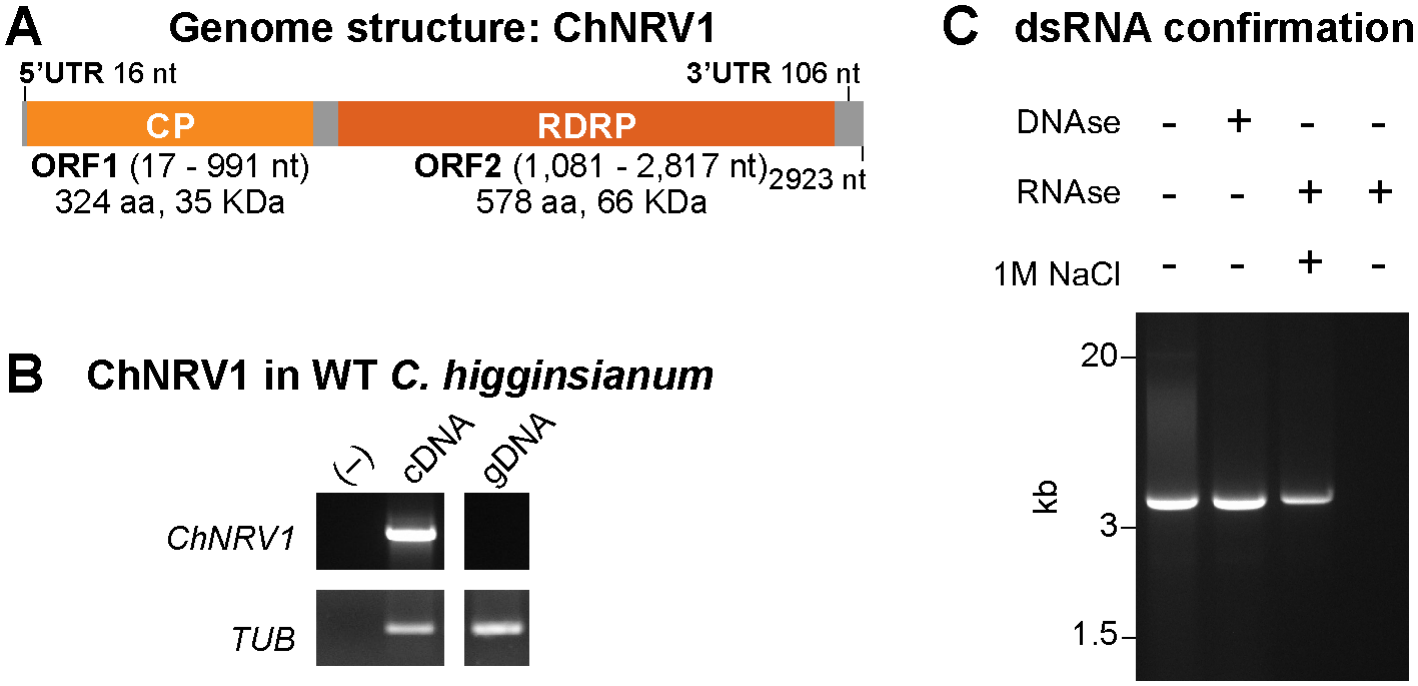
Identification of a novel dsRNA virus in *C*. *higginsianum*. (**A**) Genome organization and size of the *C* *olletotrichum* *h* *igginsianum* Non-segmented dsRNA Virus 1 (ChNRV1) identified by *de novo* transcriptome assembly. ChNRV1 contains two ORF that are in different frames. ORF1 encodes a putative coat protein (CP) and ORF2 a putative RNA-dependent RNA polymerase (RdRP). UTR, Untranslated region. (**B**) Accumulation of *ChNRV1* transcripts in the *C*. *higginsianum* wild-type strain IMI 349063A as determined by semi-quantitative RT-PCR analysis (center lane, cDNA), using primers spanning both ORFs (S7 Table). PCR analysis from genomic DNA (gDNA) of *C*. *higginsianum* IMI 349063A strain using the same primers pairs determined that ChNRV1 is not integrated into the fungal genome. (**C**) Electrophoretic analysis of viral dsRNA in 1% agarose gel without treatment, treated with DNase, treated with RNase in a high-salt, or treated with RNase in a low salt buffer. Resistance to degradation by RNaseA in buffer with high salt concentration confirmed the nature of the dsRNA molecule.

Next, the unmapped RNA-seq reads from one Δ*dcl1* replicate were aligned to the *de novo* assembled transcriptome to identify high coverage transcripts not present in the genome sequence. A single sequence, assembled into two contigs representing the forward and reverse strand of the same sequence, accounted for 79% of the previously unaligned reads. A BLASTX analysis against the nr database using the longer of these two sequences (2,835 bp versus 2,611 bp) identified two putative open reading frames (ORF) (Fig 4A). The highest-scoring alignment for the 5’ ORF (ORF1) was ORFA, a protein of unknown function from *Beauveria bassiana* RNA virus 1 (e-value 0.0). The best alignment for the 3’ ORF (ORF2) was to the RNA-dependent RNA polymerase of *Penicillium janczewskii Beauveria bassiana-like* virus 1 (e-value 0.0). The conserved domain cd01699 (RNA-dependent RNA polymerase) was also identified.

#### Novel RNA molecule is a double-stranded RNA virus present in the wild-type strain

Both bioinformatic and molecular approaches were used to confirm the presence of the virus in wild-type *C*. *higginsianum* and was thus ancestral to the generated RNA silencing mutant lines. RNA-seq reads from the control samples were aligned to the Trinity contigs, resulting in 9x coverage of both virus genome contigs. Additionally, we analyzed previously published RNA-seq data from four developmental stages of *C*. *higginsianum* [35] for the presence of the virus and found sequences that uniquely mapped along 95% of the viral genome. Normalized virus read counts (per million mapped) for the O’Connell and colleagues [35] dataset were approximately 300-times less abundant than observed in our control dataset, likely due to differences in total RNA extraction protocols: O’Connell and colleagues used poly-A purified total RNA as the template for library preparation, while we used rRNA-depleted RNA. RT-PCR demonstrated the presence of a viral transcript in the wild-type strain of *C*. *higginsianum* (IMI349063A) (Fig 4B, Lane 2), and PCR analysis of fungal genomic DNA showed that the sequence was not integrated into the *C*. *higginsianum* genome (Fig 4B, Lane 3). Further, the RNA molecule was determined to be dsRNA based on resistance to DNase, resistance to RNase at high salt concentration (1 M NaCl), and degradation by RNase at low salt concentration (Fig 4C).

RNA-ligase-mediated RACE (RLM-RACE) [59] sequencing extended the 5’ and 3’ ends of the coding strand by 65 nt and 23 nt, respectively, resulting in a complete dsRNA molecule of 2,923 nt (Fig 4A). The additional 5’ sequence extended ORF1 to include an ATG start codon, resulting in a 975 nt long gene, putatively encoding a 324 aa (~35 kDa) protein. The dsRNA sequence included 5’ and 3’ untranslated regions (UTR) of 16 and 106 nt respectively. ORF1 and ORF2 are in different frames of the plus strand: ORF1 in frame 2 (nucleotides 17 to 991) and ORF2 in frame 1 (nucleotides 1081 to 2817), with an 89-nt spacer in between. A putative slippery site heptamer, G GAU UUU, is present immediately upstream of the stop codon of ORF1, suggesting that an ORF1-ORF2 fusion protein may be produced by a -1 ribosomal frameshift.

As a search through the *de novo* Trinity transcripts did not reveal any additional viral RNAs, and the dsRNA sequence was assembled as a single fragment, we have designated the assembled contig as *C* *olletotrichum* *h* *igginsianum* non-segmented dsRNA virus 1 (ChNRV1). The complete viral genome sequence is deposited in GenBank under the accession KM923925, and GenBank protein IDs for ORF1 and ORF2 are AIW81424 and AIW81425, respectively.

The identification of ChNRV1 prompted us to ask if it was present in other isolates of *C*. *higginsianum* and the closely related species *C*. *destructivum*, collected from various geographical locations (S4 Table). RT-PCR was used to screen for the presence of ChNRV1 and only one other *C*. *higginsianum* strain (IMI349061), which was collected from Trinidad and Tobago along with the strain used in this study, had detectable virus (S4 Table). As this assay specifically queried for the presence of ChNRV1 sequences, these *C*. *destructivum/higginsianum* strains could be infected with other viruses. Viral dsRNA elements have been described previously in three *Colletotrichum* species, including one further characterized as a Gammapartitivirus [60–62]. Deeper surveys of more strains from additional locations will be needed to fully define the range of ChNRV1.

#### Analysis of ChNRV1 proteins and virions

**Fig 5:**
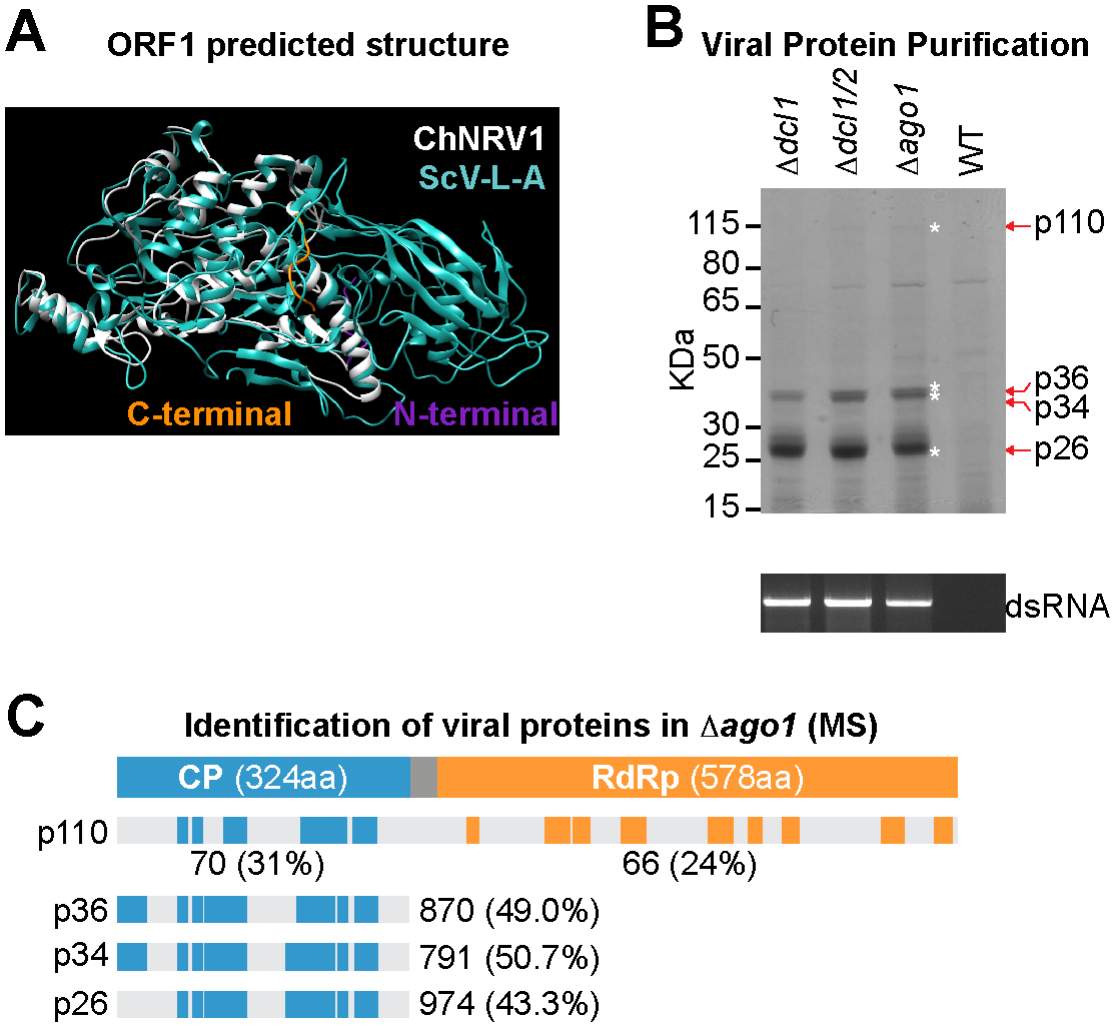
Characterization of viral proteins. (**A**) Predicted secondary structure of ChNRV1-ORF1, putative coat protein, (white) aligned to the model of ScV-L-A capsid protein (cyan). The 10 amino acids from the N-terminal and C-terminal ends of ChNRV1-ORF1 are in purple and orange respectively. (**B**) Analysis of viral proteins by SDS-PAGE (upper panel) and dsRNA by agarose electrophoresis (lower panel) from purified virus fractions. Four proteins bands (p110, p36, p34 and p25) and viral dsRNA accumulate in the Δ*dcl1*, Δ*dcl1*Δ*dcl2* and Δ*ago1* strains. KDa, kilodaltons. (**C**) Distribution of ChNRV1 trypsin-digested p110, p36, p34 and p26 peptides identified by Mass Spectrometry along the capsid protein (blue boxes) and the RdRP protein (orange boxes). Values indicate the mean normalized spectral counts and the percentage of sequence coverage in parenthesis, p110: below diagram; p36, p34, p26: next to diagram.

Double-stranded RNA viruses share numerous structural and functional similarities [63]; many are encapsidated with the necessary capsid proteins encoded for within their genome, along with the RdRP used for replication. Therefore, we hypothesized that ORF1 encodes a capsid protein. Using the previously solved 3D structure of *Saccharomyces cerevisiae* virus L-A (ScV-L-A) (PDB ID: 1m1c) [64] to predict the putative 3D structure of ORF1 using I-TASSER [65,66]. The template modeling score (TM-score) is reported between 0 and 1, where a TM-score greater than 0.5 indicates a strong similarity in topology [67,68]. The TM-score for ORF1 aligned to ScV-L-A capsid protein was 0.872 indicating that the two proteins have a similar topology [67]. The next highest scoring structural analog was the capsid protein from *Penicillium chrysogenum* virus (PDB ID: 3j3iA) with a TM-score of 0.594. The top model predicted by I-TASSER was downloaded and visualized with Chimera, where the ORF1 sequence is in white and the ScV-L-A capsid sequence in cyan (Fig 5A and S1 Movie). As ScV-L-A capsid protein sequence is ~2X longer than ORF1 (608 aa versus 324 aa), it was expected that a large part of the ScV-L-A capsid sequence would not have homologous regions in ORF1. Indeed, ORF1 is structurally similar to only the N-terminal 414 aa of ScV-L-A. This N-terminal region is enriched with alpha helices (56%) compared to full length ScV-L-A (38%) while the C-terminal end, which is not found in ORF1, is enriched in beta sheets (C-terminus: 86%, entire sequence: 63%) (S9 Fig). Thus ORF1 appears to contain the conserved helix-rich core found in ScV-L-A and other totiviruses [69].

SDS-polyacrylamide gel electrophoresis (SDS-PAGE) analysis detected differential accumulation of four proteins of approximately 110, 36, 34, and 26 KDa in the Δ*dcl1*, Δ*dcl1*Δ*dcl2* and Δ*ago1* strains that were visibly absent in the wild-type strain, that was accompanied by specific accumulation of dsRNA molecules (Fig 5B). Each protein was excised from two biological replicates of Δ*ago1* and wild-type strains, digested by trypsin and subjected to mass spectrometry (MS). Protein abundance was estimated on the basis of spectral count values [70] (S5 Table). In all Δ*ago1* samples, proteins encoded by ChNRV1 were the most abundant proteins observed. From the p110 sample, an average of 70 and 66 spectral counts matched the capsid and RdRP proteins, respectively (Figs 5C and S10A, S5 Table). The estimated size of p110 is similar to the predicted size of a CP-RdRP fusion protein (933 aa, 103.9 KDa), suggesting that these proteins are expressed as a single polyprotein, likely due to a ribosomal frameshift. However as the predicted m/z ratio of the trypsin products from the frameshift region exceeded the MS survey scan m/z cut-off, these fragments were not observed. The capsid protein was the major source of spectral counts in the Δ*ago1* mutant for the p36, p34, and p26 samples (870, 791, and 970, counts respectively) (Figs 5C and S10A, S5 Table). Both p36 and p34, but not p26, had trypsin-derived peptides that matched the capsid N-terminus (Figs 5C and S10C), suggesting that p26 is a truncated form of the capsid protein. Although not visibly detectable on the gel, the capsid protein was detected by MS in all wild-type samples (10–33 spectral counts) (S5 Table), confirming that ChNRV1 capsid protein is present at very low amounts in *C*. *higginsianum* wild-type.

We attempted to purify viral particles from mycelia of the Δ*dcl1*, Δ*dcl1*Δ*dcl2*, and Δ*ago1* strains, however, despite highly abundant dsRNA and capsid protein in the viral particle preparation, repeated attempts to observe ChNRV1 virions using electron microscopy were unsuccessful. Most dsRNA viruses assemble their capsid by arranging 120 capsid proteins, organized into 60 dimers, thus, dimer formation is a key step prior capsid assembly [71]. ß-sheets are located at the dimer interface and are important for dimer stabilization in members of the *Partitiviridae* family [72,73]. The putative capsid proteins encoded by ChNRV1 and plant amalgamaviruses are shorter in size than those from the *Totiviridae* and *Partitiviridae* families and have fewer ß-sheets (S9 Fig). Thus ChNRV1 may not form classical virions, similar to members of the plant *Amalgamaviridae* family [74–76]. Virions are an effective means of protecting the viral dsRNA genome from the host RNA silencing machinery, but in the absence of a viral particle, specialized structures within the cell may be used. Members of the *Hypoviridae* family enclose their genome within host-derived vesicles [18,77]. Therefore, it remains to be determined if ChNRV1 capsid is functionally required for dsRNA protection. Further microscopic analyses of *C*. *higginsianum* mycelium combined with viral-specific antibodies may reveal the presence of ChNRV1-containing structures.

### ChNRV1 belongs to a distinct group of dsRNA viruses

**Fig 6:**
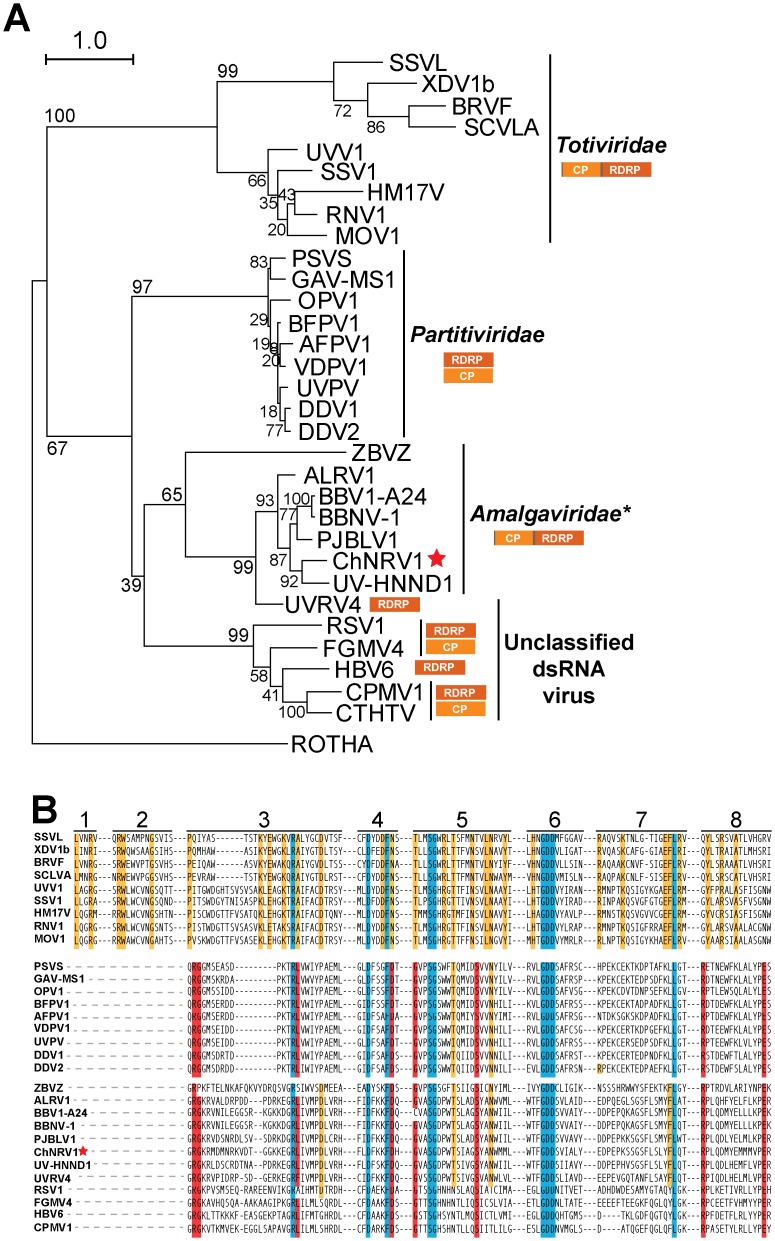
Phylogenetic and sequence analysis of ChNRV1-RDRP. (**A**) A phylogram showing the relationship of the RdRP of selected mycoviruses from the *Totiviridae* (non-segmented genome), *Partitiviridae* (Bipartite genome) and Unclassified dsRNA viruses (Monopartite and Bipartite genome) families; sequence from human Rotavirus A (ROTHA) serves as the outgroup. ChNRV1 is indicated with a red star. Virus names have been abbreviated; full names and accession numbers for protein sequences used in the alignment are in S6 Table. Phylogenetic trees were generated using Raxml under the model LG+G+F of amino acid substitution. Scale bar in each panel represents 0.5 amino acid substitutions per site. RDRP, RNA dependent RNA polymerase; CP, coat protein. (**B**) MAFFT amino acid sequence alignment of the conserved motifs of RdRP of ChNRV1 and the dsRNA Mycoviruses used in (A). Numbers at the top indicate the eight conserved domains from dsRNA viruses of lower eukaryotes [78]. Residues found in all viral sequences (top and lower panels) are shaded in blue. Residues specific to *Totiviridae*- or *Partitiviridae*/Unclassified are shaded in yellow and red, respectively.

To determine the relationship between ChNRV1 and other mycoviruses we used a maximum-likelihood phylogenetic analysis on the amino acid sequence of the RdRP of ChNRV1 and other mycoviruses from the *Totiviridae*, *Partitiviridae*, and Unclassified dsRNA viruses (S6 Table). We initially hypothesized that ChNRV1 is a *Totiviridae* based on its non-segmented genome organization and the predicated structural similarity between ORF1 and the capsid protein of ScV-L-A. However ChNRV1 was not placed into either the monophyletic *Totiviridae* or the *Partitiviridae* groups (Fig 6A). Instead, ChNRV1 grouped in as a sister clade to the *Partitiviridae* family RdRPs with a number of recently described members of the family *Amalgaviridae* that primarily have monopartite genomes (Fig 6A). A set of RdRP sequences from dsRNA viruses currently unclassified is also part of this sister clade, but separate from the *Amalgaviridae* members. Further supporting a relationship with *Partitiviridae* RdRPs, an analysis within the eight conserved domains of RdRP proteins of dsRNA viruses determined that all residues specific to the *Partitiviridae* family RdRPs are present in ChNRV1, while only 4 of 29 *Totiviridae*-specific residues are observed [78] (Fig 6B).

While the evolutionary relationship between ChNRV1 and existing mycovirus families is unclear, it seems likely that ChNRV1 belongs to a distinct group of dsRNA viruses with some similarities to both totiviruses and partitiviruses. The recent discovery of both fungal and plant dsRNA viruses with high levels of shared sequence homology indicates these viruses are either new members of the *Amalgamaviridae* family or belong to a yet to be determined family [74–76,79–84] (Fig 6A).

### ChNRV1 is a target of the *C*. *higginsianum* RNA silencing machinery

#### ChNRV1 transcripts and dsRNA genome are affected by AGO1 and DCL1

**Fig 7:**
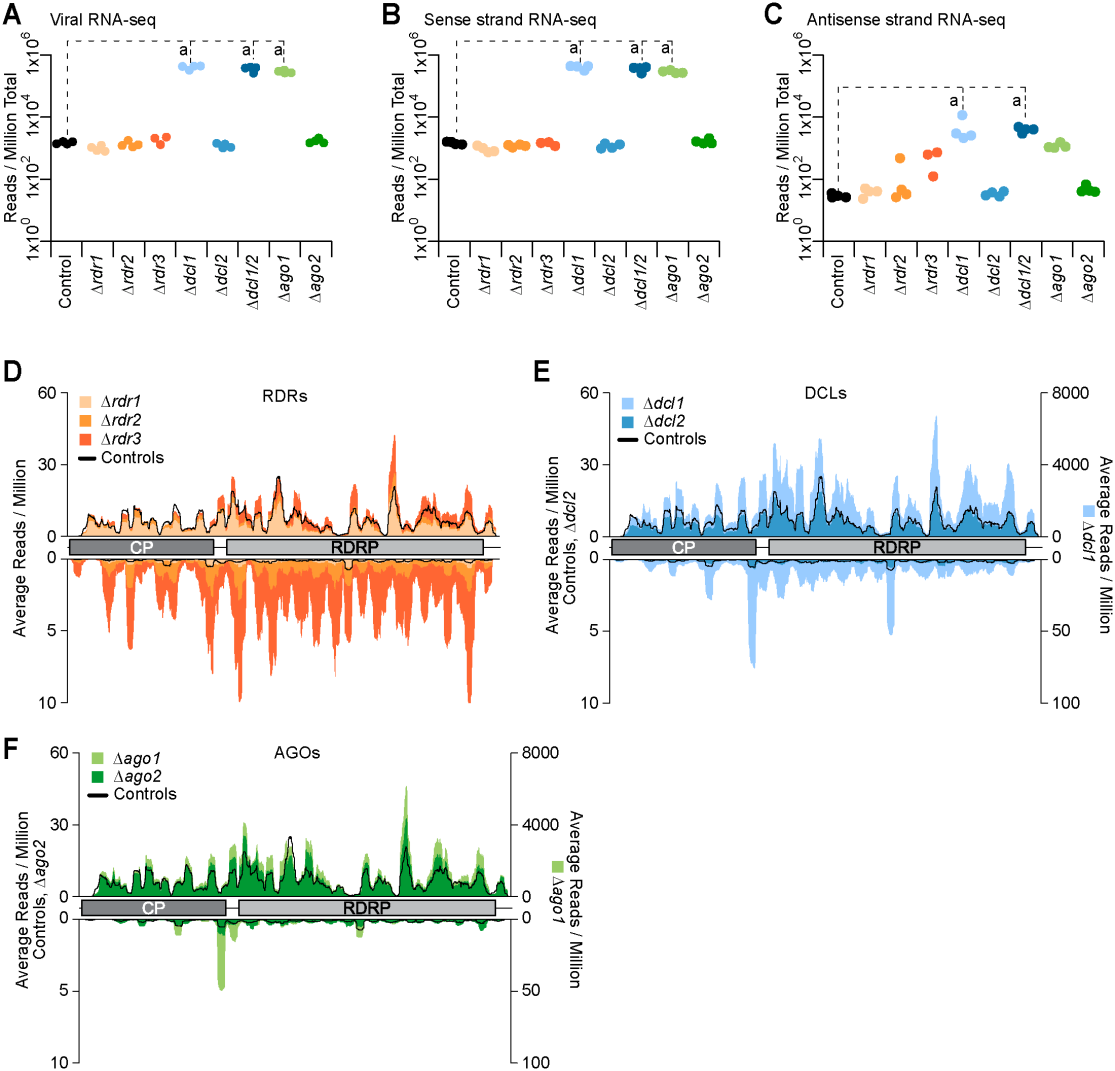
Analysis of RNA-seq reads mapped to ChNRV1. (**A**) RNA-seq reads mapped to the virus genome were scaled to reads per million of total reads (RPM) for each library. Significant differences between Control and RNA silencing mutant strains are indicated where “a”: p = 0.05. (**B**) RNA-seq reads mapped to the sense strand of the virus, scaled to RPM. Significant differences between Control and RNA silencing mutant strains are indicated where “a”: p = 0.05. (**C**) RNA-seq reads mapped to the antisense strand of the virus, scaled to RPM. Significant differences between Control and RNA silencing mutant strains are indicated where “a”: p = 0.05. (**D-F**) RNA-seq reads plotted along the viral genome (sense, above x-axis; antisense, below x-axis). Average reads per million reads mapped are on the y-axis; left y-axis scale for the sense strand (0–60) and antisense strand (0–10) for Δ*rdr1*, Δ*rdr2*, Δ*rdr3*, Δ*dcl2*, Δ*ago2* and Controls; for Δ*dcl1* and Δ*ago1* the right y-axis scale for the sense strand (0–8,000) and antisense strand (0–100) is indicated.

Since ChNRV1 RNA and protein levels changed in specific mutant strains (Fig 5B), we hypothesized that ChNRV1 is regulated by RNA silencing. Transcript and small RNA datasets from the mutant and control strains were re-aligned to a reference genome, which included the *C*. *higginsianum* supercontigs along with the mtRNA, rRNA, and ChNRV1 sequences. Using this updated reference genome resulted in an increase in RNA-seq reads mapped in comparison to the original genome alignments (S11A Fig). The Δ*dcl1*, Δ*dcl1*Δ*dcl2*, and Δ*ago1* strains had the largest gain in mapped reads with an average of 2.1X, 1.8X, and 1.7X more mapped reads, respectively. RNA-seq reads mapped to the virus were >80% of the newly mapped reads in Δ*dcl1*, Δ*dcl1*Δ*dcl2*, and Δ*ago1*, but less than 3% of the remaining genotypes (S11A Fig). The total number of reads mapped to the ChNRV1 genome was determined for all replicates and scaled by the total number of reads in the respective library (Fig 7A). A one-way ANOVA and Tukey post-hoc analysis evaluated the differences in scaled read counts between the mutant and controls strains. Only the Δ*dcl1*, Δ*dcl1*Δ*dcl2*, and Δ*ago1* strains had significantly differing amounts of RNA-seq reads than the controls with 411,802 more reads in Δ*dcl1* (95% confidence interval: 294,782 to 528,823), 563,122 more reads in Δ*dcl1*Δ*dcl2* (95% confidence interval: 446,102 to 680,143) and 290,788 more reads in Δ*ago1* (95% confidence interval: 173,767 to 407,808) (Fig 7A).

Next we analyzed the number of reads that mapped to the ChNRV1 sense and antisense strands, hypothesizing that a significant increase in virus transcription would result in a greater amount of sense strand (mRNA). Indeed, the scaled read counts mapped to the sense strand of the virus genome were significantly greater in the Δ*dcl1*, Δ*dcl1*Δ*dcl2*, and Δ*ago1* strains relative to the controls, with 406,981, 556,991, and 289,613 more reads, respectively (Fig 7B and 7D–7F) (95% confidence intervals Δ*dcl1*: 289,234 to 524,729; Δ*dcl1*Δ*dcl2*: 439,244 to 674,739; Δ*ago1*: 171,866 to 407,361). Quantitative RT-PCR confirmed the RNA-seq results as a substantial increase in viral mRNA levels in the Δ*dcl1*, Δ*dcl1*Δ*dcl2*, and Δ*ago1* strains, while the remaining strains had levels of *ChNRV1* mRNA near wild-type (S12A Fig). A significant difference was also observed in the number of reads mapped to the antisense strand of the ChNRV1 genome in Δ*dcl1* and Δ*dcl1*Δ*dcl2* (Fig 7C–7F), where 4,820 and 6,130 more reads mapped, respectively (95% confidence intervals, Δ*dcl1*: 995 to 8,645; Δ*dcl1*Δ*dcl2*: 2,306 to 9,955). Antisense strand reads were also increased in the Δ*ago1* strain but did not meet the threshold of significance. The presence of RNA-seq reads from the antisense strand of the ChNRV1 genome serve as an indicator for the dsRNA genome and correspond with the accumulation of viral dsRNA elements in the Δ*dcl1*, Δ*dcl1*Δ*dcl2*, and Δ*ago1* strains observed during gel electrophoresis (Fig 5B). Despite using RNA preparation methods not optimized for isolation of dsRNA species, antisense reads from the dsRNA genome would be expected to be at least partially amplified by our library preparation protocol; Coetzee and colleagues identified the dsRNA “virome” of an infected vineyard by treating dsRNA at 95°C for 10 minutes, followed by fragmentation via the Illumina mRNA Sequencing Kit [85], which are conditions similar to those employed in our study. A significant effect on viral RNAs was not observed in the RNA-seq data from any of the single RDR mutants. Thus ChNRV1 is deregulated in the Δ*dcl1*, Δ*dcl1*Δ*dcl2*, and Δ*ago1* strains relative to wild-type, resulting in the accumulation of both dsRNAs and mRNAs (Figs 5, 7 and S12A).

#### Small RNAs derived from ChNRV1 are DCL1-dependent

**Fig 8:**
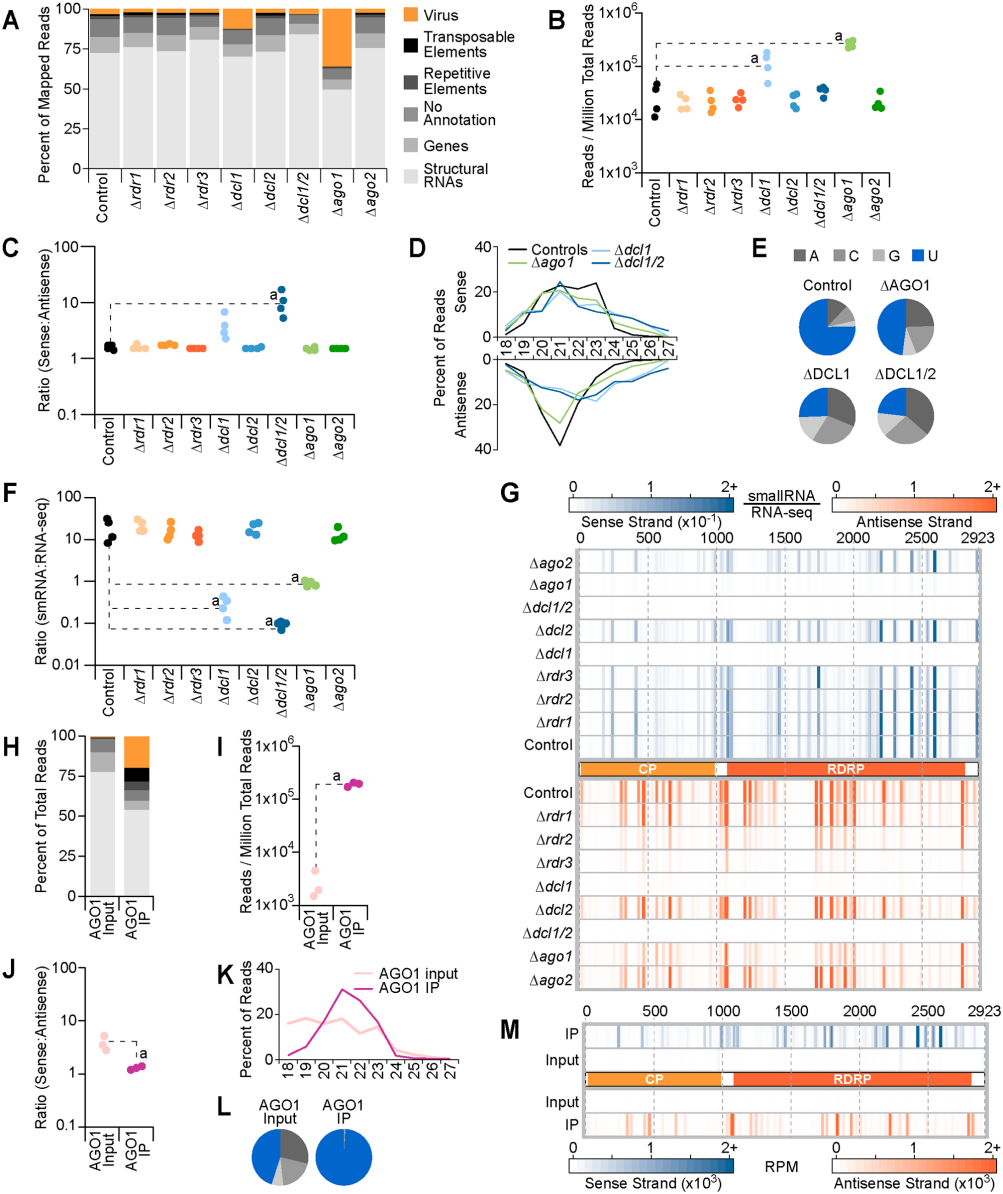
Characterization of viral small RNAs. (**A-G**): Analysis of viral small RNAs from the Controls and RNA silencing mutant strains. Significant differences between the Control and RNA silencing mutant strains are indicated where “a”: p < 0.05. (**A**) Summary of genomic loci that produce small RNAs as a percentage of total reads per genotype. (**B**) Small RNA reads, scaled to reads per million of total reads (RPM), mapped to the virus genome. (**C**) Ratio of sense to antisense small RNAs that mapped to the viral genome. (**D**) Distribution of viral small RNAs by size and by strand as a percentage of total viral small RNAs by strand. Sense strand small RNAs are plotted above the x-axis and reads from the antisense strand are plotted below. (**E**) 5’ nucleotide distribution of viral small RNAs. (**F**) Ratio of small RNAs to RNA-seq mapped to ChNRV1. RPM counts for each small RNA replicate were divided by the average RPM counts from the RNA-seq for that genotype. (**G**) Analysis of small RNA read counts along the viral genome, as a function of RNA-seq levels. Small RNA RPM counts per nucleotide were determined by strand then divided by the average RPM counts for the RNA-seq for that genotype and strand. Graphical representation of the virus is in the middle, with the two ORFs indicated, and genome coordinates are along the top-most edge. Sense strand reads are plotted in blue above the genome figure and antisense reads are plotted in orange below. The scale for sense strand and antisense strand values is shown as density heatmaps above the plot. (**H-M**): Analysis of the 6H3F-AGO1 input and IP fractions. Significant differences between the Input and IP fractions are indicated where “a”: p < 0.05. (**H**) Summary of genomic loci that produce small RNAs found in the input and IP fractions. Colors are the same as (A). (**I**) RPM counts of small RNAs in the input and IP that mapped to the virus genome. (**J)** Ratio of sense to antisense of viral small RNAs. (**K**) Size distribution and (**L**) 5’ nucleotide distribution of viral small RNAs as a percentage of total viral small RNAs. (**M**) Input and IP RPM counts plotted by strand at single nucleotide resolution along the virus genome. Heatmap densities shown below the plots indicate the scale of RPM for sense and antisense strand reads.

In other organisms, induction of an antiviral defense response is generally characterized by the production of small RNA molecules that map to the sense and/or antisense strand of the viral genome [86–88]. Thus, small RNAs were aligned to the new reference genome, using Bowtie and allowing only perfect matches. As with the RNA-seq data sets, all strains demonstrated an increase in the percent of reads mapped compared to the original mapping results (S11C Fig). ChNRV1-derived small RNAs were present in all strains analyzed (Figs 8A and S11D), along with other genomic features including structural RNAs, protein-coding genes, repeats, transposable elements, and non-annotated regions (Fig 8A). Aligning against the expanded genomic sequence, Δ*ago1* gained the greatest amount of reads (29%) where >90% of these reads mapped to ChNRV1. While not an abundant proportion of total reads, ChNVR1-derived small RNAs also represented the largest category of newly mapped reads in Δ*rdr3*, Δ*dcl1*, and Δ*dcl1*Δ*dcl2* (S11D Fig).

ChNRV1-derived small RNAs were scaled to the total number of small RNAs per library then evaluated using a one-way ANOVA and Tukey post-hoc analysis. Due to the significant increase in ChNRV1 levels observed in the Δ*ago1* RNA-seq dataset, we hypothesized that viral small RNA production may be lower in the Δ*ago1* strains. However, a significant increase in viral small RNAs was observed the Δ*ago1* mutant strain relative to the controls, with 234,333 more reads present in Δ*ago1* (Fig 8B) (95% confidence interval: 174,481 to 294,185). This result indicates that AGO1 is not required for production of ChNRV1-derived small RNAs, and that in the absence of AGO1, ChNRV1 was not adequately targeted for suppression. Further, the abundant production of small RNAs in Δ*ago1* demonstrated that DICER activity alone is insufficient for controlling viral infection, a result also observed in an *ago1/ago2* double mutant of *Arabidopsis thaliana* where DCL4 activity alone was insufficient for controlling viral infection [89]. We further hypothesized that without DCL1, viral small RNAs would be significantly under-represented. However, ChNRV1-derived small RNAs were significantly over-represented in Δ*dcl1* relative to the controls (Fig 8B) (89,571 more reads; 95% confidence interval: 29,719 to 149,423), while levels in the Δ*dcl1*Δ*dcl2* strain were not significantly different than the controls.

To address the unexpected results for the Δ*dcl1* and Δ*dcl1*Δ*dcl2* samples we examined the ratio of sense to antisense small RNAs from the viral genome, hypothesizing that if the sequences observed in these samples were true small RNAs, this ratio would remain unchanged relative to the controls. A clear strand bias was not observed in the *C*. *higginsianum* control strains (1.48 sense to antisense), indicating that viral small RNAs originate from both sense and antisense strands in an RNA silencing-proficient strain (Fig 8C). The ratio of sense to antisense reads in all the *rdr* and *ago* mutants, as well as Δ*dcl2* strain, was indistinguishable from the controls. Further, while the total number of viral small RNAs significantly increased in Δ*ago1*, the relative ratio of sense to antisense reads was maintained, indicating that DCL action remained capable of generating viral small RNAs in the absence of AGO1. However, the average ratio of sense to antisense small RNAs in Δ*dcl1* and Δ*dcl1*Δ*dcl2* strains was 3.7 and 9.5, respectively (Fig 8C), where the higher ratios are due to an increase in the number of reads from the sense strand (data available in Fig 8 dataset). As this ratio was only partially perturbed in the Δ*dcl1* strain, possibly DCL2 activity is capable of producing antisense viral small RNAs in the absence of DCL1; the loss of both DCLs results in primarily sense strand small RNA sequences with low representation from the antisense strand.

To further characterize the nature of ChNRV1-derived small RNAs, particularly those in the DCL1-deficient strains, we did a routine analysis of small RNAs by size and 5’ nucleotide preference. An initial analysis of the small RNAs by size did not reveal a pattern responsive to the loss of *DCL1* (data available in Fig 8 dataset). Thus, viral small RNAs were split by strand and then plotted by size, reasoning that the antisense strand reads would convey the size profile of true viral small RNAs, while the signal on the plus strand may be contaminated with viral mRNA degradation products. The size distribution of ChNRV1 sense strand small RNAs was broad (20–24 nt) and largely unaffected by genotype (Figs 8D and S13A). In particularly the size distribution by strand for the Δ*ago2*, Δ*dcl2*, and all *rdr* mutant strains was indistinguishable from the controls, with peak abundance at 21 nt on the antisense strand (S13A Fig). Analysis of antisense strand small RNAs in the Δ*ago1* strain also demonstrates a peak at 21 nt, along with a slight increase in abundance of longer (≥23 nt) small RNAs (Fig 8D). A more pronounced shift in size is observed in the two strains lacking *DCL1*, where 21 nt sequences are depleted and those ≥ 23 nt are more abundant (Fig 8D). This suggests that despite the presence of antisense small RNAs in the Δ*dcl1* strains, there was a loss in size-specificity expected of DICER-dependent small RNAs. A Northern blot analysis of visRNAs from Δ*dcl1*, Δ*dcl1*Δ*dcl2*, Δ*ago1*, and wild-type confirmed three key aspects of the sequencing analyses (S12B Fig). First, viral small RNAs in Δ*ago1* are more abundant than in wild-type, and that there is a size specificity of ~22 nt. Secondly, the signal in the Δ*dcl1* lanes was diffuse and lacked the specific accumulation of visRNAs observed in the Δ*ago1* strains. Finally, no signal from ChNRV1 small RNAs was detected in the Δ*dcl1*Δ*dcl2* or wild-type lanes indicating that accumulation in these strains was demonstrably lower than observed in the Δ*ago1* strains, and indeed, below the threshold for detection via blot under these experimental conditions.

ChNRV1-derived small RNAs from the control strains had a preference for 5’ U, which was negatively affected in the Δ*dcl1*, Δ*dcl1*Δ*dcl2*, and Δ*ago1* strains (Fig 8E), but not in the remaining RNA silencing mutants (S13B Fig). In Δ*ago1* less than 50% of reads had a 5’U compared to 75% in the controls, suggesting that AGO1 binds to and stabilizes viral small RNAs with a 5’U. Similarly, loss of DCL1 affected the 5’ nucleotide distribution of ChNRV1 small RNAs, where only 25% of reads contained a 5’U (Fig 8E). Thus, size and nucleotide analyses indicate that in strains with an intact AGO1 and DCL1, ChNRV1-derived small RNAs are 21-nt in length with a 5’U.

To better understand viral small RNA production, particularly in the Δ*dcl1* and Δ*dcl1*Δ*dcl2* strains, we measured the abundance of ChNRV1-derived small RNAs relative to the ChNRV1 RNA-seq data. Each replicate of small RNA for each genotype shown in Fig 8B was divided by the average number of RNA-seq reads of the corresponding strain (data shown in Fig 7A). The average ratio of small RNAs to RNA-seq in the controls was 23.33, which was not significantly different from that of the *rdr*, *dcl2*, and *ago2* mutant strains (Fig 8F). The ratio observed in Δ*ago1* was significantly lower at 0.90 (95% confidence interval: 4.0 to 34.1), meaning that small RNA production from ChNRV1, as a function of the RNA-seq data, was approximately 22 times lower in Δ*ago1* (Fig 8F), further indicating that the abundant levels of ChNRV1 small RNAs produced by DCL1 are not sufficient for controlling viral RNA levels; AGO1 is also required. As with Δ*ago1*, the small RNA to RNA-seq ratios observed in Δ*dcl1* and Δ*dcl1*Δ*dcl2* decreased to a mean of 0.28 and 0.06, respectively (95% confidence intervals: 4.6 to 34.7 and 4.8 to 34.9). Visualizing the distribution of small RNAs along the viral genome, again controlling for RNA-seq levels, illustrates that particular loci from both strands appeared to be hot spots for ChNRV1-derived small RNA production in strains with a functioning DCL1 and AGO1 (Fig 8G). Taken together, these results indicate that DCL1 is the primary producer of viral small RNAs as the strains lacking this gene have altered size and 5’ nucleotide distributions relative to RNA silencing-proficient strains. Further, AGO1 is also a crucial member of the antiviral defense as DCL1 activity alone was inadequate for controlling ChNRV1 transcript levels.

#### Small RNAs derived from ChNRV1 are loaded into AGO1

ChNRV1-derived small RNAs were further characterized by the analysis of AGO immunoprecipitate fractions. A comparison of unique sequences in the 6H3F-AGO2 IP and WT/mock IP revealed a high degree of correlation between these samples (average Pearson correlation co-efficient (ρ) = 0.96) that was not observed between 6H3F-AGO1 IP and WT/mock IP (average ρ = 0.67). Combined with the nearly undetectable levels of AGO2 RNA and protein, suggested that AGO2 does not play a major role in RNA silencing during vegetative growth. As such, we focused all further analyses on the 6H3F-AGO1 input and IP samples only. As expected, we observed ChNRV1-derived small RNAs in both the AGO1 input and IP fractions upon alignment to the expanded genome sequence (Figs 8H and S11C, right panel), and these sequences represented the majority of newly mapping reads (S11D Fig). ChNRV1-derived small RNAs were significantly more abundant in the 6H3F-AGO1 IP than the input, where we observed 186,362 more reads (Fig 8I) (95% confidence interval: 158,030 to 214,693). Analysis of 6H3F-AGO1-bound small RNAs by strand revealed an average ratio of 1.3 sense to antisense (Fig 8J), indicating that viral small RNAs loaded into AGO1 originated from both strands without a strong bias. A size distribution analysis determined that 21 nt was the predominant size in the 6H3F-AGO1 IPs (Fig 8K), and a 5’ nucleotide preference for 6H3F-AGO1-bound small RNAs was clear as nearly 99% of viral small RNAs loaded into AGO1 had a 5’U (Fig 8L). The strong 5’ nucleotide preference of AGO1 further demonstrates that the sequences observed in the Δ*dcl1* and Δ*dcl1*Δ*dcl2* datasets are not likely to be functional as these small RNAs are not the preferred substrate to be loaded into AGO1 for targeting the viral genome. 6H3F-AGO1-bound viral small RNAs originated from both strands and were distributed across the ChNRV1 genome (Fig 8M). Regions with the greatest signal in the 6H3F-AGO1 IP corresponded to the hotspots observed in the controls and mutant strains proficient at silencing ChNRV1 (see Fig 8G), suggesting that small RNAs from these loci were specifically being loaded into AGO1.

Here we used RNA-seq and small RNA datasets from high-throughput sequencing to characterize the antiviral role of RNA silencing machinery in *C*. *higginsianum*. Specifically, AGO1 and DCL1 were identified as crucial components as loss of either resulted in an increased accumulation of viral mRNA and genomic RNAs. DICER-like genes have been implicated in the antiviral response of other fungi. Characterization of the two DCL genes of *C*. *parasitica* revealed that the *C*. *higginsianum dcl1* homolog, *dcl2*, had a diminished response to infection by either the dsRNA reovirus MyRV1-Cp9B21 or the p29 suppressor mutant of the ssRNA hypovirus CHV1-EP713 [14]. Additionally, increased levels of viral RNAs were observed in the Δ*dcl2* and Δ*dcl1*Δ*dcl2* double mutant of *C*. *parasitica* [14] and viral small RNAs were no longer detected by Northern blot in the *C*. *parasitica dcl2* mutant [90]. Here we show direct evidence that AGO1 binds ChNRV1 small RNAs and the properties of AGO1-bound viral small RNAs, ~21-nt, 5’U preference, are consistent with previous reports for mycoviruses [90–92]. Argonaute-like proteins have also been identified as important for antiviral responses in fungi. Viral small RNAs from *Aspergillus* virus 341-infected *A*. *nidulans* were only detected in an *rdsA* mutant background, the *C*. *higginsianum* AGO1 homolog [91], indicating that the loss of this gene lead to an increase in levels of viral small RNAs. Further, the *C*. *parasitica* ortholog of *C*. *higginsianum* AGO1, AGL2, was also shown to be required for induction of an antiviral defense in response to infection by Δp29-CHV1-EP713 hypovirus [25]. The putative role(s) of RdRP proteins during *C*. *higginsianum* antiviral response remain to be determined; recent work with single and multiple mutants of *rdr* in *C*. *parasitica* indicated no contribution by RdRP genes to the antiviral response [26], and work in *A*. *nidulans* demonstrated that an RdRP was not required for silencing of transgene elements [93].

### Conidiation levels are restored in ChNRV1-free Δ*dcl1* strains

**Fig 9:**
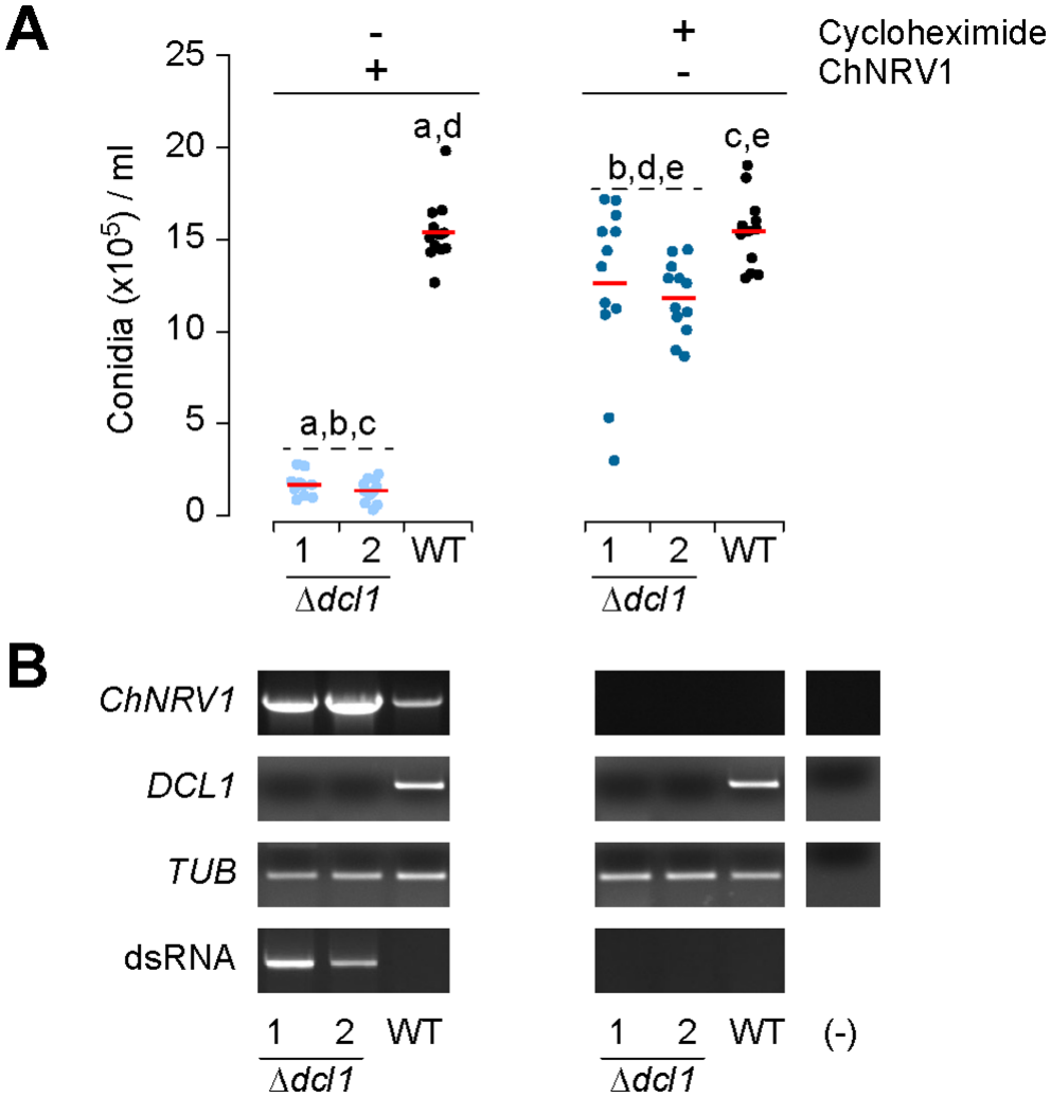
Conidiation in *C*. *higginsianum* Δ*dcl1* and wild-type strains with and without ChNRV1. (**A**) Conidia production in the Control and Δ*dcl1* mutant strains before cycloheximide treatment (–Cycloheximide/+ChNRV1) and after cycloheximide treatment (+Cycloheximide/–ChNRV1). Conidia were collected after 7 days of growth in Mathur’s medium and counted with a hemocytomer. Mean conidia counts are indicated by the red dash; significantly different pairwise comparisons are indicated by shared lowercase letters (p < 0.05). (**B**) RT-PCR analysis of *C*. *higginsianum* total RNA for the presence of ChNRV1, *DCL1*, and *tubulin* (control). The presence of dsRNA was determined by gel electrophoresis of total RNA (lower panel).

After determining that DCL1 and AGO1 were involved in virus regulation, we hypothesized that the defects in conidiation observed in these strains might be due to the activity of ChNRV1 rather than endogenous regulatory activity of RNA silencing. Therefore, we used a cyclohexamide treatment to obtain a *C*. *higginsianum* wild-type strain IMI 349063A cured of ChNRV1. *DCL1* deletion mutants were generated by targeted gene replacement using the *C*. *higginsianum* wild-type cured strain as the recipient background. Gene disruptions were confirmed by RT-PCR analysis of *DCL1*, identifying two independent deletion mutants for conidia quantification (Fig 9B). Virus-free Δ*dcl1* strains produced an average of 10.7x10^5^ conidia/ml more than ChNRV1-infected Δ*dcl1* (95% confidence interval: 9.6x10^5^–11.9x10^5^ conidia/ml), demonstrating an 8.8X increase in conidia production (Fig 9A). Further, conidiation levels in the Δ*dcl1* strain were restored to nearly 80% of the levels observed in the cured wild-type strain. These results indicate that virus proliferation in the Δ*dcl1* strain is the major contributing factor to the severe conidiation defect observed in the ChNRV1-infected Δ*dcl1* strains. Further work to generate and characterize of virus-free strains will be used to elucidate the contribution of RNA silencing during conidia production, particularly as work with *T*. *atroviride* strains lacking the ChDCL1 ortholog demonstrated severe deficiencies in conidia production [13]. As well, the virus-free strain can be used as a system to query the effects of viral RNA versus viral proteins in an RNA-silencing deficient strain to better understand the relative contribution of each element to the conidiation phenotype observed.

### Conclusions

Here we describe the antiviral role of *Colletotrichum higginsianum* silencing machinery against ChNRV1, a novel dsRNA virus present in the wild-type strain (IMI 349063A). When the fungal silencing machinery is functional, ChNRV1 is maintained at low levels. However, loss of either DCL1 or AGO1 leads to de-repression of ChNRV1, which consequently accumulates at very high levels. Increase in viral accumulation is responsible for the severe conidiation defect observed in Δ*dcl1*, Δ*dcl1*Δ*dcl2* strains, as virus-free Δ*dcl1* strains show strong recovery of the phenotype. The question remains why and how is ChNRV1 able to avoid the full impact of the antiviral defense. Possibly ChNRV1 confers some advantage to *C*. *higginsianum* in a yet to be studied environment, or it may act as vector a contributing to fungal genomic plasticity, enhancing the emergence of new virulence traits through evolution.

## Materials and Methods

### Fungal strains, mycelial growth and conidiation

The *Colletotrichum higginsianum* (IMI 349063A) wild-type strain used in this study corresponds to the sequenced strain isolated from *Brassica rapa* (Trinidad Tobago), kindly provided by Dr. Richard O’Connell [35]. The fungal strains deficient in the RNA silencing machinery *(*Δ*ago1*, Δ*ago2*, Δ*dcl1*, Δ*dcl2*, Δ*dcl1*Δ*dcl2 (*Δ*dcl1/2)*, Δ*rdr1*, Δ*rdr2*, and Δ*rdr3)* were obtained in this study by gene replacement. The fungal strains expressing the 6His-3FLAG-tagged AGO1 and AGO2 proteins were generated in this study by transformation of the previously generated Δ*ago1* and Δ*ago2* strains, respectively. Specific details related to the generation and confirmation of knock-out and tagged strains can be found in S2 Text. Growth tests were performed by culturing the fungus in Potato Dextrose Agar (PDA), Czapek dox agar (CDA), or Mathur’s Agar (Conidiation media) [94] at 25°C in dark conditions. Growth tests under nutrient limitations were performed using CDA depleted in carbon (CDA-C) or nitrogen (CDA-N) at 25°C in dark conditions. For growth tests under osmotic stress conditions, fungal strains were grown for 10 days in Mathur’s agar supplemented with 0.4 M NaCl, 0.2 M LiCl, 0.5 M Sorbitol, or 0.6 M Sucrose. Oxidative stress responses were analysed in Mathur’s media supplemented with 2 mM H_2_O_2_. Fungal conidiation was induced by growing the fungus on Mathur’s agar for 7 days at 25°C and dark conditions. Conidia were collected from fungal mycelium by adding sterile water to the surface of the mycelium. After filtration and microscopic observation, conidia were counted with a Neubauer counting chamber.

### Identification of the core components of the silencing machinery in Ascomycetes

The search for putative homologs of the RDR, DCL and AGO proteins in Ascomycete fungi analyzed in this work was done with BLASTP, using *N*. *crassa* protein domain sequences as input. Identification of homologs was as follows: RDR proteins were required to have an RNA-dependent Polymerase Domain, DCL proteins were required to have two RNAseIII-domains, and AGO proteins were required to have a PAZ and a PIWI domain. *C*. *higginsianum* gene names were assigned based to the silencing pathway to which they belong: AGO1, DCL1 and RDR3 for the Quelling pathway; AGO2, DCL2 and RDR1 for the MSUD pathway; and RDR2 for the Unknown pathway.

### Sequence and phylogenetic analysis

The deduced amino acid sequence for each RNA silencing protein was aligned with MAFFT [95]. Aligned sequences were imported into Molecular Evolutionary Genetics Analysis (MEGA v6.06) [96], and trimmed to exclude positions with gaps. The best model of protein evolution was determined using Prottest [97], based on the Akaike Information Criterion (AIC). The LG+G+F model was determined by Prottest to be the best-fit for both fungal RNA silencing protein and the viral RDR alignments. A maximum likelihood (ML) phylogenetic tree was constructed with RAXML [98] using the appropriate model of amino acid substitution. The tree was drawn using Dendroscope v3.2.10 [99].

### Light microscopy

For conidia enumeration, a 10 μl drop of conidia suspension was placed in a Neubauer chamber and covered with a cover slip. Three biological replicates from four individual transformants were counted for each genotype. Three different mm^2^ were analyzed from each biological replicate. For conidia measurements, pictures were taken using a Planapo x20 water immersion objective with a Confocal Laser Scanning microscope Leica TCS SP8, and image analysis was done with open-source ImageJ/Fiji v1.48 software (http://fiji.sc/Fiji). At least 200 conidia were measured for each genotype.

### Purification and observation of virus particles

Viral particles were partially isolated from the Δ*dcl1*, Δ*dcl1*Δ*dcl2* and Δ*ago1* strains following the methods described by Dunn and colleagues [69] with minor modifications. Fungal mycelia were grown for one month with constant agitation at (150 rpm, 25°C, dark). Approximately 70 mg of mycelia were homogenized with 6 ml of Tris buffer (0.1 M Tris-HCl, 0.15 M NaCl, 5 mM EDTA (pH 7.8). Hyphal debris was removed by centrifugation (10,000 rcf for 20 min at 4°C). The supernatant was collected and filtered through four layers of Miracloth (Calbiochem). To concentrate the virus particles, 0.15 M NaCl and 6% PEG6000 was added to the solution, incubated 1 h on ice, and precipitated by low-speed centrifugation (3,600 rcf for 20 min at 4°C). The supernatant was removed and the pellet was resuspended in 100 μl 0.1 M Tris-buffer (pH 7.8). Samples were analyzed immediately. The coat protein from viral particles was observed by running a 5 μl aliquot of resuspended viral particles on a NUPAGE-Novex 4–12% Bis-Tris protein gel (Invitrogen) and stained with Commassie blue G-250. dsRNA from viral particles was extracted from a 5 μl aliquot by phenol-chloroform-isoamyl alcohol, followed by ethanol precipitation and visualized on a 1% (wt/vol) agarose gel.

### Protein digestion and identification using liquid chromatography—tandem mass spectrometry (LC-MS/MS)

The gel bands were cut, de-stained and in-gel digested as previously described [100]. Five microliters of the digested peptides were injected to the LC-MS/MS system. The LC-MS/MS was carried out on a LTQ-Orbitrap Velos Pro (ThermoFisher Scientific, Waltham, MA) coupled with a U3000 RSLCnano HPLC (ThermoFisher Scientific, Waltham, MA). The protein digests were first loaded onto a C_18_ trap column (PepMap100, 300 μm ID × 5 mm, 5 μm particle size, 100 Å; ThermoFisher Scientific) at a flow rate of 5 μL/min for 4 min equilibrated with 2% acetonitrile, 0.1% formic acid. Peptide separation was carried out on a C_18_ column (Acclaim PepMap RSLC, 15 cm × 75 μm nanoViper, C18, 2 μm, 100 Å, ThermoFisher Scientific) at a flow rate of 0.3 μL/min and the following gradient: 0–3 min, 2% B isocratic; 3–41 min, 2–45% B; 45–47.8 min, 45–98% B. Mobile phase A, 0.1% formic acid; mobile phase B, 0.1% formic acid in 80:20 acetonitrile:water. The Orbitrap mass analyzer was operated in positive ionization mode using collision induced dissociation (CID) to fragment the HPLC separated peptides. The mass range for the MS survey scan done using the FTMS was 300 to 2000 m/z with resolution set to 60,000 @ 400 m/z and the automatic gain control (AGC) target set to 1,000,000 ions with a maximum fill time of 10 ms and 1 μscan. The 20 most intense signals in the survey scans were selected and fragmented in the ion trap using an isolation window of 1.5 m/z, an AGC target value of 10,000 ions, a maximum fill time of 100 ms, normalized collision energy of 35 and activation time of 30 ms. Dynamic exclusion was performed with a repeat count of 1, exclusion duration of 30 s, and a minimum MS ion count for triggering MS/MS set to 5,000 counts. All MS/MS samples were analyzed using Mascot (Matrix Science, London, UK; version 2.5.0). Mascot was set up to search the *Colletotrichum higginsianum* database from http://www.broadinstitute.org/annotation/genome/colletotrichum_group/ToolsIndex.html, (16,263 entries) including the sequence from CP, RdRP and CP-RdRP assuming the digestion enzyme trypsin. Mascot was searched with a fragment ion mass tolerance of 0.80 Da and a parent ion tolerance of 15 PPM. Deamidated of asparagine and glutamine, oxidation of methionine and carbamidomethyl of cysteine were specified in Mascot as variable modifications. Scaffold (version Scaffold_4.3.4, Proteome Software Inc., Portland, OR) was used to validate MS/MS based peptide and protein identifications. Peptide identifications were accepted if they could be established at greater than 95.0% probability by the Peptide Prophet algorithm [101] with Scaffold delta-mass correction. Protein identifications were accepted if they could be established at greater than 99.0% probability and contained at least 2 identified peptides. Protein probabilities were assigned by the Protein Prophet algorithm [102]. Proteins that contained similar peptides and could not be differentiated based on MS/MS analysis alone were grouped to satisfy the principles of parsimony. Proteins sharing significant peptide evidence were grouped into clusters.

### Electron microscopy

Cells were packed in specimen carriers in 75 mM PIPES buffer (pH 6.8) containing 50 mM sucrose, and ultra-rapidly frozen in a Bal-Tec high-pressure freezer (HPM 010, Technotrade International). Frozen samples were substituted in 2% osmium tetroxide plus 0.1% uranyl acetate in acetone for 5 days at -85°C, slowly thawed to 20°C and embedded in Spurr’s resin. Thin sections were cut using a LeicaUCT ultramicrotome, stained in uranyl and lead salts, and observed using a LEO 912 AB energy filter TEM (Zeiss).

The viral particles were stained with 2% (wt/vol) uranyl-acetate solution (pH 7.4) and observed using a transmission electron microscope.

### Curing experiments

Cycloheximide treatment was used to remove ChNRV1 from the wild-type *C*. *higginsianum* strain. In order to increase the chances of obtaining single conidia free of virus, mycelial plugs were inoculated in Mathur’s Agar supplemented with Cycloheximide at 10 μg/ml (Crescent Chemical Company) and allowed to conidiate for 15 days at 25°C in dark conditions. Conidia were collected by adding sterile water, then counted with a Neubauer counting chamber. About 100 conidia were spread onto 1% agar plates containing 0.5 ug/ml cycloheximide, and allowed to germinate at 25°C. After 3–4 days the margin of the colonies were collected and transferred to fresh Mathur’s agar.

### DNA extraction and Southern blots

Total nucleic acid from *C*. *higginsianum* was extracted using previously described methods [103], but with a mixed alkyltri-methylammonium bromide solution (MATAB) [0.1 M Tris HCl, pH 8.0, 1.4 M NaCl, 20 mM EDTA, 2% MATAB, 1% PEG 6000, 0.5% sodium sulfite] as the extraction buffer. DNA was purified by chloroform-isoamyl alcohol, followed by RNase treatment, chloroform-isoamyl alcohol, and ethanol precipitation. Ten μg of DNA was digested to completion with the indicated restriction enzymes (S3, S4 and S5 Figs) (New England Biolabs), separated on a 1% (wt/vol) agarose gel, and desired fragments were visualized after hybridized with the corresponding digoxigenin (DIG)-dUTP-labelled probe. Oligonucleotides used for probe preparation are listed in S7 Table. Probes were labeled with digoxigenin (DIG)-dUTP using the Random Primed DNA Labeling Kit (Roche).

### dsRNA 3′RACE analyses

To determine the terminal sequence of the dsRNA ChNSV1 genome, a RNA ligase mediated RACE (RLM-RACE) protocol was followed [59]. or dsRNA enrichment, total RNA was extracted using Trizol reagent (Life Technologies), followed by precipitation with 2M LiCl to remove single stranded (ss) RNA. Supernatant was collected and dsRNAs were precipitated with 4 M LiCl. dsRNAs were fractionated by 1% (wt/vol) agarose gel electrophoresis, gel-purified (PureLink Quick Gel Extraction Kit—Life Technologies) and denatured at 95°C for 5 min. The miRNA Cloning Linker 1 (IDT) (5'App/CTGTAGGCACCATCAAT/3'ddC/) was ligated to the 3’end of each strand of the denatured RNA using AIR Ligase (Bioscientific) in the presence of RNAseOUT (Invitrogen). The ligated products were used as templates for cDNA synthesis using Superscript III Reverse Transcriptase (Invitrogen) following the manufacturer’s instructions but with a denaturation step of 98°C for 10 min. The resulting cDNAs were amplified with the primers *ORF1-reverse/P7-modban* and *ORF2-forward/P7-Modban* to amplify the 3’ end from each strand. A total of 12 PCR reaction products for both strands were cloned into pCR-BluntII (Invitrogen) for sequence analysis.

### Semi-quantitative (RT-PCR), quantitative real-time PCR (RT-qPCR) and northern blot analysis

Total RNA from *C*. *higginsianum* tissue was extracted using TRIzol reagent (Life Technologies). One microgram was treated with TURBO DNase I DNA-free (Ambion) to remove genomic DNA contamination. cDNA synthesis was performed using the Superscript III system (Life Technologies) following the manufacturer’s instructions. RT-PCR analysis was performed using 10% of the first-strand reaction and 40 cycles of amplification to guarantee the detection of the amplicon. RT-qPCR analysis used 3% of the first strand reaction as previously described [104,105]. Oligonucleotides used for RT-PCR and RT-qPCR analysis are listed in S7 Table.

For Northern blot analysis of viral siRNAs, 2.5 μg of Total RNA was subjected to 17% polyacrylamide (containing 7M Urea) electrophoresis, and transferred to a positively charged, nylon membrane (Roche) using a semi-dry electroblotting apparatus (Biostep). Blots were pre-hybridized in PerfectHyb Hybridization buffer (Sigma), followed by hybridization with a DIG-labeled DNA probe corresponding to the CP (ORF1) sequence. The DIG-labeled DNA probe was created by amplifying the coat protein sequence using oligonucleotides listed in S7 Table, cloning the resulting amplicon into pCR4 (Life Technologies), digestion with EcoRI (NEB) and gel purification (Life Technologies), then labeled following the manufacturer’s instructions for the DIG DNA Labeling Kit (Roche).

### RNA immunoprecipitation for high-throughput small RNA sequencing

One gram of mycelia tissue from wild-type, Δ*ago1/*6H3F-AGO1 and Δ*ago2/*6H3F-AGO2 *C*. *higginsianum* strains was used as the starting material for AGO-immunoprecipitation described in Carbonell et al. [104], but with the following modifications. Clarified lysates were incubated with 4 μg/ml of Monoclonal ANTI-FLAG M2 Antibody (Sigma) for 3 h at 4°C, followed by 100 μl of Protein-G agarose (Roche) per milliliter for 30 min at 4°C. RNAs recovered from input (before immunoprecipitation) and IP fraction were used for preparation of small RNA libraries as described below.

### Preparation of small RNA libraries

Small RNA libraries were prepared following the detailed protocol previously described [105,106]. Specific modifications include using 40 μg of total RNA of *C*. *higginsianum* 4-days old mycelia and a 2 hr incubation for the 3’ ligation reaction.

### Small RNA sequencing analysis

Libraries were sequenced using the Illumina HiSeq 2000, v3 chemistry at the Genome Technology Access Center (GTAC) (Washington University, Saint Louis, Missouri). Scripts used for initial processing of the small RNA data are available on GitHub (https://github.com/carringtonlab/srtools). LibParse.pl was used to remove the 3’ adaptor and de-multiplex the sequences in the raw (FASTQ) file. For all lanes of data, the following common settings were used: -t fastq -r 50 -m 18 -e CTGTAG–E (comma-separated list of the 10–12 barcodes used to index the individual samples during library preparation) -l (log_file.txt) -a (failed_parsing_file.txt). Reads less than 18 nt in length and reads containing one or more N nucleotides were discarded. Remaining small RNA reads were aligned to the *C*. *higginsianum* genome (version 1 assembly; *Colletotrichum* Sequencing Project, Broad Institute of Harvard and MIT (http://www.broadinstitute.org/)) using the Bowtie algorithm [53] (version 0.12.8) allowing only perfect matches and reporting all mappings for reads that map multiple times to the genome. Reads that mapped and reads that did not map to the genome were quantified using the Bowtie output files and get_parsed_mapped_unmapped_stats.pl.

RepeatMasker (version open-3.3.0 [107]) and IRF (Inverted Repeats Finder, version 3.05) [108] were used to identify repetitive elements within the *C*. *higginsianum* genome. These features were added to the features table in a custom SQLite3 database along with the *C*. *higginsianum* version 1 gene annotation available from the *Colletotrichum* Sequencing Project (Broad Institute of Harvard and MIT, http://www.broadinstitute.org/).

### Preparation of strand-specific RNAseq libraries

Strand-specific RNAseq libraries were produced using the same RNA samples used for preparation of small RNA libraries. Ten μg of total RNA was treated with TURBO DNase I DNA-free (Ambion). For depletion of ribosomal RNAs, 1.5μg of DNase-treated RNA was treated with the Yeast RiboZero Magnetic Gold Kit (Epicentre) according to manufacturer’s instructions. cDNA synthesis was performed using 70 ng of RiboZero-treated RNA as previously described [104,109] with the following modifications. RNAs were fragmented at 94°C for 7 min, and 12 cycles were used in the linear PCR reaction. DNA adaptors 1 and 2 were annealed to generate the Y-shape adaptors, and oligonucleotides PE-Primer-F and PE-Primer-R (series N701-N712) were used for linear PCR and multiplexing of transcript libraries (S7 Table). DNA amplicons were analyzed with a Bioanalyzer (DNA HS kit, Agilent) to ensure a library size of ~250 bp, quantified using the Qubit HS Assay Kit (Invitrogen) and sequenced on a HiSeq 2000 sequencer (Illumina) at GTAC (Washington University).

### RNA-seq analysis

FASTQ files of RNA-seq data were de-multiplexed using the script parseFastq.pl and then Bowtie2 (version 2.1.0) [52] was used to align the reads to the reference genome. The indexed bam output from bowtie2 and the script get_RNAseq_mapped_unmapped_stats.pl were used to identify the total number of reads that mapped and that did not map for each genotype.

### De novo transcriptome assembly

The program Trinity (version Trinityrnaseq_r20131110, http://trinityrnaseq.github.io/) was used for *de novo* assembly of RNA-seq reads. The FASTQ files for the four Δ*dcl1* replicates were combined into one FASTQ file and used as the input for Trinity. Separately the unmapped reads from the bowtie2 alignment for Δ*dcl1* (replicate 1) were converted into FASTQ format (bam_2_fastq.noHit.pl), and aligned to the Trinity contigs using the utility program alignReads.pl provided with the r20131110 version of Trinity. As the *C*. *higginsianum* genome does not contain the mitochondrial genome, a custom BLAST database was created from the mitochondrial sequences of *C*. *graminicola* (available at http://www.broadinstitute.org/) and *C*. *lindemuthianum* (http://www.ncbi.nlm.nih.gov/nuccore/KF953885.1). Trinity contigs were aligned to this mitochondrial database using BLASTN. A non-redundant set of 26 contigs was concatenated together to create the *C*. *higginsianum* mitochondrial RNA feature. To identify high-coverage contigs, the samtools idxstats command was used on the output from alignReads.pl: coordSorted.bam. Putative gene identities were determined using BLASTX and the nr database. In total, three additional features were added to the features table of the SQLite3 database and the *C*. *higginsianum* genome sequence: (1) viral sequence (2,923 bp), (2) a large subunit rRNA sequence (4,077 bp), and (3) mitochondrial RNA sequence (30,330 bp).

### Small RNA and RNA-seq analyses using a modified *C*. *higginsianum* genome sequence

A new reference genome sequence was created with the addition of the three contigs to the *C*. *higginsianum* genome sequence. RNA-seq reads were aligned to the modified genome using Bowtie2 (version 2.1.0) [52]. Mapped and unmapped reads were determined using get_RNAseq_mapped_unmapped_stats.pl. To determine the distribution of the newly mapped reads, the script reads_mapped_2_chromNum.RNAseq.pl was used. The distribution of RNA-seq by strand mapped to the virus sequence was determined using readsPerFeature.strand.byRep.RNAseq.pl.

Small RNAs were aligned to the new reference sequence using bowtie, with perfect matches only allowed. An SQLite3 database was created from the parsed and aligned reads using the scripts PopulateDB.pl and Merge.pl (https://github.com/carringtonlab/srtools). Mapped and unmapped read counts were determined using the script get_parsed_mapped_unmapped_stats.pl. The distribution of newly mapped reads was determined using reads_mapped_2_chromNum.FinalGenome.pl. To determine the distribution of mapped reads by genomic feature category sizeNT_byCatID.FinalGenome.pl was used. This script also created the 5’ nucleotide profile data. Small RNAs mapped by strand to the virus were identified using readsPerFeature.strand.byRep.smRNA.pl. Size distribution of viral small RNAs by strand was determined using the script sizeNT_byCatID.byRep.byStrand.FinalGenome.pl.

### Statistical analysis and data visualization of viral small RNA and RNA-seq

Small RNA and RNA-seq reads mapped to the virus genome were scaled using the total number of reads in each respective library. Additionally, the scaled abundance of reads mapped to the sense strand and antisense strand were also determined. Data were analyzed in R (version 3.0.3) with a one-way ANOVA with Tukey post hoc analysis. Visualization of scaled and ratio data were also performed in R.

For plotting small RNAs along the virus genome, first the scaled read count per nucleotide, by strand, was determined using hitsPerNT.smRNA.strand.pl. The per-nucleotide values were then each divided by the average number of scaled RNA-seq reads mapped to that strand of the virus. A density map of reads along the sense and antisense strands of the virus was created in R. The hits per nucleotide per strand from the small RNA data of the AGO1 IP similarly plotted, but was not normalized by RNA-seq data.

### Structure prediction

3D structure prediction of ORF1 of the viral genome was performed with I-Tasser (version 3) on-line server [65]. The known structure of the *Saccharomyces cerevisiae* L-A virus major coat protein (PDBID: 1m1c) [64] was used as a template. The resulting protein structures were visualized using Chimera (http://www.cgl.ucsf.edu/chimera) [110].

### Accession numbers

Accession numbers for the fungal protein sequences analyzed in this study are listed in S1 Table. Accession numbers for *Colletotrichum destructivum/higginsianum* species are listed in S4 Table. Accession numbers for viral proteins are listed in S6 Table. The complete nucleotide sequence of the dsRNA virus ChNRV1 was deposited at NCBI in GenBank under the accession number KM923925. De-multiplexed fastq files of the small RNA, IP, and RNA-seq libraries, along with a gff3 file of counts and alignments to the viral genome, are available at the Gene Expression Omnibus (GEO, http://www.ncbi.nlm.nih.gov/geo/) under the SuperSeries accession GSE62708; subseries GSE62705, GSE62706, and GSE62707.

## Supporting Information

S1 Text Sequences of rRNA and mtRNA contigs identified via de novo assembly of Δ*dcl1* RNA-seq reads. (DOCX)

S2 Text Supplementary Methods. (DOCX)

S1 Movie Animation of the ChNRV1 ORF1, putative coat protein, aligned to *Saccharomyces cerevisiae* virus L-A (ScV-L-A) coat protein. ChNRV1-CP is in white and ScV-L-A-CP in cyan. N-terminal and C-terminal ends of ChNRV1 are denoted in purple and orange respectively.
(MOV)

S1 Fig Dicer and Argonaute domains in eukaryotes. (**A**) Domain arrangement of Dicer (DCL) proteins from several species. Accession numbers for DCLs of *Colletotrichum higginsianum* (ChDCL1: CH063_06582, ChDCL2: CH063_02619), *Neurospora crassa* (NcDCL2: NCU06766), *Schizosaccharomyces pombe* (SpDCR: NP_588215), *Toxoplasma gondii* (TgDCR: TGME49_267030), *Chlamydomonas reinhardtii* (CrDCR: XP_001692436), *Homo sapiens* (Hs-DCR: Q9UPY3), and *Arabidopsis thaliana* (AtDCL1: Q9SP32). (**B**) MAFFT alignment of amino acid residues involved in the 5’ phosphate binding (MID domain) and slicer activity (PIWI domain) in the selected AGO proteins. Position shown to interact specifically with the 5’ phosphate (MID) and Mg^+^ coordinating residues (PIWI) are labeled with a red asterisk [111–114]. Accession numbers for AGOs of *Colletotrichum higginsianum* (ChAGO1: CH063_04066, ChAGO2: CH063_09722), *Neurospora crassa* (NcQDE2: NCU04730, NcSMS2: NCU09434), *Magnaporthe oryzae* (MG1: MGG_01294), *Arabidopsis thaliana* (AtAGO1: U91995, AtAGO2: Q9SHF3), *Drosophila melanogaster* (DmAGO1: CG6671) and *Homo sapiens* (HsAGO2: Q9UKV8).
(TIF)

S2 Fig Expression analysis of *RDR*, *DCL* and *AGO* genes in *C*. *higginsianum* in different stages of fungal development. Gene expression in (**A**) Conidia and (**B**) Germinated Conidia was analyzed. Silencing genes belonging to the Quelling pathway (left panel), MSUD pathway (middle panel) and Unknown pathway (right panel) are indicated. Values represent means +/- SE of three biological replicates normalized to *ACTIN* and *TUBULIN* genes as a relative value to *AGO1*, as determined by qRT-PCR.
(TIF)

S3 Fig Targeted gene disruption of the *C*. *higginsianum RDR1*, *RDR2*, and *RDR3 genes*. (**A-C**) Schematic diagram showing the target replacement strategy (TGR) for (**A**) *RDR1* (CH063_02767), (**B**) *RDR2* (CH063_05776) and (**C**) *RDR3* (CH063_08349). Hygromycin (*HPH*) resistance was used as a selectable marker. Colored arrows indicate primers used for amplification and generation of TGR constructs (S7 Table). For D-H, one gel was used for each gene and probe or primer set; non-contiguous lanes are separated by white space. (**D-F**) Integration analysis by Southern blot for (**D**) *RDR1*, (**E**) *RDR2* and (**F**) *RDR3*. One wild-type (WT) and four independent mutant strains were analyzed. (**D**) A single 7.02 kb band was observed in *Stu*I-digested genomic DNA of WT strains when using the 1.89 kb *RDR1* probe (within the deleted region of *RDR1*). A 7.05 kb unique band was detected in only the Δ*rdr1* mutant strains when using the 2 kb *HPH* probe; no band observed in the WT strain. (**E**) A single 4.90 kb band was observed in *Sal*I-digested genomic DNA of WT when using the 1.90 kb *RDR2* probe. A 5.38 kb unique band was detected in only the Δ*rdr2* mutant strains when using the 2 kb HPH probe. (**F**) A single 3.99 kb band was observed in *Pvu*II-digested genomic DNA of WT when using the 2.11 kb *RDR3* probe. A 5.00 kb unique band was detected in only the Δ*rdr3* mutant strains when using the 2 kb *HPH* probe. (**G-H**) Confirmation of gene knock-out by semi-quantitative RT-PCR analysis of (**G**) *RDR1*, *RDR2*, and *RDR3* and (**H**) *tubulin* in the corresponding mutant and WT strains. Black arrows in (A-C) denote primers located in exon junctions designed for specific amplification of the RNA transcripts (S7 Table).
(TIF)

S4 Fig Targeted gene disruption of the *C*. *higginsianum DCL1* and *DCL2* genes. (**A-C**) Schematic diagram showing the target replacement strategy (TGR) for (**A**) *DCL1* (CH063_06582), (**B**) *DCL2* (CH063_02619) and (**C**) both *DCL1* and *DCL2*. Hygromycin (*HPH*) resistance was used as a selectable marker for single mutants (**A-B**). Phleomycin (*PHLE*) resistance was used as a selectable marker for generation of double Δ*dcl12*Δ*dcl2* mutant in the single mutant Δ*dcl2* background (hph resistance) (**C**). Colored arrows indicate primers used for amplification and generation of TGR constructs (S7 Table). (**D-F**) Integration analysis by Southern blot for (**D**) *DCL1*, (**E**) *DCL2* and (**F**) *DCL1 in* Δ*dcl2* background. Four independent mutant and wild-type (WT) strains were analyzed. (**D**) A single 5.34 kb band was observed in *Bgl*II-digested genomic DNA of wild-type when using the 3kb *DCL1* probe (deleted region in Δ*dcl1* mutants). A 5.44 kb unique band was detected in all the Δ*dcl1* mutant strains when using the 2kb *HPH* probe. (**E**) Two bands were observed in the *Xho*I-digested genomic DNA of the wild-type strain as expected; this was due to the initially designed probe, from the deleted region of *DCL2*, hybridizing across the digestion site. A single 5.58 kb band was detected in all the Δ*dcl2* mutant strains when using the 2 kb *HPH* probe. (**F**) A single 5.34 kb was observed in *Bgl*II-digested genomic DNA of wild-type when using the 3 kb *DCL1* probe. A 6.41 kb unique band was detected in all the Δ*dcl1*Δ*dcl2* mutant strains when using the 2.93 kb probe *PHLE* probe. Disruption of the *DCL2* (CH063_02619) gene was re-confirmed using an improved probe that hybridized to a single, unique band in wild-type. (**G-I**) Confirmation of gene knock-out by expression analysis of *DCL1* (**G**), *DCL2* (**H**), and *DCL1* and *DCL2* (**I**) in the corresponding mutant backgrounds as determined by semi-quantitative RT-PCR. Black arrows in (A-C) denote primers located in exon junctions designed for specific amplification of the corresponding RNA transcripts (S7 Table).
(TIF)

S5 Fig Targeted gene disruption of the *C*. *higginsianum AGO1* and *AGO2* genes. (**A-B**) Schematic diagram showing the target replacement strategy (TGR) for (**A**) *AGO1* (CH063_04066) and (**B**) *AGO2* (CH063_09722). Hygromycin (*HPH*) resistance was used as a selectable marker. Colored arrows indicate primers used for amplification and generation of TGR constructs (S7 Table). (**C-D**) Integration analysis by Southern blot for (**D**) *AGO1* and (**E**) *AGO2*. Four independent mutants and one wild-type (WT) strain were analyzed. (**D**) A single 7.14 kb band was observed in *Sal*I-digested genomic DNA of wild-type when using the 2 kb *AGO1* probe (deleted region in Δ*ago1* mutants). A 7.02 kb unique band was detected in all the Δ*ago1* mutant strains when using the 2 kb *HPH* probe. (**E**) A single 4.94 kb band was observed in *Xho*I-digested genomic DNA of wild-type when using the 0.8 kb *AGO2* probe. A larger, unique band was detected in all the Δ*ago2* mutant strains when using the 2 kb *HPH* probe. (**E-F**) Confirmation of gene knock-out by expression analysis of (**G**) *AGO1* and (**F**) *AGO2* in the corresponding mutant backgrounds as determined by semi-quantitative RT-PCR. Black arrows in (A-B) denote primers located in exon junctions designed for specific amplification of the corresponding RNA transcripts (S7 Table).
(TIF)

S6 Fig Disruption of RNA silencing genes in *C*. *higginsianum* does not negatively affect vegetative growth. Vegetative growth in the *RDR*s (**A**), *DCL*s (**B**) and *AGO*s (**C**) mutant strains on PDA and CDA media. Representative images of colony morphology after six days of growth (left panel) and measurements of radial growth from 2 to 5 days (right panel) (mean +/- SE). Scale bar = 1 cm. PDA, Potato Dextrose Agar. CDA, Czapek Dox Agar.
(TIF)

S7 Fig Disruption of *DCL1*, *DCL2*, or *DCL1DCL2* in *C*. *higginsianum* does not negatively affect vegetative growth under stress-related conditions. Colony morphology of Δ*dcl1*, Δ*dcl2*, Δ*dcl1*Δ*dcl2* and wild-type (WT) under selected stress -related conditions. Cultures were grown at 25°C and dark conditions. Scale bar = 1 cm. (**A**) Strains were grown for four days on Mathur’s media alone (–) or supplemented with a stress component: 2 mM H_2_O_2_, 5 mM Methyl Viologen (MV), 200 mg/ml Calcofluor white (CFW). (**B**) Strains were grown for three days on Mathur’s media alone (–) or supplemented with an osmotic stress component: 0.4 M NaCl, 0.2 M LiCl, 0.5 M Sorbitol and 0.6 M Sucrose. (**C**) Strains were grown for seven days on CDA media without Carbon (CDA-C) or without Nitrogen (CDA-N).
(TIF)

S8 Fig Generation and molecular analysis of *C*. *higginsianum* strains expressing tagged versions of AGO proteins. (**A**) Schematic diagram of the constructs. A 6His-3FLAG (6H3F) epitope was cloned in frame with *AGO1* or *AGO2* and expressed under the control of its own promoter (prom). *C higginsianum* Δ*ago1* and Δ*ago2* mutant strains expressing the Hygromycin resistance (HygR) were transformed with the corresponding tagged AGO1 and AGO2 constructs harboring the Phleomycin resistance (PhleR). UTR, Untranslated region. (**B**) Analysis of integration by Southern blot using the *Phleomycin* probe. Only those strains showing a single hybridization pattern with *Hind*III-digested genomic DNA, indicative of a single copy integration event, were selected for further analysis (indicated with an asterisks). Numbers above the blot refer to the ID of the three AGO1 and two AGO2 independent transformants selected. (**C**) Colony morphology after 3 days of growth for the selected *C*. *higginsianum* Δ*ago1*/6H3F-AGO1 (three independent transformants), Δ*ago2*/6H3F-AGO2 (two independent transformants), and control (wild-type) strains. Scale bar = 1 cm (**D**) Conidia production in controls strains (wild-type and empty vector), Δ*ago1* mutant strains, and Δ*ago1*/6H3F-AGO1 strains. Conidia were collected after 7 days of growth in Mathur’s medium and counted with a hemocytomer. Significantly different pairwise comparisons are indicated by shared lowercase letters (p < 0.05). (**E**) Immunoblots of protein extracts from Δ*ago1*/6H3F-AGO1 (upper panel) and Δ*ago2*/6H3F-AGO2 (lower panel) with a wild-type (WT) control. (**F**) Immunoprecipitation of *C*. *higginsianum* tagged-AGOs. Immunoblots of protein extracts from input (in) and immunoprecipitated (IP) samples from Δ*ago1*/6H3F-AGO1 (upper panel) and Δ*ago2*/6H3F-AGO2 (lower panel) with a wild-type (WT) control. 6H3F-AGO2 was not detected in the IP samples, even with longer exposures (data not shown).
(TIF)

S9 Fig Comparison of capsid proteins of ChNRV1 and the selected members from the *Totiviridae*, *Partitiviridae* and *Amalgaviridae*. Capsid secondary structures α-helix and ß-sheets are shown in pink and orange, respectively. *Colletotrichum higginsianum* Non-segmented dsRNA virus 1 (ChNRV1), *Saccharomyces cerevisiae* virus L-A (ScV-L-A), *Penicillium stoloniferum* virus S (PsV-S), *Southern tomato virus* (STV). aa, amino acids.
(TIF)

S10 Fig ChNRV1 proteins identified using liquid chromatography—tandem mass spectrometry (LC-MS/MS). (**A-B**) Protein sequence coverage identified for ChNRV1 in samples p110, p36, p34, and p26 in both replicates (R1 and R2) in the Δ*ago1* and wild-type (WT) samples. Highlighted in blue are the peptides matching to the capsid protein and in orange to RdRP protein in the Δ*ago1* mutant (**A**) and wild-type (WT) (**B**) strains. (**C**) Extracted ion chromatograms (XIC) of the selected N-terminal peptide (detected at 810.78 m/z, z = 3) from p36, p34, and p26 samples from the Δ*ago1* mutant strain. Retention time and integrated peak area are indicated next to the peak.
(TIF)

S11 Fig Mapping statistics versus original and new reference genome sequence. (**A**) Percent of total RNA-seq reads by category: mapped to original reference genome, mapped to new sequences, or remain unmapped. (**B**) Breakdown of the source of newly mapping RNA-seq reads. (**C**) Percent of total small RNA reads by category: mapped to original reference genome, mapped to new sequences, or remain unmapped. (**D**) Breakdown of the source of the newly mapping small RNA reads.
(TIF)

S12 Fig qRT-PCR and northern blot of ChNRV1 RNAs. **(A)** qRT-PCR analysis of *ChNRV1* levels in mutant and wild-type backgrounds using primers for the RDRP sequence. Mean fold change (2^-ΔΔCt) relative to *ChNRV1* levels in wild-type (+/- standard deviation) is plotted. Four biological replicates were averaged and *ACTIN* and *TUBULIN* genes were used for normalization. **(B)** Small RNAs in total RNA were separated using a 17% polyacrylamide/urea gel, followed by transfer to a membrane and probed with a DIG-labeled probe generated from the coat protein sequence. Top panel is the ethidium bromide stain gel demonstrating equal loading and intactness of RNAs greater than 30 nt for each replicate. Lower panel is the blot of lower region of the gel. A 21 nt known sequence from the ChNRV1 coat protein sequence was included as a control for size and probe-specificity. A diffuse signal is visible in the Δ*dcl1* replicates, while no signal is apparent in either Δ*dcl1*Δ*dcl2* or the WT sample. The Δ*ago1* lanes show a band at 22 nt in each replicate.
(TIF)

S13 Fig Read size distribution by strand and 5’ nucleotide of viral small RNAs. (**A**) Read size distribution as a percentage of total viral small RNAs by strand for Δ*rdr1*, Δ*rdr2*, Δ*rdr3*, Δ*ago2*, and Δ*dcl2*. The distribution of the control replicates, found also in Fig 8D, is included in each panel for reference. (**B**) 5’ nucleotide distribution for viral small RNAs from Δ*rdr1*, Δ*rdr2*, Δ*rdr3*, Δ*ago2*, and Δ*dcl2*. 5’ nucleotide distribution for control replicates, found also in Fig 8E, is included for reference.
(TIF)

S14 Fig Determination for F2dU sensitivity in *C*. *higginsianum*. **(A)** Growth of *C*. *higginsianum* conidia in the presence of 5-fluoro-2’-deoxyuridine (F2dU) at concentrations ranging from 0.005–50 mM. Only the highest concentration of F2dU (50 μM) had a visible, negative, effect on *C*. *higginsianum* growth. (**B**) Growth of *C*. *higginsianum* conidia un-transformed (WT) and transformed with a vector containing the *Hvtk* and *Hyg* genes (*pGKO2-Hyg*) in the presence of Hygromycin (150 μg/mL) and F2dU at a range of concentration not toxic for *C*. *higginsianum* conidia (0.005–5 μM) as determined in (A). An optimal concentration of 150 μg/mL Hygromycin and 0.5 μM of F2dU was determined to guarantee an optimal growth of homologous recombinants (no presence *Hvtk* gene) and death for ectopic transformant (presence *Hvtk* gene). *Hyg*, Hygromycin. *Hvtk*, herpes Virus thymidine kinase.
(TIF)

S1 Table Accession numbers used for generating the phylogenetic tree for the RDRs, DCLs, and AGOs in the Ascomycota clade. (DOCX)

S2 Table Summary of sequencing data from RNA-seq libraries. (DOCX)

S3 Table Summary of sequencing data from small RNA libraries. (DOCX)

S4 Table Accession numbers and geographical accessions for the *Colletotrichum destructivum/higginsianum* strains used in this study. (DOCX)

S5 Table Identification of ChNRV1 proteins in *C*. *higginsianum* Δ*ago1* and wild-type strains following 1-D SDS-PAGE and mass spectrometry. (DOCX)

S6 Table Accession numbers used for generating the phylogenetic tree for the RDR from selected mycoviruses. (DOCX)

S7 Table Primers used in this study. (DOCX)
