# Supplementary material for: Small RNA-Based Antiviral Defense in the Phytopathogenic Fungus Colletotrichum higginsianum
Source: PLoS Pathog. 2016 Jun 2;12(6):e1005640. doi: 10.1371/journal.ppat.1005640 (PMC4890784; doi:10.1371/journal.ppat.1005640)
Supplement: S1 Text — (DOCX) [file ppat.1005640.s001.docx]

**S1 Text. Sequences of rRNA and mtRNA contigs identified via *de novo* assembly of ∆*dcl1* RNA-seq reads.**

>rRNA_contig_4077bp

CCCCCCCCCCCCCCCCCCCCCCGGGGGGGGGGGGGGGGGAGACAACAAAGGCTTAATCTCAGCAGATCGTAACAACAAGGCTACTCTACTGCTTACAATACCCCGTTGTACATCTAAGTCGTATACAAATGATTTATCCCCACGCAAAATGACATTGCAATTCGCCAGCAAGCACCCAAGGCCTTTCCGCCAAGTGCACCGTTGCTAGCCTGCTATGGTTCAGCGACGCCACAAGGACGCCTTATTCGTATCCATCTATATTGTGTGGAGCAAAGAAATCACCGCGTTCTAGCATGGATTCTGACTTAGAGGCGTTCAGCCATAATCCAGCGGATGGTAGCTTCGCGGCAATGCCTGATCAGACAGCCGCAAAAACCAATTATCCGAATGAACTGTTCCTCTCGTACTAAGTTCAATTACTATTGCGGTAACATTCATCAGTAGGGTAAAACTAACCTGTCTCACGACGGTCTAAACCCAGCTCACGTTCCCTATTAGTGGGTGAACAATCCAACGCTTACCGAATTCTGCTTCGGTATGATAGGAAGAGCCGACATCGAAGAATCAAAAAGCAATGTCGCTATGCACGCTTGACTGCCACAAGCCAGTTATCCCTGTGGTAACTTTTCTGGCACCTCTAGCCTCAAATTCCGAGGGACTAAAGGATCGATAGGCCACACTTTCATGGTTTGTATTCACACTGAAAATCAAAATCAAGGGGGCTTTTACCCTTTTGTTCTACTGGAGATTTCTGTTCTCCATGAGCCCCCCTTAGGACATCTGCGTTATCGTTTAACAGATGTGCCGCCCCAGCCAAACTCCCCACCTGACAATGTCTTCAACCCGGATCAGCCCCGAATGGGACCTTGAATGCTAGAACGTGGAAAATGAATTCCAGCTCCGCTTCATTGAATAAGTAAAGAAACTATAAAGGTAGTGGTATTTCACTGGCGCCGAAGCTCCCACTTATTCTACACCCTCTATGTCTCTTCACAATGTCAAACTAGAGTCAAGCTCAACAGGGTCTTCTTTCCCCGCTGATTCTGCCAAGCCCGTTCCCTTGGCTGTGGTTTCGCTAGATAGTAGATAGGGACAGTGGGAATCTCGTTAATCCATTCATGCGCGTCACTAATTAGATGACGAGGCATTTGGCTACCTTAAGAGAGTCATAGTTACTCCCGCCGTTTACCCGCGCTTGGTTGAATTTCTTCACTTTGACATTCAGAGCACTGGGCAGAAATCACATTGCGTCAACATCACTTTCTGACCATCGCAATGCTATGTTTTAATTAGACAGTCAGATTCCCCTTGTCCGTACCAGTTCTAAGTTGATCGTTAATTGTAGCAAGCGACGGTCTACAAGAGACCTACCAAGGCCGTCTACAACAAGGCACGCAAGTAGTCCGCCTAGCAGAGCAAGCCCCACCAAGCAGTCCACAAGCACGCCCGCTGCGTCTGACCAAGGCCCTCACTACCCGACCCTTAGAGCCAATCCTTATCCCGAAGTTACGGATCTATTTTGCCGACTTCCCTTATCTACATTATTCTATCAACTAGAGGCTGTTCACCTTGGAGACCTGCTGCGGTTATCAGTACGACCTGGCATGAAAACTATTCCTTCCTGTGGATTTTCACGGGCCGTCACAAGCGCACCGCACGGGCCGTCACAAGCGCACCGCACGGGCCGTCACAAGCGCACCGCACGGGCCGTCACAAGCGCACCGCGTTAGACTCCTTGGTCCGTGTTTCAAGACGGGCGGCATATAACCATTATGCCAGCATCCTTGACTTACGTCGCAGTCCTCAGTCCCAGCTGGCAGTATTCCCACAGGCTATAATACTTACCGAGGCAAGCTACATTCCTATGGATTTATCCTGCCACCAAAACTGATGCTGGCCCAGTGAAATGCGAGATTCCCCTACCCACAAGGAGCAGAGGGCACAAAACACCATGTCTGATCAAATGCCCTTCCCTTTCAACAATTTCACGTACTTTTTCACTCTCTTTTCAAAGTTCTTTTCATCTTCCCTCTTCCAGCCATAAGACCCCATCTCCGGATAAACCAATTCCGGGGTGATAAGCTGTTAAGAAGAAAAGATAACTCCTCCCAGGGCTCGCGCCGACGTCTCCACATTCAGTTACGTTACCGTGAAGAATCCATATCCAGGTTCCGGAATCTTAACCGGATTCCCTTTCGATGGTGGCCTGCATAAAATCAGGCCTTTGAAACGGAGCTTCCCCATCTCTTAGGATCGACTAACCCACGTCCAACTGCTGTTGACGTGGAACCTTTCCCCACTTCAGTCTTCAAAGTTCTCATTTGAATATTTGCTACTACCACCAAGATCTGCACTAGAGGCCGTTCGACCCGACCTTACGGTCTAGGCTTCGTCACTGACCTCCACGCCTGCCTACTCGTCAGGGCATCATATCAACCCTGACGGTAGAGTATAGGTAACACGCTTGAGCGCCATCCATTTTCAGGGCTAGTTCATTCGGCCGGTGAGTTGTTACACACTCCTTAGCGGATTCCGACTTCCATGGCCACCGTCCGGCTGTCTAGATGTTACCTCATAAAACTGATACGAGCTTCTGCTATCCTGAGGGAAACTTCGGCAGGAACCAGCTACTAGATGGTTCGATTAGTCTTTCGCCCCTATACCCAAATTCGACGATCGATTTGCACGTCAGAACCGCTACGAGCCTCCACCAGAGTTTCCTCTGGCTTCACCCTATTCAGGCATAGTTCACCATCTTTCGGGTCCCAACAGCTATGCTCTTACTCAAATCCATCCGAAGACATCAGGATCGGTCGATTGTGCACCTCTTGCGAGGCCCCAACCTACGTTCACTTTCATTACGCGTATGGGTTTTACACCCAAACACTCGCATAGACGTTAGACTCCTTGGTCCGTGTTACAAGACGGGCGCCGCAGGTTCACCTACGGAAACCTTGTTACGACTTTTAGTTCCTCTAAATGACCAAGTTTGTCCAAATTCTCCGCTCTGAGATGGAGTTGCCCCCTTCTCTAAGCAGATCCTGAGGCCTCACTAAGCCATTCAATCGGTACTAGCGACGGGCGGTGTGTACAAAGGGCAGGGACGTAATCAACGCAAGCTGATGACTTGCGCTTACTAGGAATTCCTCGTTGAAGAGCAATAATTACAATGCTCTATCCCCAGCACGACGGAGTTTCACAAGATTACCAAGACCTCTCGGCCAAGGTTAGACTCGCTGGCTCCGTCAGTGTAGCGCGCGTGCGGCCCAGAACGTCTAAGGGCATCACAGACCTGTTATTGCCTCAAACTTCCATCGGCTTGAAACCGATAGTCCCTCTAAGAAGTGGATAACCAGCAAATGCTAGCACCACTATTTAGTAGGTTAAGGTCTCGTTCGTTATCGCAATTAAGCAGACAAATCACTCCACCAACTAAGAACGGCCATGCACCACCACCCACAAAATCAAGAAAGAGCTCTCAATCTGTCAATCCTTATTGTGTCTGGACCTGGTGAGTTTCCCCGTGTTGAGTCAAATTAAGCCGCAGGCTCCACTCCTGGTGGTGCCCTTCCGTCAATTCCTTTAAGTTTCAGCCTTGCGACCATACTCCCCCCAGAACCCAAAGACTTTGATTTCTCGTAAGGTGCCGAGTGGGTCATTAAAAAAACACCACCCGATCCCTAGTCGGCATAGTTTATGGTTAAGACTACGACGGTATCTGATCATCTTCGATCCCCTAACTTTCGTTCTTGATTAATGAAAACGTCCTTGGCATTTTTTCAAAGTAAAAGTCCTGGTTCGCCAAGAGCCACAAGGACTCAAGGTTAGCCAGAAGGAAAGGCCCCGTTGGAAATCCAGTACACGAAAAAATCGGACCGGCCAACCGGGCCCAAAGTTCAACTACGAGCTTTTTAACTGCAACAACTTTAATATACGCTATTGGAGCTGGAATTACCGCGGCTGCTGGCACCAGACTTGCCCTCCAATTGTTCCTCGTTAAGGTATTTACATTGTACTCATTCCAATTACAAGACCCAAAAGAGCCCTGTATCAGTATTTATCGTCACTACCTCCCCG

>mtRNA_contig_30330bp

CCTTAGAATACTTGGATTTAGAGCCCGACTAAATCGGGCTTGGTATTCGCAATATAAATATATTGTACAGGAATAATTTGATATAAAATGAATATAATATATGAGTTTGACTTTAATACTTTTTTTAATAGGAATCTTAGGATTCGTCTTTAATAGAAAAAATATCATATTAATGCTTATTTCAATCGAAATAATGCTATTATCTATAACATTTTTAATATTAGTAAGCTCTGTAAATATGGATGACATAATAGGGCAAACTTATGCTATATATATTATAGTTATTGCTGGAGCTGAATCCGCAATAGGTTTAGGTATTTTAGTGGCTTTTTATAGATTAAGAGGAAGTATAGCAATAGAATATAAATAATGTATTTAAGTATAATTTTTTTACCCTTATTAGCATCAATAGTTTCCGGATTTTTTGGAAGAAAAGTTGGAATTAGTGGAGCACGTTTTTTAGGTTGTTTAAGCATAATAATTACAACAACTTTAGCTATCGTGGCATTTTTTGAAGTAGGATTTAACAACAGTCCTGTGTATTTACATTTATTCCCTTGAATAAACAGTGAATCATTTAACATTGTTTGAAGTTTCCAATTTGATAGCTTAACAGTTTCAATGTTAATTCCTGTATTAATAATTAGTTCTCTGGTTCATATATACTCTATAAGTTATATGAGTGCAGATCCTCACAATCAAAGATTTTTTAGTTATTTAAGCTTGTTTACTTTTATGATGATCATACTTGTAACAGCTAATAATTATTTATTAATGTTTGTTGGATGAGAAGGTGTTGGGGTTTGTTCATACCTTTTAGTTAGTTTCTGATTTACTAGAATAGCAGCTAACCAAAGTTCTATGTCTGCCTTCTTAACAAACAGAGTAGGAGATTGTCTTTTAACTATAGGTATGTTTGCTATCTTATGATCTTTAGGTAACTTAGACTATACTACAGTATTTTCATTAAGTCCTTATATCAATGAAAACATTGTTACTATAATAGGTATATGCTTATTAATAGGTGCTATGGCAAAAAGTTCTCAAGTAGGTCTTCATATTTGATTACCTATGGCTATGGAGGGTCCTACACCAGTTTCTGCGTTAATTCACGCAGCTACTATGGTAACTGCAGGAGTATACTTATTAATACGTTCATCTCCTTTAATTGAATATAGCTCTACTGTATTATTAATATGTTTATGATTAGGTGCAGTAACAACTGTATTTAGTTCTCTTGTAGGTTTATTCCAACAAGATATTAAAAAAATAATTGCTTATTCTACTATGAGTCAATTAGGTATGATGGTTATTGCTATTGGTTTATCTTCTTATAATATAGCTTTATTCCATTTAGTTAACCATGCTTTCTATAAAGGATTATTATTCTTAGGTGCAGGTGCAGTTATTCATGCAGTAGCTGATAACCAAGATTTAAGAAAATACGGTGGATTAGGAGCATTCTTACCATTAACTTATTCTGTTATATTAATAGCAAGTCTTAGTTTAGTAGCTTTCCCTTTTATGACAGGATTCTATAGTAAGGATTTCATATTAGAATCCGCTTATGGACAATATTACTTTAGTAGTATTGCTGTTTACGTTATTGCTGTTATAGGTGCAATATTTACTACTTTATATTCTGTGAAAGTTTTATATCTAACTTTCTTAGCTAACCCTAACGGTAATATAGTTTCTTATAAACATGCTCATGAAGGTGATATATTCTTAAGCCTACCTTTAGTAGTATTAGCTATTTTCTCTATATATTTTGGATATATAACTAAAGATATTTTTATAGGTTTAGGTTCAGGATTCTTTACAGATAACAGTATATTTATTCACCCTATACATGAAATATTAATAGATACAGAATTTGCTGTGCCTACTACATTTAAATTATTACCTTTATTCTTTACAGTATCATTTACAGCTATAGCTATAATTTATTCAGAATTCTTTCCTAATTTAATAAACAGTTTTAAACTATCTAATTTAGGTCATACTATTTATGGATTCTTTAACCAACGTTTCTTAGTTGAGTATTTCTATAATAAATTTATTGTAAACTTAGTTTTAAATATGGGAGGTCAAACAACTAAAATTTTAGATAAAGGTAGTATAGAACTTTTAGGTCCTTTTGGTCTAGAAAAATTATTAATTAAAATTAGTAAAATTATTTCTAGTCTGAATACAGGTGTTGTTACAAATTATGCCTTATTTATATTAATAGGATTTATTGCATATACATCTATATACTACTCTTTCCTTCAAAACTTAGATTTATCTTCTTTAGCCTTACTATTTTCAATTAGTATTTTAACACTTTACAATACTAAAAACAATAACAATAATTAATAATGGAAGTTAGGTTTTATGTGTTGGACTACGTAGAGTAATTTACATTCGTCGAAATGCTCCTAGGGTGGATCCCCTAGCTTCATGAGGCGTAATGGAAGTTTTGAGTGTACTAACTTTTTTAACCGTCTATGTTTTAGGCATCGCTCGGTGGGAAAAGATGCTTCGCTCGAAATCACGATAGGATAGTATTTTATAACTAAGTATGAATTTTATACATTCATATATTTAAAACAAAGGAGAGTTCCTTTATTGGCAATATAAGTAGTTGTTCGTTATTAACTTGGAATTATTAGTGATGAATATAAAAAGTGAAACGCCATCGCTCTTCATCATACTTTGCGGTTTTATATAAGTGGTGTTTTAAGGTGTCTCATATAAAATAATTATATTTAGGCTGGAAGCCTAATTTATGTTATTGCTCGAATTTAATTGTATACACTACATTATAGATTTATCTAGTATATATGTAAGAAGGCCTAAAGGCTTCGAGGAAGCCGTCGTTACGCCTCTTATTTAATTTCTATTTAAACTCTAAATATTTATTTGTATTATTAGGCCTAAGTAAAATTAATATACTATTATAAATATATTTTTACTCTAATTAATTTATAAACTTAGATTAGCTTTTGCTATTTGACAGCGAAGCGCCATTACAAATAAATATCCAAAAATTTATTTTTATTAATCAAATATGAGAATTTTAAAAAGTCATCCTTTATTAAAATTAGCTAATTCGTATCTTATAGATACATCACAACCTACTAATATTAGTTATCTTTGAAACTTCGGTTCTTTACTTGCTGTTTGTTTAGGTATACAAATAGTTACAGGAGTAACTTTAGCTATGCATTACAACCCTAGCATTGCTGAAGCATTTAACTCTGTAGAACATATTATGAGAGACGTAAATAATGGATGATTAATACGTTATTTACATAGTAATACAGCTTCTGCTTTCTTCTTCTTAGTTTACTTACATGTAGGAAGAGGTATGTATTACGGTTCATATAGAGCTCCAAGAACATTAGTTTGAGTTATAGGTGCTATAATACTTGTAGCTATGATGGGTATAGGTTTCCTGGGTTATGTTTTACCATATGGACAAATGTCTTTATGAGGTGCAACTGTAATAACTAACCTTATTAGTGCTATACCATGAATTGGACAAGATATTGTTGAGTTCATTTGAGGAGGTTTTTCTGTTAATAATGCAACTTTAAACAGATTCTTTGCTTTACACTTTGTATTACCTTTCGTATTAGCTGCATTAGTTTTAATGCATTTAATCGCTTTACATGACACAGTAGGATCAAGTAACCCATTAGGTGTTTCTGGAAATTATGATAGAATACCTTTTGCTCCTTACTACCTATTTAAAGACTTAATTACTATATTTATGTTTGTATTTGGATTAAGTCTATTTGTATTCTTCATGCCTAATGTCTTAGGAGATAGTGATAATTATATAATGGCTAACCCTATGCAAACTCCAGCTGCTATAGTTCCAGAATGATATTTATTACCTTTCTACGCTATATTAAGATCTATCCCTAATAAATTATTAGGAGTTCTTGCTATGTTTAGTGCTATATTAATTATATTAACATTACCTTATACAGACTTAGGGGAAACTAAAGGTTACCAATTCAGACCTTTAAGTAAAGCATTCTTCTATGTATTTGTGGCTAATTTCTTAATATTAATGCAATTAGGAGCTAAACATGTTGAATCTCCATTTATAGAATTAGGGCAAATCTCTACAGTTTTATATTTCTCTTACTTCTTAGTTATAGTACCTTCTGTAAGTTTAATAGAAAATACTGCAAAATATGTAGCAACTGCTAGATAAAAATGAGTTATATCATTGTTTTTCTTAGGCCTGAAGATTATATGTCAAATGGTAATTTAATATTTAAAATATATTAAACTAATACTGTGGTTTAAGTCGCAGTCTAATTTAAACAATATGAAGAAGTCGAGGATATTATGTCTGAGGCTAAGTGAATGTTAGAATAATAAAGTTTTATTCAAATTAAAAACTCAAAGATTGTGAAGTAGATGGTCTACACTTTGACTGCAAATCTTAAGTAAGAGGTTCGATTCCTCCATGATCTTAGGACTAAAGTCTAATTTATTTTCGTACCATTATAGCTCTTTATGTCTGTCTCGTATAGAGGTTATTTATCAAATAAAAAAGCTTATAAGAAATATGACTTAAAATTTAGTTATATTGGAAAATATAGTAGTGTATATCGAGATTTTTATAAGTATATTATAAGTCTAAGTTAATATAATTAACTGGGTCTTTTAATATAAAAAACATTATCTGAGTATAGATTTTGTTTCTGGTATAATCAATATAATCAGTTAATTCGGAAGGTGAAGAAGATTTAGCTTCAGAGTCTAATAGTGAGTCATCTAATTCGGAAGGTGAGGAAGAAGCTCCTTCGCATAGTGAATTTAATGAGTCAGTTAATTCGGATAGTCAAGGGGAAGCTGATTCGGATAGTCAAGGGGAAGCTCCTGCCAATGGTGAATCTAATAATGAGTCATCTAATTCGCAAGGTCAAGGAGATTCTAATGGTAGTGAAGAAAATAACAAACGTCCCTTATCATCTTATTCAGATGATAATCCTACCAAAAAATTAGACAATAAGCAATCACCTTTAGACTATGTACTGGAAAAACAGGCAACGGAAATGCCTGACATCCAGGATGCCGATGGTGGTGATTAAGCATTTATATGGTTTATTTTATAATTTATAATAGTTTCCTATCCTTTGCTTTCTGGGTTGCTAAGTAGTAGCTAAGTTCGTCTCCCTTCCTCGGCTATTAGCCAAAATAAATATTTATTAAAAATAAATATTATATGGGGCGCTTAGGCTACGCATGCACGGAGGCATGTACGCAGCCATTAAGATTGTTTAGGTGCAACAGGGTTACTTGTAATATTTAGTTAAAATTTATTATCATAATTTCCTATTATGGTATATATTAAGACTATGTTATATATATATATATTAAAACATGCTGTTATCACTATTAATTATTATACCATTAACTGGTATATTTGTCATATCTAGTTATACTTCTTATGAAAGCGGGTCTTCGCCCACTAACTCTAATGTAGAAAATGTTACACTATACAAACAAATAGCCTTAGTAACTTCTAGTGTAAATATGATTTTATCTTTTATAATTTATCTATTTTTCAATTCAAGTACAAATCAATTCCAATTTGTACAAGAACACTATAATGTACAATTATTTGATATTTATTTAGGGGTAGACGGTATTTCTATCTATTTTGTATTATTAACAACTATAATAATGCCTATTGCTTTATTATCAAATTGAAACTCTATAAAAGAAAATGTTAAATTTTATTTAAATACTATATTATTATTAGAAACATTACTATTAGGGTGTTTCTTAGTTGCAGATATATTATTATTCTATATCTTTTTCGAAAGTATTTTACCTCCTTTATTTTTATTAATAGGTTTATTTGGATCTAGTAATAAAGTAAGAGCAAGTTATTACTTTTTCTTATATACAGTATGAGGATCTTTATTTTTATTATTAGCTATTTTAGCTATGTATTCTATAATGGGTACAACAGATTTTGATGCGTTATTTAAAACAAATTTTGATTATACTACTCAAATTATATTATTTGGTGCTATATTTTTAGCATTCGCTGTAAAAACACCTGTAATATTTTTAAATAATTGACTATTAAAAGCTCATGTTGAATCACCTTTAGGGGGAAGTATTATACTAGCAGGTATTGTTTTAAAATTAAGTTTATACGGTATATTTAGATTAATATTACCTGTATTACCTAAAGCTACATTAGACCTTACTTTTATAGTATACACTATAGGTGTTATTACTATAATATATGCAAGTTTTAGTACATTAAGAACTGTAGATGTTAAAGAATTAATTGCCTATAGTTCTGTGTCTCACGCTGCTGTGTATTTAATAGGTGCATTTAGTAATACAATTCAAGGTATAGAAGGAAGTATTGCTTTAGGATTAGCTCACGGATTTGTATCTAGTGGTTTATTTATATGCGCTGGAGGTATATTATATGACAGATCAGGTACTAGAATGATATATTTATACAGAGGTATAGCTCAACTTATGCCTTTATTTTCTATACTATTCTTTATATTATCTTTAGGTAATTGTGGAGCTCCTTTAACATTAAACTTTATAGGGGAATTTATGTCTCTTTATGGTGTGGTAGAAAAACTACCATTATTAGGTGTATTAGCTTCTACATCTATAGTATTCTCTGCTGCATACACTATATATATGTTTAATAGAATTACATTTGGAGGTTCTTTCACTAAATTCTTTGAAGAAAATATATTTGATACTACAAAAAGAGAATTTACATTATTGTTTATACTAGTTTTATTTACTGTTATACTTGGAATATACCCTTCTTTAATATTAGACAGTTTACACTATTCTGTTGCAAATTTAATTTATAGTTTTTAAGAAACTTCTATATTTTAGAGCTTGCACCTACTTAGAGTATTATATGAATACTTTAAGTATAAACAACTTATATAAAGATTTACTTTACTCAATTTAATATATATAGAAACTAGTATTTAAGGATTAGTTTTTAAGAATATAATTTGAAGTAAACCATTCATTAATTGTTATTTTAACAATTAGTGATACAATTTTTTGTTGTTTAAAGAAGAAGAAGAAGGTAATCAACCAAGATTAACATCTTCCATAGGATTGGGTTTAGAGAGATGATTTTTATCTACTAACGCCAAAGATATTGGAACTTTATATTTAATTTTTGCTTTATTTTCAGGATTATTAGGTACAGCTTTCTCTGTATTAATAAGATTAGAATTAAGTGGACCGGGTGTTCAATATATTTCAGATAACCAATTATATAATAGTATAATTACAGCGCATGCTATACTTATGATATTCTTTATGGTTATGCCTGCCTTAATCGGAGGTTTTGGTAATTTCTTAATGCCTTTAATGGTAGGTGGGCCCGATATGGCATTCCCAAGATTAAACAATATAAGTTTCTGATTATTACCTCCTAGTTTAATACTATTAGTATTCTCTGCATGTATAGAAGGTGGAGTAGGTACAGGGTGAACATTATATCCTCCTTTATCAGGATTACAAAGCCATAGTGGACCTAGTGTAGATTTAGCTATATTTGCTTTACATTTATCAGGGGTAAGTAGTTTATTAGGGGCTATTAATTTTATTACTACAGTGGTAAATATGAGAACACCGGGTATAAGATTACATAAATTAGCTTTATTTGGATGAGCTGTAGTTATAACAGCAGTATTATTATTATTATCTTTACCTGTTTTAGCCGGAGGTATTACAATGGTTTTAACTGATAGAAATTTTAATACATCATTCTTTGAAACAGCTGGTGGAGGTGATCCTATATTATACCAACATCTTTTCTGATTCTTCGGTCACCCTGAAGTTTATATCTTGATTATACCAGGTTTTGGTATAATAAGTACAACTATTTCAGCTAGCTCTAACAAAAGCATTTTTGGATACATAGGAATGGTTTACGCTATGATGTCTATTGGTATATTAGGATTCATAGTTTGAAGTCATCACATGTATACAGTAGGTTTAGACGTTGATACTAGAGCATACTTTACAGCTGCTACATTAATTATTGCAGTACCTACTGGAATAAAAATATTCTCATGATTAGCTACATCTTATGGAGGATCTATAAAATTAACTCCTTCTATGTTATTTGCCTTAGGATTTGTATTTATGTTTACAATAGGAGGATTAAGTGGAGTTGTTTTAGCTAACGCCTCACTTGATATTGCATTCCACGATACTTACTACGTTGTTGCTCATTTCCATTATGTTTTAAGTATGGGTGCAGTTTTTGCAATGTTTAGTGGTTGATACTACTGAATACCTAAAATATTAGGTTTAAATTATAACATTGTATTAGCTAAAATTCAATTCTGAGTTTTATTTATAGGAGTTAATTTAACATTCTTCCCACAACATTTCTTAGGATTACAAGGTATGCCTAGACGTATTAGCGATTACCCTGACGCTTTTGCAGGATGAAATCTAATTAGTAGTTTTGGATCTATTATAAGTGTAGTAGCCGCATGATTATTCTTATACATAGTATACAAACAATTAGTTGAAGGTAAAGTTGCAAGCAGAAATCCATGATCATCTGTACAATATTACACTGATACTTTACAAGCTCTTTTAAATAGAAGTTATCCTAGTTTAGAGTGAGCATTAAGTAGCCCTCCTAAACCTCATGCCTTTGTAAGTCTACCTTTACAAAGTAGCGTTTCTTTTCGTTAGTATATTATGAAAATATATAAACCTTAAAACGTTAACTAAATTATTCATTAGTTTACTATTAGTTTATTTAGTAAAAGACGTATTTTTTGCACTATTTCAAGATATTACTGCTGATATATTACTTATTGGTAGTATATTAGGCTTTTTAACTAGAGCATTTATGTTTACATTATTTGAATATTTAGATAATGAGTTAAAATTACCTTTAACGCTGCGCCGAGATTCTTCTAATTTGAACCCTAAATTTAATAAACCTTTATTCAAAGATAATGGAGAAGGTTCTTCTAAAGATAATACTTATAATGATAGCCCTAGAGACAACGGAGAAGGTTCTTCTAAAGATGCTCCGGCTGCGCCGAAAAGAGGTCGTATTACTTTAGATGACTATACTTTCGATTCGGATAGTGAAGGTACTCGCCCTAAAAAAACTCTAAAAAGATGAGTTGCCCCTAAAATGGATAAGGAGACTGCTGTAGCTAATCTTAGCAAGTATATGGAAATGTATGAAGAATATGAAACTTCAGGAAAAAACGTTCCGGCCGCAAGAGAACAAAAAGCTATGCTGTTACAAAAAATAGAAGAATGTGAATCTATTATAAATGAATCAGATTCTGAATCTGATTCTAAGGATAAAAAAGGTAAAGGCTTGCTTGGCACAAAAAGAAACAACAAATAAAATATTCTAGGCTTAAGCCTGCATATTTTACATATAAATATCTTTATTACTTTGCAAAAAATTTTAGTTCGAATCTGAAATGAGATAGCTCAAAGCTTTGAGTAAGATATCAATTAATAATACCTAATCGTAGGTAAATTTTTTTTACTGGTTTTTTATTTCTATGATACTTTAATAGAATTAATAGCGAGGGCTAACCATACTAAAATATGCTACGTAATGGCGACTTTTTTTCCCTACGTACTACTACGTCACGCCGTACGAAATCGCTTGCGCCTTAATTTTATTATTAAGTATTTATAACACAAAAACCTTTATCTTTATAAATAAATGTATTATACTTCAACTCTTTTATCTGTTTTAGAAGTAATTTTATTGATGTTACCCGCTTTATTAGCAGTAGCTTATGTAACAGTAGCCGAAAGAAAAACTATGGCAAGTATGCAAAGAAGATTAGGACCAAATGCTGTAGGATATTATGGATTATTACAAGCTTTTGCTGATGCTTTAAAATTAATTTTAAAAGAATATGTAGCACCTACACAAGCTAATACTATATTATTCTTCTTAGGTCCTGTAGTTACTTTAGCTTTCGCTTTATTAGGTTATGGAGTAATACCATATGGACCAGGTTTAACTTTAAATGATTTAGAATTAGGTATATTATATATGTTAGCTGTTTCATCATTAGCTACTTACGGTATTTTATTAGCGGGATGAAGTGCTAATAGTAAATATGCTTTTTTAGGTTCTCTTAGAAGTACTGCTCAATTAATTAGCTATGAATTAGTTTTAAGTTCTGCTATACTTTTAATTATCATGATTACTAGTAGTCTTAATTTAAATATTAACATACAAGCTCAAAAAGCAGTATGACTTGTATTACCTATATTTCCTATTTTTTTAATATTCTTTATAGGTTCTGTAGCAGAAACTAATAGAGCTCCATTTGATTTAGCTGAGGCTGAATCTGAATTAGTTAGTGGATTTATGACAGAACATGCTGCAGTTGTATTTGTATTCTTTTTCTTAGCTGAATATGGAAGTATTTTATTAATGTGTATATTAACAAGTATATTATTTTTAGGGGGTTATTTAGTAGGATTTAATTTGTTATACTGATTTAACTTAATAAATAATATATGAGCTTATATATTCGATATAGATTGAGTGCTATCTGCTGAATATAATAACTTAAAAATTATGGTAAGTGCTTTTGCTGAAAGTGGTTTATTTTCTAGTCTTATTTTAGGTGCTAAAAGTTCCCTTTTAGTTTTCATTTTTATATGAGTTAGAGCTTCATTCCCTAGAATACGTTTTGACCAATTAATGTCATTTTGTTGAACAGTATTATTACCTTTATTATTTGCTTTTATTATATTGTTACCTTGTACTTTATATGTATTTGACATCTTTGTCATAAATATTACCATCTAGTACAATATAGATTATTTCAGCCCTATATAAAAAGCTTAGAGTATACTTTGAATACTTTAAGTAATATTAGTTTTTATAACTTTATTAATAAAAATAAAGAGCTAGTAGAAGAGAATGAAACCCCTGTCCCGTAATTCGTAGGGAAAGGTTGATTTTTACCTAATCTGATTAAAAAAAAATGAGTTGGTTAACTTTTTACTATACGCCAGAAGACCGCAAAAGTTAGACTTTGGTAATTAGTAACGCTTATTTTCTTGAAATATAAATAAATAACATGACGTCCTCTCAATCCTATCATCTCTAATTTAGAATGATAGTTCAAGAAAATAGCGAGTGCTTATATCCAAATTATTTTTTTCCATTTTAATTACCGCTATTTATAGAGCGAGGTATTTTATAATACAGTCAGATTATTCTAGCAAGAAGAGCATTCGTCTGTAAGAAGTCACATGCTAACTTAGCAGTAGCGGCGACTTCTTACGCAGGAAGCAAAAAAGGATAAATTATAGGCCCGAGAGGACGAAGGGAATAGCCTATTTTATCTTATTATTTCCCTTTTTAAATAAACAAACAAACCATGAATATGATTTTTAATAAAAATTTAACTTACAAAATTAATGTTACCTCAGCTTTATTTTCTACAACTAGTATTTTTAATATGGCCGACAAAAAATTGCCTAGTCATGTTAAAGATGATATTTCAGAGACTGTTCACGATGAGTATAAAAACACTGTACCTAAGTTTAAAGAGCCAGTTAGAGATGCTTTATTAGATGAGCCTTCTGATAGTTCTAATGAGGCTTTGGATAAATTTTCTAGTTTCGTTACAAAACATTCGGATAAGGGTGAAAAAGTAGCCTCGGAAGAATATTCTAAGTTAGTTGAAAATGTTACTGAGCAAATAAAAGAAAAGTATCCAGAATGTTCAGATGATAGCATAAAAGAGAGAGTGGATAAAGAAGTTCATCATAAGTTATTTAGTCGGGAATTTGAAGTACCTATGTTACCTGGTCAAGAAAGTTGACGTCGTGAGTTACATTATGAGTTTTCTCGCCCTGGCGGTGCCGGTGACCAGGGATCTGATCGTGCTGATTCTATTGCGTCTAATGACTCTGGTACAGGTCAAGGAGGAGCTACATATAAATCAGATGATTTTACAGATCAATCTGTTGTAGGTAGCCCGGGATGTTCTAGTAGAGATGCTCCATCTAACCCTCCTCTTTCATCTTCTTCTGAAGAAGAATTATCTTCAGAGGCTAATAGTGAGTCAGTTAATTCGGAAGGTGAAGAAAATTTATCTTCAGAGGCTAACAGTGAATCGTAGTATAATAATGTATATTTATTCATTATTACCATCTTTATTGTTATTTAATGGAGATGATGATACGGATAAATCTTCGAATAATTCTTATAGTAATGAGGATAGCGAAGGTTGTTCAGGCAATGGATCCGATGAGGATCCCGGCGATGAAGACGGTAAGAAAACCGTAAACGATGATCCTGAAAAAACCAGAATCATTGAACGGGATAATTCACATCGTGGAGAGAATATCATGGATAGCTTAGAACTTGTAGATAAAGCAAAAGAAGGAGATAAGGATGCTTTAGATACGATTAAGTCCGAATACTCTTCCTTTTTTGACACGACTGATTCGGATAAAGAGGGATTAAATCAAGTAGAAAACTATTTAGAAGAAGAATTTGGTTGCGGAATAGATGAAGAAGCTAAAGAAGCGGATTTATTGGATGCAATTGAAAAGGAAAGGGAAGCTTTAGCTTCCGAATCTAAATCAGGTAATACTCAGTATTCTTCAGCTGAAAGCTCAGATTTTAAGGAAAATTTGCTTACGGATGATTCACGTAAAAGATCACACTCCGAAGAGGATGATTTCGACTCTAAACGTCAACGTAGATCGTATTCGGATGATGTTAATAACAACGGTAAAGGACCAGGTGGTGGATTTTCAGGTCCAAATTCTTCAGGTAATGGGCCTGAGGCTAATGGTCCAGATACTGAATCTCCTACAAGTTCTAATAAAATAATGGAAGTATCACTTGGCTTATTAATATTAGGTGGAGGAATTCTAGAGAATATTGCTGAAACTCTTTGTAATTTATTTTCTTAACTTGTTTTATAAATAGCTTTTTAATAATAAGTTAAATTTGTGCATAGTATTGTTTTTTTCTTTATCCGCCTCCGGCGGATAAAAAACCTCACGTAAAATATATTGCTAATATTCATTCTAGTATTAGCAATATTCATTCTAGCTTTCGTAGCTCAATAAGAATGAAAAACCAAAAAGTTTGAAAAAAAGTCTAAGGAAGCAATATGTTTATTAATTTACATTAAAAATTATTTTTTATATTAGATAGTTTACATTACGGCGGCGAAGCCGCCCTCCCTATCTAACTACATTACCTTTTAGCTTGGCTACAACGTAGCCCCTTCGCACATCTTCTAGTTTTATATTAAACATTAATACTGAGGGACGGTGGTTAAATTCCCCCTACCTAATTTATAAGTATTTAAAAAAAAATTATGAAAAAATGCCTCAATTAGTACCTTTTTATTTTGTTAACGAAGTAACTTTTGCTTTTGCAATAATTGTATTACTTACTTACGTATTTTCTAAATACGTATTACCAAGAATAGTTAGTTTATTTGTATCTCGTCTTTTTATCTCAACACTATTAGATAAATTTGGATTATAATAAATTTTAGATATTTAGATATTTTAAGGCCTATATAAAAAGCTTAGAGTATAATATGAATACTTTAAGTTTAAACAATTTTCATAAAGAAATCTCTAGTCCTTTAACTCAATTTGAAATAAGAGATTTATTAAGTATAGATTTACCTATACTAAGTGATTTACATATATCTATGACTAACATTGGATTATACTTAACAATTGGATTAGTTTTCACATTAATTTTAAGTATATTAAGTATAAATAATAATAAATTAGTTAGCAATAACTGATCAATAAGTCAAGAATCTTTATATGCAACTATACATGGTATAGTAGTAAATCAAATAAATGCAAGAAGTGGTCAAGTTTACTTCCCTTTTATTTACACTTTATTTATATTTATATTAATAAATAATTTAATAGGTATGGTACCTTACAGTTTTGCATCAACTAGCCATTTTGTTTTAACATTTGCTCTTAGTTTTACTATTGTTTTAGGTGCAACTATATTAGGATTCCAAAAACATGGTTTAAAATTCTTTTCATTATTAGTACCAGCTGGTTGTCCTTTAGCTTTATTACCTTTATTAGTATTAATAGAATTTATTTCATACTTAGCTAGAAATATTTCTTTAGGACTTAGATTAGCAGCTAATATATTATCAGGTCACATGTTATTACATATATTAGCAGGATTTACTTACAATATTATGACTTCTGGTTTTATATTCTTCTTTTTAGGATTAGTACCTTTAGCCTTTATAATTGCTTTCTCTGGTTTAGAATTAGGTATTGCATTTATACAAGCTCAAGTGTTTGTAGTTTTAACTAGCGGTTATATAAAAGACGGATTAGACTTACATTAAGGAACAAGTTCATTATTCGTATAATATAGTAGTGCCTTTCTTATGAAAGTATTACCGGGCAAAATTGGTTAATACCAAAATGGATTCTGTGAAATAGAGCGCAGGATTATCTATATTATTTGAGTCATAGAGTATTTAACAAAAATACAGGAGATCTATTGATGAATGGAAAAGAAATAGTAAAATATTCGGAAAAAGAAAAGAGAATATGATACCTTCCTTCTATAGAATCAACAAGAGCTATAATATGACTTTTGAAATACATGGATAAAGAATATTTTGGAGGATATTGTAAAAATAGTATATCAACATTAAACTATAAAGGAAATGAATCTTCTAATGGATGAACAGATTCATTTGAACTGAAGGATTCTATATGAGTAAGATTTCCGATAGATTGACATGTAAAAGGAAGTCTTGAAGAAAGACTACCAGAACTGTTAAACGAGGACCTAACTGTAAATAGAAAGAAAATGAATAAAGTAATTCGGAAGAAGAGACTAGAACCTACCAAAGAATCTAGCCAAAAGGGTATTATATTAATGGTAAAGAAGAGCGTTACGGCCGCTAAAATATGAGTTCAAATCTCATATACCCTTCCATTCCTTATATTATAATCTATAATTAAAATCTAGGTATTAGCAATATATTGTGCGTAATGTTTGTTATTCGATTTATTTCGTACGAAGTTGCGGAGCACGCTAGGCTCGATTAAAGAAAACATCCGTAGGCTGCGAATAAATAAAAAATATGTATGAGTAGGCGAAGTTTACTAACCTATTTTAAATTGACTATATATTAAGTATTTAATAAAAATGGTATTTGTATATAAAACCTTACAAATACCTATATTTAGTGATAAAGAATGAAAAAAGCTATTATTTCTAATCGGGGATTAATTTAGTCAATAAATGAAAATGAAAGAATTATATATATGATCAATGAAAGTATATTTAATTATTAGAAATAAGGTGATTATTTTAGTGAATAAATGAAAATGAAAGCATTTCACCTCAAGAGTAATGTGGCAACGCAGGAACTGAAATCACTAGACCGTTTCTGACACCAGTAGTGAAGTATGTTATTTAATTCGATGATCCACGAAAAACCTTACCACAACTTGAATATCTAGTAAATAGATACAGGAGTTGCACGGCTGTTTTCAGTTAATGTTGTGAAACTGTGGTTTTTCCATGAAATTAACGGAATCCTTTGCTTTATTTATAGAATGTTTTATAGAGCAATCTTTCTTTATAGCGGAAAAGATAAGAAGGAAAAAGACAAGTCATCATGGCCCTTATGTTGTGGGCTATAGACGTGCCACGTAGTCCTTAACAAAGAGATGCGAAAATGCGAATTTTAGCAAATCTCAAAAAAGAGGATATAAATTATACAAGGATTGTAGTCTGAAACTCGACTATATGAATAAGTAATTACTAGTAATCGTGAATCACCATGTCACGGTGAATTTAATCTCGGATTGGTACTAACCACTCGTCGCATGCTGAAAGGAGTTTACGCAATAAGTTTGCTATTTTGTTATAATCTCTTTTAAAATATTATAATGGAGTTCTTCGTATGCGTTACTCTGATTAGTGTTAAGTCGAAATATGGTTCGTGTAGTGGAAGTTGCACGGGGTTTATAAACTTAAAAAATATGATAGCATATAGCCGTTAAAGGGATACAATCCCTGGCGATATGCTAAGTATTATATATATCCATTCAAAGGGGGGGGAGAATTCTCCCCTTGGTTATATGTAAAAGGAGAGTTCCTTTATTGGCAAAAAGGGTTGAGCTGTAAACTCAATAGCTATTTCAGGCTTTAAGAGTTCGAATCTCTTGTCTCCTAAGCTCGCCCGCCTTTATAGCTCAACGGTAGAGCATAATACTGTTAATATTATGATAGAGGTTCGATTCCCCCTTAAGGGCTTGATTATCTTTTATTATGGTGGTAATACCTAGCTTTTCAAAGATTTTATACAAACAATGACAAATTTAAAAAGAAGTAATTTTCAAGATCATCCTTTCCATTTAGTTTCTCCATCACCTTGACCCTTTTATACTAGTCTTAGTTTATTATCTTTAACACTAAATTCTGCACTAGCTATGCATAATTTTACTAACGCTTACCTATTTTTCTATTTAGGTCTATTTTTAGTTGTAAGTTCTATGACTTTATGATTTAGAGATATAATAACAGAAGGTACATTTTTAGGAAATCACACCTTAGCGGTGCAAAAAGGACTTAACTTAGGTGTAATCCTATTTATAGTATCTGAAGGGTTATTCTTTGTGGCTATATTCTGAGCATTCTTCCATAGTGCATTAACGCCTACAGTTGAATTAGGAGCACAATGACCACCTATGGGTATAGATCCTATAAACCCTTTTGAATTACCTTTACTTAATACAATTATATTATTAAGTAGTGGAGCTACAGTGACTTACAGTCACCATGCTTTAATTCAAGGAGATAGAGCTGGAGCTTTATACGGTGCAATAGCTACAGTATTATTAGCTTTAGTGTTTACAGCTTTCCAAGGTGTAGAATATTCAGTTTCATCTTTCACAATTAGTGACGGTGCATTTGGTTCATGTTTCTACTTTGCAACTGGTTTCCACGGATTCCACGTTATAATTGGTACATTATTTATTGGAGTAGGATTATGAAGAATATACGCTTATCACTTAACAGATAACCATCATTTAGGGTTTGAAGGAGGTATTCTTTACTGACATTTCGTTGATGTTGTTTGACTATTCTTATATGTATCTATATATTATTGAGGTTCATAATAATTATATATGTATAGCTGTAGGGATTTTAGTATAATGGTGAATACATATGACTTTTAATCATTATGATATTGGTTCGAATCCAATAGATCCTACTTGTTTACTGATCTTAATATTATTCTGCTAATTAATAGTCTTTAGCTTTATTTATAACAATCTTTTTTAATTATTTAGTTTTCCAATTCTTACCATATGATTTAGCAATTTTAAATAATTCAAATAACGGCTCCGCTTTAAATAATGTGTTATATCATACTTCATTAAATGGTGCTTCGCTAGCCTCCGGCTCCGAAAAAATATTTAATGGTGGCGCAAGCTCTAAAATATCAAGTTTAGGAGCTGAAGGCTCAGGGCTAGCAAGCTCTGATTTATTCTTTGCTACAAGTAGTAGTTGAGATGGTAATTTAATCGAAACAAGTCATATATCTAGTATAGGAAATATATTATATACAAGTCATAGTATGTGATTAATATTAGCAAGTTTTATTTTATTATTAGCTATGATAGGAGCAATAGTAATTACAATAAAACCAAAAAACTGAGGGATTAGCTCAATGGTATATTGGAAAAGCTACGCTAGGGATGTTTGTCCTTCAATATTTTTAAACCCCGACTAAGTCGGGAAGATAAATTAAAATTATTGATAATATATTGGGTTAATTTCAAGAATTACTTATATAAGTAGATTTGAAGCGAAATTTATCTTAGCATAATTATATAAGATAAAATCGTTCAACGACTATAAGGTGAGTTATGCTAACAATAATCCTTTTTAACACCCAACATTTTTATTACCATACACTTATAAAAAAAGATAAAAAGCCAATCAAATTTATTTTTATGAAAGGAAAATCTCTAAATACTGCGACTTCTCTAAGAAGTATGCCAGCTAAAATATTTCATTCTAATTTAAACAATAAGTTTAAATTAATACCATTTAATTCTAGCTTTAATGATACAGGTAGAGTTAAATATTTACCTCCTGTTTCTAAAGAATGAAAAAGTACTATTTATTCATATTATAAAAAAAATATGCAAAATATGCCTATAGATAACTTAAATGCCAATAAAATAATACAAAGTTATTTTAATTTACATTTTGCAAATAATAAATTTATAGGATCAAAATACATATCTCCTAGAAAAAGAAGTTTATTCTTAAAAAAAATCTATGTAAGTAAAATGGAAACAAAACATACTAATTCTAAAGCTATAATAACTTTATACACTATCAATGTAGGGAAAAATATAAAAAATTATCTAAATATTGAGAAAATAAAGTAGTAGATTCATTTAAAGATGGATTAAAGCTTAAAATAAAAGATATGGTCAATAATGGTAATACAAAAAATATTGCTTTAGCCTCTAAAAATGCAGATCAAAAAGAAATTATTTTTTCTATGTATAAACATTATAGAGAGTCGTCTTGATCATTAGGTTTTAATTTATATTTAAGATTATACTTAAGTAGACAATTTAAAAAATCTTATTTTGATAAATTAGATTTATTAAGAAATTATCAATTAAAGAGTAATGGATAGTTTATTAAAAGTAGCTAGATTACCTGAAGTTAACAGAATTCAGGAAAAAGCTAGTATTGTAAAATCTAAAGATTGATCTTTATTAGAAAATATTTATCCGAATTTAAGTTTAGTTTCTATACTAGGCGCTAAGGGTAAATCAGAAGGCTCTTGCTCTTTAAATTCATTATTGGAAGAGAATTTAAAAGATACTAATTCTAATAAATCGCAACGTAGTAGATACTATGGTAATATTTCAAATATAATATTTAATTCTATTAAACATAAAAATTTAGGAGGTATTAGATTAGAAGTTAAAGGAAGATTAACCAAACGTAATAGAGCAGATAGATCTGTATTTAAATTTAAATGAAAAGGAGGATTAAAAAATATAGATTCTTCTTATAAAAAATTATCTACAGTAACATACAGAGGTTATTATAAACCAAATGTAATCTATTCATTATCTACATCAAAACGTCGTGTAGGATCTTTCGCCGTAAAAGGTTGAGTAAGTGGTAAATAATTATATTTAGTAGCCTGACCTAGGTCAGGCTCAGATTACTAGATAAGTTATGGTAAAAATAAAAATGAAGACATAGTCTGAACCATTTTGTGAAAAATGGAAATAAAATTTATTATGATAACATGTAGAACAGGCTAATTTGCGCAAGAGTGGTGCAGTTTTCTTAATATCAACAAATGACTTAGTATCTATATTCCTTTCAATAGAATTACAAAGTTATGGTCTATATATATTAAGTACAATATATAGAAATTCTGAATTATCTACTACTGGAGGTTTAATCTATTTCTTATTAGGTGGATTAAGTTCATGTTTTATATTATTAGGAACAGCTTTATTATACGCAAATTCAGGTACTACTAACTTAGATGGTTTATACGTTATTAATAGTATAAGTGATTTTAGTGATATGACTTACTGATATCAACCATATTATATAAACTTTTCTTTATTAGTTTTTAGTATAGGATTCCTATTTAAAATTAGTGCAGCTCCTTTCCATTTTTGATCTCCTGATGTGTATGACGCTATACCTACTATAGTTACAACATTTGTAGCTTTAGTAGCTAAAATATCTATATTAATATTCTTATTAGAATTAGTTTATTACACAAGCAACAATTTTTCAGATATAAATTGAACTTTTGGTTTAATAATAAGTTCATTATTTTCATTAATTATAGGTACAGTTGTAGGTTTAACACAATTTAGAATAAAAAGATTATTTGCTTATAGTACTATATCTCACGTTGGTTATATGTTATTAGCTTTAGGTATATCAAGTATAGAATCTACTCAAGCTTTCATATTTTATTTAACACAATATACTATAAGTAATTTAAATGCCTTTATGATAATAATAGCTATAGGATTTTCTTTCTATCGTTATGTTACTAATAATAAAGAACATGAGGAACTTTTAGATAAAAATAATTCTCCTGTACAACTAGTTAATCAATTAAGAGGATATTTTTACATAAATCCTTTATTAGCTATAAGTTTTGCTATTACAATATTCTCTTTTATGGGTATACCACCTATGGTAGGGTTCTTTGGAAAACAAATGGTTTTAAGTGCAGCTTTAGATAAAGGATATATATTTATAACTTTAATTGCTATTTTAACTAGTGTTGTGGGTGGAGTATATTATTTAACTCTTGTAAAAGAAATTTTCTTTTATAAACCACAGTATAAAGTTAATCCTTTATTAGAAAATTTAACTTTACACGGAGTATAAAATTCTTTATTTTTGCTTTAGTTTATTTACTATTAGATTTAGAAATTTTAGTTATATATCCATTTGGTGTTAGTGAATACGATAATGGTATATACGGATTAATAGTTGTGCTTATTTTCACTGGTATTATTACTGTGGGGTTTGTATTTGAATTAGGAAAAAACGCATTAAAAATAGATAGTAGACAATCTGTTGTTGATTCTAGTAAAAATTCTCGTTTTCTTTCTACTTTTTCTCAAAATTAATTTAAATTTTTACAAATAGGGTTTTTATGGTATTATGTGTTAGAGCATATATTATAACAGTTAGCTGTTTAGTTGCATTCTCAATAGCTGCTAAATATTCTATTGTTAGATTAATAGAATATTTATTATTTTTTTACCAGGATTTCTATCTTAATAAAAAAGCTTTCGCAAAGACAGCAAAATTTAGCGGTCTTTACCTTGAGTGTAAATAAGTTTACTCTCTATACAAATTCATGGTTTATCCTAATGAGCTTCGGCTACAAAAATTATAAAATGTTCAGTAATAAAGTCTTTTTTTGCCTTTAAAACGGCACTATAAACAAATATCTTTTTAATACATAATACATGATACAAGTTGCAAAAATAATAGGAACAGGTTTAGCTACAACAGGGTTAATAGGAGCTGGAGTAGGAATAGGTGTAGTTTTCGGTGCTTTAATTTTAGGTGTAGCAAGAAATCCTTCAATGAGAGGTCAATTATTCTCATACGCTATATTAGGATTTGCTTTCTCTGAAGCTACAGGTTTATTCGCTTTAATGATGGCTTTCTTATTACTTTACGTTGCATAATCTAACTTTACAGGGTAAAACCCATTTATTTCTAATTCCTTTATTAATAATATAGGAAAAATACGTAAACTTATAATTAAAATATGAATTTATTTATAAAAAGCTTAGCTTATCACTTAGACGCTCCACACGCGTGAGGGATTTATTTCCAAGATAGTGCTACTCCTCAAATGGAAGGGTTAGTAGAATTACATGATAATATTATGTACTATTTAGTATTAATATTATTTGCCGTAGGATGAGTATTATTTTCTATAACAAGATACTTTGTAGAAACTAGATCACCTATTTCTCACAAATATTTAAATCACGGTACTTTAATAGAATTAATATGAACTATAACACCTGCTATTATATTAATATTAATTGCATTCCCTTCATTTAAATTATTATATTTAATGGATGAAGTTAATGACCCTTCAATGACTATTTCAGTAGAAGGTCATCAATGATATTGAAGTTACCAATACCCTGACTTCTTAGATTCTAGCGACGAATTTATAGAATTTGATTCATATATCGTGCCAGAATCTGACTTAGAAGAAGGTGCTTTAAGAATGTTAGAAGTTGATAACAGAGTAATTGTACCTGAAGAAACACATATTAGATTTGTTATAACTTCTGGAGACGTTATACATTCTTATGCTGCTCCTGCTTTAGGTATAAAATGTGATGCATACCCTGGTAGATTAAATCAAGTATCTGCATTCATTAACAGATCTGGAGTATTTTATGGTCAATGTTCTGAAATATGTGGAATATTACATTCTTCTATGCCAATTGTAATAGAATCAGTAAGTTTAGAAAAATTTTTACCTTGACTTTTATCTCAGTAGAATTTATATACTTAACTTTATCTATTCTTGGCAATAATAAACAACCTAAGGAGGAAGAGGTGGATAAAAATAGTATTATATCATTATATTTGAGCATATCGATTATTCGGATGACATGTAAACATAGTTATTTTAGAACTAAATATTAATTTTATATATTTATATATATAAACAAAAGAAAGTTCCTTTATTGGTAAAAAGGATTGAGCTGTAAACTCAATAGCTATGCAATAGGCTTTGTTTGTAATGATCTAGAGATCTTAAGATACAAAGTACTAATTTTAAAGTAACTATTTATTAATATATCTTACATTTTATTAACCTTGTTAGTATCCATGCTTCGTATTCAGTCCCCTTTGGGGAAAGGAACGTGGGTAAGGCCATTAAACTAATAAAGGTGTGCGTAGCTTGCATGCTTCGCCAGTCGAAGCTCTAGGACGTACAAATGAAAGCCTGGTTAGCATAAAAGTAATGCAATTGTTTTGTAATCAATAGACGCAAGCGCGATACTTGCACGGGGCTTAGAAAAAGGATTAGTAGTCAAGTGGTAAAGACATAGCTCTTTCAATGCTACATGCGCGGGTTCGAACCCCGCCTAGTCTAAGCGGATTGATGTAATAGTAACATATTTGACTCATGATCAAATTATAAAGGTGCGAAACCTTTATCCGCATAATAGAGGCTATAGCTTAATTGGTAAAGTGTACTGCTCATGACAGTAAACATAAGTGTTCAACTCACTTTAGCCTTATTCATTACGGAGCATGGCTTAACTGCAAGCTCTAAATCCGAGTGCTGGAATAGGTAGACTTATTAGTGATGTATTTGACATCAACATAATTATAGATGAGTTTGGTGATGGCTCTGAATGAATGCTGTCCAAATGCTTGACACATGCTAATCGTACGATTAATTTAATGAATAATTTTATTAATAAGTGGTGTACAGGTGAGTATAAGATATTTTCACTACCTTAAAGTGAGGGATAAAATCCCTCATAATAATAAAGGCTAGGGCTGTACCCTTGTGTAAGCAAGAGTATGTATGCTTGCCGCTTTAGGATGAAAATTAATATCGCCGAGAGAGGTAGTAGTAAAGGTAATGTCTTCACTAGCCTTAAAATCTCGTAACCGTAACTGAAAGGTTGATCGGTCACATTGGGTCTGAAAAAAGCCCAATGCAAAATTAGTACAGCAGTGGGGAATTTTGGTCAATGGCCTAACGGCTGAACTGGCAATTTGGGTAAACGATATAGAATTGTATCTACAAAAATAAAGTTTTAAGTACGTATTAATAATGATAATATGTATACTAGTCTTGACTAATTACGTGCCAGCAGTCGCGGTAATACGTAAGAGACGAGCATTATTCATATTAAATAGGTTTAAAGGGTACCCAGACGGTCAAAATACCTTAATAAAAAGAAGTATTAGACTAGAGTTATATGTGAGAAGACCGTACTTGAGGTGGAGAGATTATATTCTGTGATACCAAAAGGACTGATAAAGGCGAAGGCTGTCTTTTATGTAATAACTGACGTTGGAGGACGAAGGCATAGAGAACTAACAGGATTTGACACCCTAGTAGTCTTTGCAGACAATGATGAATGCCATGAACTAGATAAAGAATGGCAAAACAAGAAGCTTCAACCTTCGAATTCTTAGTTCGAGTCTAAGTACTCTTGTATAGGTGTGGTGGTCGTGGCATGCATGCTTCGCCAACTTGCACAAAATTCTTTAACTTAACGGTAAAGTACATTTTTGATAAGAATGAAATTCAAGTTCGATTCTTGGAAGAATTAAAAGGTGAGCTAGTTAGTCGCTAACCATGCTAGCGACTACGTATAAAAGAGAATTGACAGAGTGGTTTAATGTGCCGGCCTTGAGTACTGGTATGGGGTTCTCCCATCATGGGTTCGAATCCCATATTCTCTGTTTTGTAGCCTAGGTTGACATGGAGGCGAGCTTCATGTGATTGCCAGAATGTCGAGCCGAATCTTTTGGGTTACGGCTAAGCACAAATTAACCTTTTACAGATTAGGGCCGGGTTGGTTAGGCACTCCTTTTGGGAAGGAGACTAGTTGCGTTCACAAATCAACATGTTGTTTTAGTTAAACTAAAATGAAAACATCTTGTCTGAGATTAAATTCTGTACCTGCTAAGATGTACTCAAATTGCCTACTGTTATAGGTTTCGCAGAAAACCAGCTATCCTAGTCAGTGTTGTCTTTCACTACCTACCACAATTCTTCGGATATCTTTACAACGATAACCCGTTCGGACTTTTATAATCCTGATTGTGGTAAAATCACCAGGCTTCGGGTCTAACTTTAGACACTATTCGTTATTTTAACGATTGGTTTATCTACGCTTTCACTCGCATCTAATGTTAACTTGGTGACCCATTATGCAAAAGGTACGTTCTCACTTATTTATTTAGCTCGAACTGCTTATAAAACTACTAATTCTAATCGTTCTCTCATGATACTATTCACTATCGCTTATGTATTATATTTAGCCTTTGAGGAAGGTTCCCCGAGTCCGACATAGTCGGCTATAAATTCAAACAGTTTCTAAACCATTTTACTTATTAATATATAGGCTTCTCTACTTTCACTCACGCTAATCGTAGAATCTCTTTTGATTTCTTTTCCTGAAGTTACTAAGATGTTTCAATTCACTTCGTTTATTAAAAAACTTCTCCCCCCCCCGTACTGGCTGCTACCCCACAAAATTACCATAGTATATATAGTAAAATATGGACAAGGTGTCGGTATATTGTTTAGATCCGTTAAATTTTCGATGCTTTTAAAACTTAACAAGCGCTCTGTTACGAGGTCTTCAAAATTTGGCTGCTTCTAAGCCCATCTCCTTGTCGACTTTAATAAAAACCTTCTTAATTTCACTTAACTAATAATTTTGGAACCTTAACTCTTGTTCTGGGCTGTTTCCCTTTTGACATACAACCTTATCATTCTATGTCTGATAATTCAAGATCCATCTTTGCCATTCCGAGTCTACATACTCACCGATAAAATCTTCACGATCCCCCGATATATACAAATCAACATGTTGTTTTAGTTTAACTAAAACTCTGCCCATCCATAAGCTTTAGGCGGAGGATTTGATGGTCATGATAATAACTTTCGACCTTTTGCCCAAGGTTGTTCCTTGTTTCCAAGGTTGTTCCTTGTACCCAAGGTGTACCGCTTATCATCTTCTCATCGTGTCCTTCTTATTCGTTACTCATGTCACCATTCTCACTTGAATTGTTTGTTATATATTTCTTCACAGAAGAAATATCTTAATTTAATTCAACGTTCTGCTACCCCACAAAATTACCATATTTTACTATATATACTATGGGTTCGATTCCAACTAACCGTAAGCTATGAACTAATAGCTTAATTGGTAAAGACTTTTCCTGTCACGAAAAGGGATGTCGGTTCGATGCCGACTTAGTTCGATGTCAATTAAATTATGCTATGAACAAGAATCTAGGCCGGAAAGGTCGCCATAGGTAGGGCAAGATATTTGCTAAATATTATGTGAAAACATATGGGCGTTCGAGTCGTCTTCTTTCCGAGACTTTCCATTTATTCTTATTTTTAAATAAGCAATACTTTCCCTAGTAAGGGTAAAATTTATTAATATAATATGACTAACTTATTTATCATAGATGAAATATACACTAAATGATTAGAAATGTAATTGATATAGAATATATACCTATATATAATATAGAAGTATACAAAGAAACTATTATGGAAATCTGGCTATGTATTAAAGGGTATGATTTATTTACTCAAATTAAGAATTTAATATAAAGAATAATTTATACTAACTTTAGTATAGATCATTCTTATAATATAAATTTTTATTTTAAATATAAACCCTAAGAGAAGTTTTTTAATAAACGAAGTGAATTGAAACATCTTAGTAACTTCAGGAAAAGAAATCAAAAGAGATTCTACGATTAGCGTGAGTGAAAGTAGAGAAGCCTATATATTAATAAGTAAAATGGTTTAGAAACTGTTTGAATTTATAGCCGACTATGTCGGACTCGGGGAACCTTCCTCAAAGGCTAAATATAATACATAAGCGATAGTGAATAGTATCATGAGAGAACGATTAGAATTAGTAGTTTTATAAGCAGTTCGAGCTAAATAAATAAGTGAGAACGTACCTTTTGCATAATGGGTCACCAAGTTAACATTAGATGCGAGTGAAAGCGTAGATAAACCAATCGTTAAAATAACGAATAGTGTCTAAAGTTAGACCCGAAGCCTGGTGATTTTACCACAATCAGGATTATAAAAGTCCGAACGGGTTATCGTTGTAAAGATATCCGAAGAATTGTGGTAGGTAGTGAAAGACAACACTGACTAGGATAGCTGGTTTTCTGCGAAACCTATAACAGTAGGCAATTTGAGTACATCTTAGCAGGTACAGAATTTAATCTCAGACAAGATGTTTTCATTTTAGTTTAACTAAAACAACATGTTGATTTGTATATATCGGGGGATCGTGAAGATTTTATCGGTGAGTATGTAGACTCGGAATGGCAAAGATGGATCTTGAATTATCAGACATAGAATGATAAGGTTGTATGTCAAAAGGGAAACAGCCCAGAACAAGAGTTAAGGTTCCAAAATTATTAGTTAAGTGAAATTAAGAAGGTTTTTATTAAAGTCGACAAGGAGATGGGCTTAGAAGCAGCCAAATTTTGAAGACCTCGTAACAGAGCGCTTGTTAAGTTTTAAAAGCATCGAAAATTTAACGGATCTAAACAATATACCGACACCTTGTCCATATTTTACTATATATACTATGGTAATTTTGTGGGGTAGCAGAACGTTGAATTAAATTAAGATATTTCTTCTGTGAAGAAATATATAACAAACAATTCAAGTGAGAATGGTGACATGAGTAACGAATAAGAAGGACACGATGAGAAGATGATAAGCGGTACACCTTGGGTACAAGGAACAACCTTGGAAACAAGGAACAACCTTGGGCAAAAGGTCGAAAGTTATTATCATGACCATCAAATCCTCCGCCTAAAGCTTATGGATGGGCAGAAACCCTTAAAGTAACGGCCTCTAAGTTATAAATCTAAAGGTTTAAACGATGAGAAAATCTTTATTTACTAAATGACAACATTTATAAGTGATAAGTGTGCTGGAAAAAATAGTAAATATATTTTATTAAAGTATATAAAATAAACTAACAAGATGATTATAGAAAGATAATAAGATGAAAAAATAACCGTACCTAGAAACTAAAACAAGTAAGCTAGTAGAGAATACGAAGGCGTAAATGAGCTAACAATCATAAAGGAACTCGGCAAACTAACTACCGTAACTTCGGGATAAGGAGAGCTCATTAGTCCTGATTAATATCGGGTAAAAAGGAAGAAGCATAAAATAGTGTTGTACGACTGTTTAATTAAAACACAGCACTTTGCTAAAAGATTAAAAATCGAAGTATTGAGTGTGATGTCTGCCCGATGCCGGCTGGTTAACGAATTTAACTAAATTCTTAAAAAATTTGGTGTTGAAGGAACCCCCGGTTAATGGCGGCCTTAACGTGAGGGTCCTAAGGTAGCGAAATGCCTTGGCCGTTAAATGCGGTCTTGCATGAATGATGTAACGATACAACAGCTGTCTCTATGATTGACTCAGTGAAATTGGAATAACTGTGCAGATACAGTTTACCTCTAGTTAGACGAGAAGACCCTATGCAGCTTTACTGTTACTAATTATAGGGTATGATGGATTAATTTTCAGATTATAAGGTACATTGATTCAATAAAAATGAAAATCCTTTATTTTTACGTCATACTTCAGAATTAAATTTTTTTACTTCTCTAGTCCCCGGCTTGGCTGGGGACCTTACTTATACCATTTACTTACATATTTGGAACATAGGGGAGAAAATAAAAGAATAACATATTAACCTAGTCTGATTAAAGCCTTTGGTAAGGAACATATTGTTAGGAGACAGTTTATGTGGGGCACAGGCCCCTTAAAGAGTAAAAGGGAGTGTCTAAAAATTATTGGTTATAAGGCGTGCTGGTAAAAGCACCGCTATTATAACGTTGTTTGTGATTTTATGTAGTTTTACTATATTTAATCTTATTAAATTATTCTTATCTTATTACAGATAGGGGTGGATATTAGGTACGATTTTCACTATAGTGAAAGTAGCGGTATAAAAAAATATTATGAAGAGCTCTCCTTTTTTATAAAGGCGGAGCCTTCTAATGTTTTGGGAGTTATTAAGTGACGGCGCGGCATGTATGCTTCGCCAGCCAGCACAAACTTTGTTTGTGCTGGTAGCTCTTAATAATAATGATAAATACCTGAAAAGCTCAACGGCTTATTCGTGCCTTACTGTTTGATTAACAACAAATCTTACAGTTGCGTAAGCAGGGCATAGGATCACAAGATGCAAAAAGGAAAGATCTTGGATTATTGGAAAAGCTACGCTAGGGATAACAGGCTAATTTGCGCAAGAGTGTACAAAATGAGTGCGCGGTTTGGCACCTCGATGTCGGCTTAACTTATCCTCATGGATGCAGAAACTATGTAGGGTACGACTGTTCGTCGATTAAAAAGTTACATGAGCTGGGTTAAATACGTCGTGAGACAGTATGGTTTCTATCTTCTAGAGGGAATTTGAATAAAATAAGGATTAACCTTTGTACGAAAGGAACAGGAAAAACTTAACCTTTTTCCCTTCGGGGGACTAGGTTAGGTTATATTAATTGTTAACCTCTGGTTTACCTGTTGTTTATAAGCCCAATATTAATTTATTATAAAATTTTAGGATTTTTATAAGGGATGTTACTTTCACTTAAGTAAATATATATTATAGGCACGGCAGGAAAGCTAAGTTAGTTAAAGATAAGTGCTGAAAGCATATAGGCACGAAGCTTACCTTAAGATATTTCTAATTCACGTAATATACTATTACGGCATATTATATTATTGCAATAGGCTTTGTTTGTAATGATATTATTATTGCAATAGGCTTTGTTTGTAATGATATCCATCCGAGTGCTGGAATAGGTAGACAGGGTATTCTTAAACAATACTGATGAAATTTCGTAAACGTTCAAGTCGTTTTTCGGATACCATCCGAGTGCTGGAATAGGTAGACAGGGTATTCTTAAACAATACTGATGAAATTTCGTAAACGTTCAAGTCGTTTTTCGGATAATAATGAATAAATCCCTAAGTAGGGGTTCTTCTGCAGCGAAGCCGGGGACATAGTTTAATTGGTAAGACTTTGATTTTGCATATCAACATTTCAGGTTCGATTCCTGATGTCTCCATTTTTTTTTTAATACTTAAGCTCGTGAAGCTCAATTGGTAGAGCAAAATATTGAAGCTATTTTGGTTATAAGTTCAAGTCTTATCTCGAGCATTAAAAAAGGGTGATGATGGAATTGGTAGACATAAACAGTTTAGGCCTGTTGGGTATTTTTTGGTATCTTACCCGTTCAAGTCGGGTTCACCTTATAAAATTATGTTGTAGACTAATGGGTAAGTCATAAATTTTTGATATTTACAATTGAGTGTTCGAATCACTCCAACATAAGGTGGATATAGTTCAATTGGTAGATCGACTGTTTGTGGCACAGTATGTTCCCTGTTCGAACCAGGGTATTCACCCTTATTCGCCGTCTATGGACGGTTTAATCGAGCCTAGCGTGCTCCGCAACTTCGTACGAAATAAATCGGATCATAAGTATCACGCACCCGGATAGTTTAATTGGTTAAAACTTTAATTTCATGCATTAAAAATGGGAATTCGATTTTCCCTCCTGGTTATAAAAACTTAATATGATAATAATTTCAATACTATTTATATTACTTTCCAACGCCGTCACTATAAGACGAGATATTTCAATACTTTTTAATAGAGTAGCTATTTTAGCTTTAGTTTATTGTATTTTACAAGATACAATGAGTTTACATTTAGTTAGTAATGGAATAGGGTTACATGGAGGTTTACTTCATATAACTAATATTACACAAATTTTCCATATCTTTATTTATTTTGTAAGTATATTAATTATACAATTAACAAGTTTTCACCCTAGAAAAGTTTGAGTTCCAGAATACTCTTCTTTAAATCAATTATTGTTTAATAAATTTATTTATTATAGAACAAAAATTATTAACAAAATGGGAG
