# Supplementary material for: Small RNA-Based Antiviral Defense in the Phytopathogenic Fungus Colletotrichum higginsianum
Source: PLoS Pathog. 2016 Jun 2;12(6):e1005640. doi: 10.1371/journal.ppat.1005640 (PMC4890784; doi:10.1371/journal.ppat.1005640)
Supplement: S2 Text — (DOCX) [file ppat.1005640.s002.docx]

**S2 Text. Supplementary Methods**

**Targeted gene replacement of *C. higginsianum* RNA silencing genes**

A Targeted Gene Disruption (TGR) approach was followed to obtain *C. higginsianum* RNA silencing-mutant strains. TGR strategy for *RDRs, DCLs and AGOs* is shown in S3, S4 and S5 Figs, respectively. First, two sets of gene specific primers (F1/R1 and F2/R2) were used to amplify the 5’ and 3’ flanking regions of the targeted sequence for the gene of interest (approximately 1.1kb and 1.3kb respectively). This first PCR used *C. higginsianum* IMI 349063A genomic DNA for each RNA silencing gene. A second PCR was performed using *hph-F* and *hph-R* primers and the plasmid *pSILENT* [1] to obtain the *HPH* cassette which confers hygromycin resistance (S3 Table). The 5’ ends of the primers R1 and F2 share reverse-complementarity with the *hph-F* and *hph-R* primers, respectively. Thus, a third PCR reaction was performed to combine and amplify the three PCR products using the external primers F1 and R2. The final product was a target gene replacement cassette for each specific gene with the Hygromycin resistance marker between the two flanking genomic sequences. This PCR product was introduced in the entry vector pENTR/D-TOPO (Invitrogen) and cloned in a next step into the pGKO2-dest by Gateway LR recombination. The resulting pGKO2-dest vector was transformed into *A. tumefaciens* AGL-1 cells and *C. higginsianum* WT conidia were transformed by *Agrobaterium tumefaciens*-mediated transformation Double Selection (ATMT-DS). The pGKO2-dest vector contains the HSVtk (herpes virus thymidine kinase) gene; this protein transforms nucleoside analogs, such as 5-fluoro-2’-deoxyuridine (F2dU), into a toxic compound for the cell [2,3]. Thus successful transformants that undergo homologous recombination contain a disrupted target gene, are Hygromycin-resistant and can grow in the presence of f2dU. For selection of desired transformants 150 µg/mL of Hygromycin and 0.5 µM F2dU were used (S14A and S14B Figs). The double deletion mutant ∆*dcl1*∆*dcl2* was obtained by using the same strategy, however a Phleomycin resistance gene was used. The *DCL1* specific primers (F1/R1(Phle) and F2(Phle)/R2) were used to amplify the genomic regions, while the Phleomycin cassette, conferring phleomycin resistance, was obtained by PCR using the *phle-F* and *phle-R* primers and the plasmid *pBC-Phleo* [4]. The three PCR products were again combined and amplified using the external primers F1 and R2. To obtain the double *dcl* mutant and *C. higginsianum* ∆*dcl2* conidia (HygR) were transformed by ATMT-DS. For selection of desired transformants 200 µg/mL of Phleomycin and 0.5 µM f2dU were used in PDA pH 8.5 plates.

**Binary plasmids generated in this study for *A. tumefaciens*-mediated transformation**

Two binary vectors with the dominant selection marker phleomycin (phle) were created for transformation of fungal strains that are already hygromycin resistant: *pC-PHLE* and *pC-PHLE-Dest*, The Phleomycin resistance cassette (*GDP.prom-Phle-Cyc1*) was PCR-amplified from *pBC-Phleo* [4] using the primers *gdp.prom-HindIII-F* and *cyc1.ter-SpeI-R*, which contain a *Hind*III restriction site at the 5’ end of the *Aspergillus nidulans* *gpd* promoter and a *Spe*I restriction site at the 3’ of the *Saccharomyces cerevisiae CYC1* terminator. The PCR fragment was initially cloned into the *PCR®-Blunt* vector (Invitrogen), then a *Hind*III/*Spe*I digestion was used to isolate the fragment which was then ligated into a *Hind*III/*Avr*II-digested *pCAMBIA0390* vector, creating the *pC-PHLE* vector (*pCAMBIA0390: GDP.prom::Phle::Cyc1)*. The *pPHLE* T-DNA contains the *Phleomycin* cassette and a MCS for cloning purposes. To obtain the destination vector *pC-PHLE-Dest*, a Gateway destination cassette (*att*R1-*ccd*B-*cam*R-*att*R2) was isolated from the plant transformation vector pMDC99 using a *Hin*dIII/*Spe*I digestion and ligated in a *Hin*dIII/*Spe*I-digested *pC-PHLE*, creating the *pC-PHLE-Dest* vector.

**Generation of *C. higginsianum* tagged-AGO constructs**

To generate *AGO1prom-6His-3FLAG-AGO1*, the sequence coding for a 6His-3FLAG epitope was inserted at the N-terminal of the AGO1 sequence by PCR in 2 consecutive steps. First, the *AGO1* open reading frame and 3’ regulatory sequences were PCR-amplified from *C. higginsianum* genomic DNA using the *AGO1-1F-ERV-F* and *AGO1-Pst-R* primers (S7 Table), where the forward primer contains a 1xFLAG sequence in frame with the second codon of the gene. The *1FLAG-AGO1-UTR* PCR product was cloned into *PCR®-Blunt* vector. In a second PCR, the AGO1 native promoter (*AGO1prom*) including the first ATG codon was PCR-amplified from *C. higginsianum* genomic DNA using the primers *AGO1-PROM-F* and *AGO1-PROM-6H2F-ERV/Pst-R* (S7 Table). Here, the *6His-2FLAG* epitope sequence is present in the reverse primer, in frame with the first ATG codon of *AGO1*. The *AGO1prom-6His-2FLAG* PCR product was initially cloned into *pENTR/D-TOPO* (Invitrogen) from which it was isolated using a *Not*I/*EcoR*V digestion, then ligated into the *1FLAG-AGO1-UTR* vector digested with *NotI/EcoRV*; this created the vector *pCR®-Blunt-AGO1prom-6His-3FLAG-AGO1-UTR*. Finally, the *AGO1prom-6His-3FLAG-AGO1-UTR* cassette was isolated using a *Xba*I/*Spe*I digestion and cloned into a *Spe*I-digested *pPHLE* vector to create the binary vector *pC*-*AGO1prom-6His-3FLAG-AGO1-UTR (PhleR).*

To generate *AGO2prom-6His-3FLAG-AGO2*, a sequence coding for a 6His-3FLAG epitope was cloned at the N-terminal of AGO2 by PCR in 2 steps. In a first reaction, the AGO2 native promoter (*AGO2prom*) including the first ATG codon was PCR-amplified from *C. higginsianum* gDNA using the primers *AGO2.prom-HindIII-F* and *AGO2.prom-EcoRI/AvrII-R*, cloned into *pENTR/D-TOPO.* In a second PCR reaction, the AGO2 open reading frame and 3’ regulatory sequences were PCR-amplified from *C. higginsianum* gDNA using the *AGO2-6H3F-EcoRI-F* and *AGO2-AvrII-R* primers (S7 Table). Here, a 6His-3FLAG epitope sequence was present in the forward primer in frame with the second codon of *AGO2*. The *6His-3FLAG-AGO1-UTR* PCR product was cloned into *pENTR/D-TOPO* vector, then isolated using a *EcoR*I/*Avr*II digestion and cloned into a *EcoR*I/*Avr*II-digested *pENTR/D-TOPO:AGO2 promoter* vector, to obtain the vector *pENTR-AGO2prom-6His-3FLAG-AGO2-UTR*. Finally, the *AGO2prom-6His-3FLAG-AGO2-UTR* cassette was cloned by Gateway LR recombination into *pC-PHLE-Dest.*

Integration of the 6H3F-AGO1 and 6H3F-AGO2 constructs were confirmed in several independent transformants by Southern blot. Transformants selected for further analysis had a single copy integration (S8B Fig) and no observable phenotypic defects in vegetative growth (S8C Fig). Introduction of *6H3F-AGO1* in the *∆*a*go1* background (independent transformants 1 and 3) complemented the defects in conidiation (S8D Fig) previously observed *∆*a*go1* mutants. Western blot analysis from total protein extracts using the anti-FLAG antibody determined that 6H3F-AGO1 accumulated in the ∆*ago1*/6H3F-AGO1 strains (S8E Fig, upper panel), and the lower levels of AGO1 protein observed in *∆*a*go1/6H3F-AGO1* strain 2 could explain the partial recovery in conidiation observed for this particular transformant. Immunoprecipitation fractions demonstrated enrichment for 6H3F-AGO1 (S8F Fig, upper panel). On the contrary, 6H3F-AGO2 was not detected in either the total protein or the immunoprecipitated extracts of the ∆*ago2*/6H3F-AGO2 strains (S8E and S8F Figs, lower panel), in agreement with the extremely low level of *AGO2* transcript in mycelia (Fig 1E). Thus, to identify small RNAs loaded into AGO1 from mycelia, we generated small RNA libraries from the input and the AGO1 immunoprecipitated fractions from three independent transformants of ∆*ago1*/*6H3F-AGO1*. Additionally, we sequenced the input and immunoprecipitate fractions from two independent transformants of ∆*ago2/6H3F-AGO2*, and a single replicate of a mock-IP, which did not contain a tagged AGO protein. The total reads sequenced in the input and immunoprecipitate fractions of ∆*ago1*/6H3F-AGO1, ∆*ago2*/6H3F-AGO2 and mock-IP is reported in S3 Table.

**References**

1. Nakayashiki H, Hanada S, Quoc NB, Kadotani N, Tosa Y, et al. (2005) RNA silencing as a tool for exploring gene function in ascomycete fungi. Fungal Genet Biol 42: 275-283.

2. Khang CH, Park S-Y, Lee Y-H, Kang S (2005) A dual selection based, targeted gene replacement tool for *Magnaporthe grisea* and *Fusarium oxysporum*. Fungal Genet Biol 42: 483-492.

3. Tucker SL, Besi MI, Galhano R, Franceschetti M, Goetz S, et al. (2010) Common Genetic Pathways Regulate Organ-Specific Infection-Related Development in the Rice Blast Fungus. Plant Cell 22: 953-972.

4. Silar P (1995) Two new easy-to-use vectors for transformations. Fungal Genet Newsl 42: 73.
