# Supplementary figures and images for: Small RNA-Based Antiviral Defense in the Phytopathogenic Fungus Colletotrichum higginsianum
Source: PLoS Pathog. 2016 Jun 2;12(6):e1005640. doi: 10.1371/journal.ppat.1005640 (PMC4890784; doi:10.1371/journal.ppat.1005640)

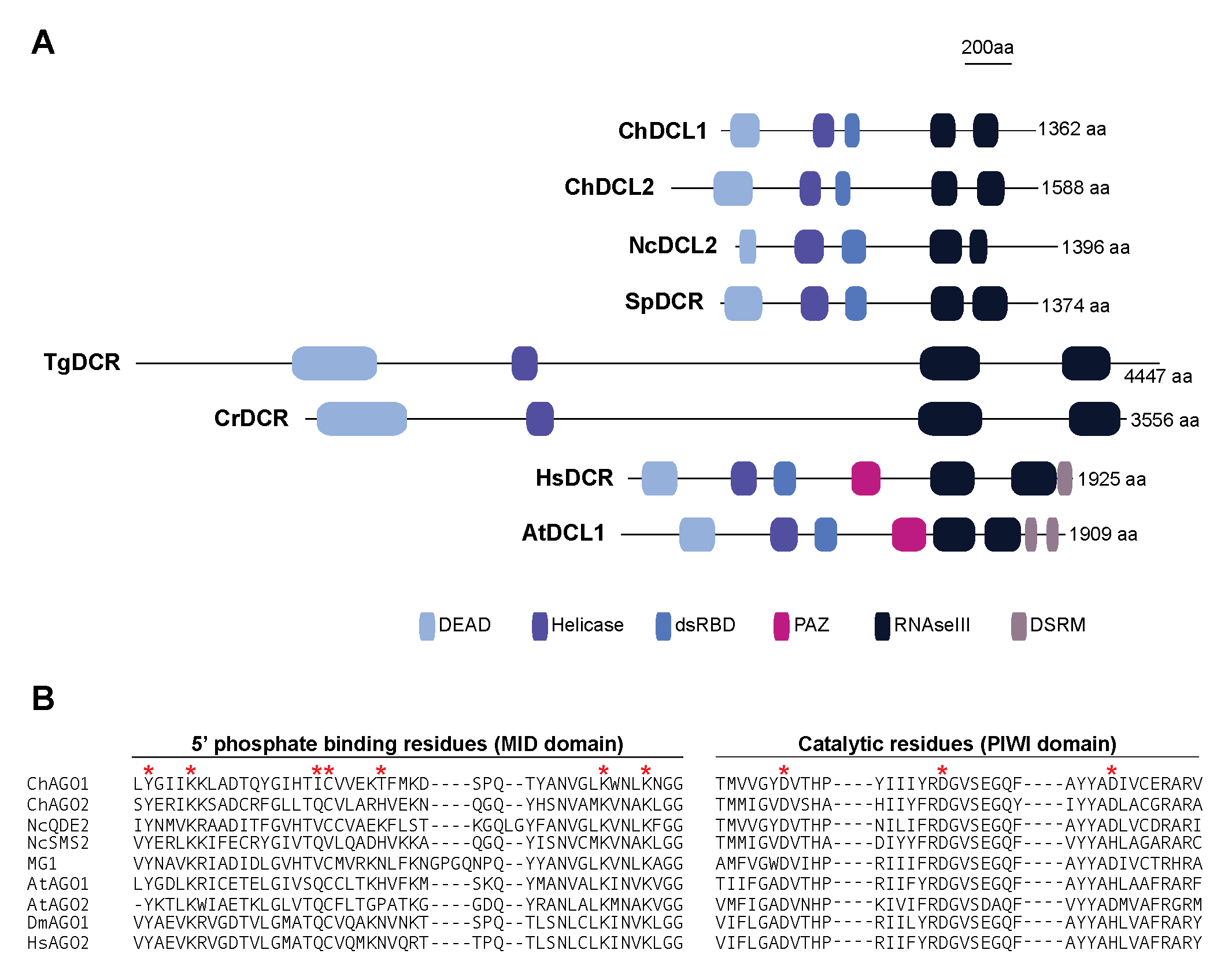

Supplement: S1 Fig — (A) Domain arrangement of Dicer (DCL) proteins from several species. Accession numbers for DCLs of Colletotrichum higginsianum (ChDCL1: CH063_06582, ChDCL2: CH063_02619), Neurospora crassa (NcDCL2: NCU06766), Schizosaccharomyces pombe (SpDCR: NP_588215), Toxoplasma gondii (TgDCR: TGME49_267030), Chlamydomonas reinhardtii (CrDCR: XP_001692436), Homo sapiens (Hs-DCR: Q9UPY3), and Arabidopsis thaliana (AtDCL1: Q9SP32). (B) MAFFT alignment of amino acid residues involved in the 5’ phosphate binding (MID domain) and slicer activity (PIWI domain) in the selected AGO proteins. Position shown to interact specifically with the 5’ phosphate (MID) and Mg+ coordinating residues (PIWI) are labeled with a red asterisk [111–114]. Accession numbers for AGOs of Colletotrichum higginsianum (ChAGO1: CH063_04066, ChAGO2: CH063_09722), Neurospora crassa (NcQDE2: NCU04730, NcSMS2: NCU09434), Magnaporthe oryzae (MG1: MGG_01294), Arabidopsis thaliana (AtAGO1: U91995, AtAGO2: Q9SHF3), Drosophila melanogaster (DmAGO1: CG6671) and Homo sapiens (HsAGO2: Q9UKV8). (TIF) [file ppat.1005640.s004.tif]

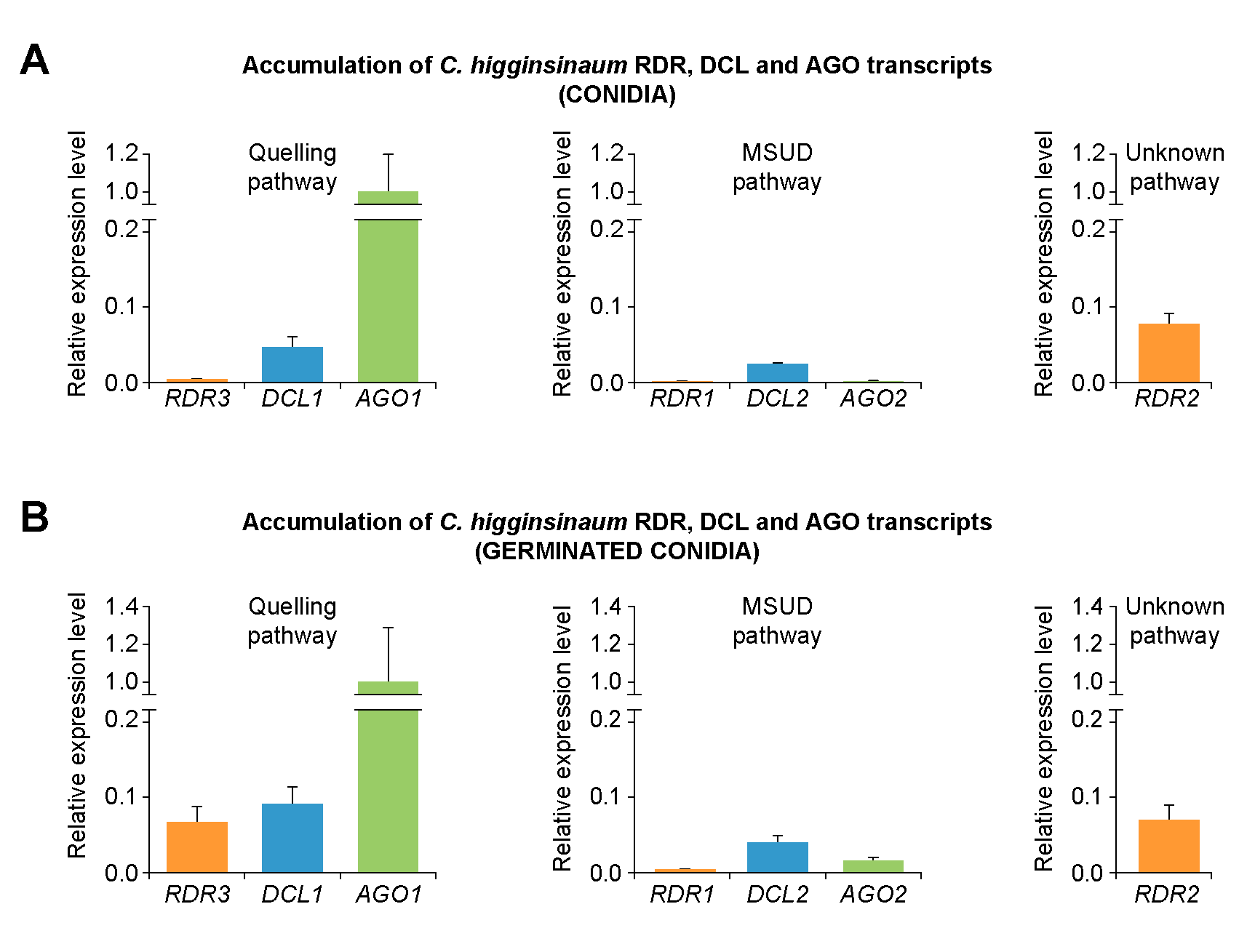

Supplement: S2 Fig — Gene expression in (A) Conidia and (B) Germinated Conidia was analyzed. Silencing genes belonging to the Quelling pathway (left panel), MSUD pathway (middle panel) and Unknown pathway (right panel) are indicated. Values represent means +/- SE of three biological replicates normalized to ACTIN and TUBULIN genes as a relative value to AGO1, as determined by qRT-PCR. (TIF) [file ppat.1005640.s005.tif]

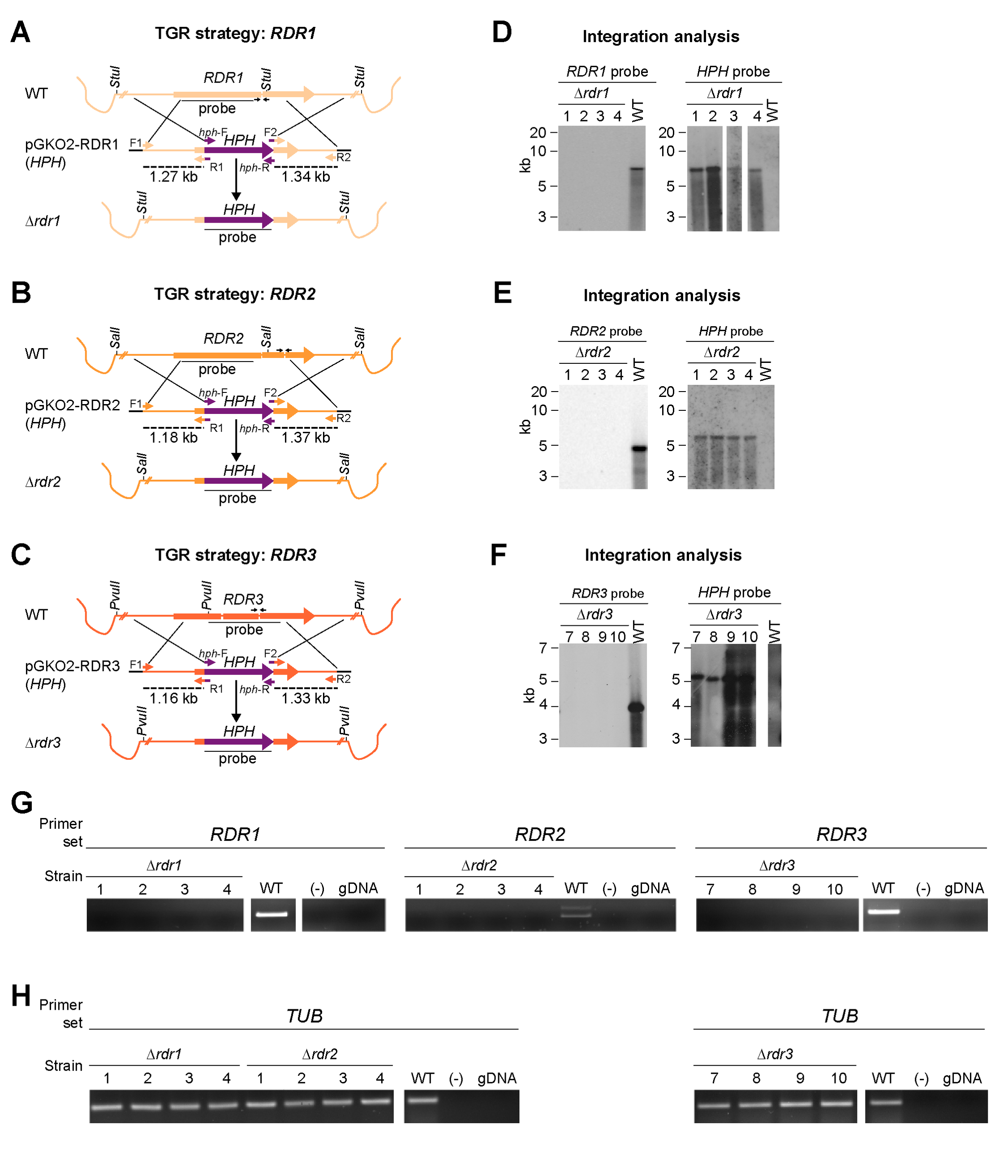

Supplement: S3 Fig — (A-C) Schematic diagram showing the target replacement strategy (TGR) for (A) RDR1 (CH063_02767), (B) RDR2 (CH063_05776) and (C) RDR3 (CH063_08349). Hygromycin (HPH) resistance was used as a selectable marker. Colored arrows indicate primers used for amplification and generation of TGR constructs (S7 Table). For D-H, one gel was used for each gene and probe or primer set; non-contiguous lanes are separated by white space. (D-F) Integration analysis by Southern blot for (D) RDR1, (E) RDR2 and (F) RDR3. One wild-type (WT) and four independent mutant strains were analyzed. (D) A single 7.02 kb band was observed in StuI-digested genomic DNA of WT strains when using the 1.89 kb RDR1 probe (within the deleted region of RDR1). A 7.05 kb unique band was detected in only the Δrdr1 mutant strains when using the 2 kb HPH probe; no band observed in the WT strain. (E) A single 4.90 kb band was observed in SalI-digested genomic DNA of WT when using the 1.90 kb RDR2 probe. A 5.38 kb unique band was detected in only the Δrdr2 mutant strains when using the 2 kb HPH probe. (F) A single 3.99 kb band was observed in PvuII-digested genomic DNA of WT when using the 2.11 kb RDR3 probe. A 5.00 kb unique band was detected in only the Δrdr3 mutant strains when using the 2 kb HPH probe. (G-H) Confirmation of gene knock-out by semi-quantitative RT-PCR analysis of (G) RDR1, RDR2, and RDR3 and (H) tubulin in the corresponding mutant and WT strains. Black arrows in (A-C) denote primers located in exon junctions designed for specific amplification of the RNA transcripts (S7 Table). (TIF) [file ppat.1005640.s006.tif]

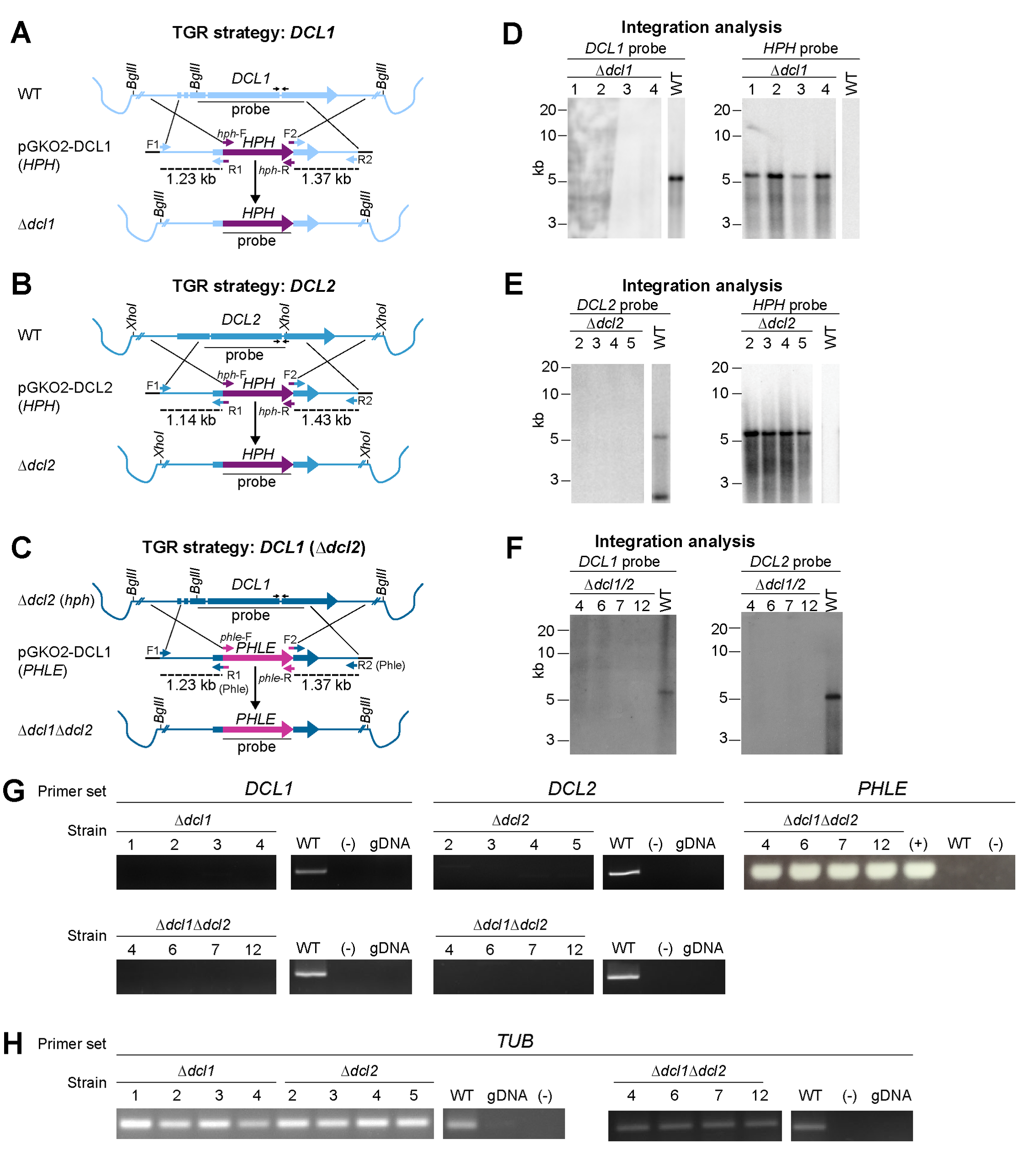

Supplement: S4 Fig — (A-C) Schematic diagram showing the target replacement strategy (TGR) for (A) DCL1 (CH063_06582), (B) DCL2 (CH063_02619) and (C) both DCL1 and DCL2. Hygromycin (HPH) resistance was used as a selectable marker for single mutants (A-B). Phleomycin (PHLE) resistance was used as a selectable marker for generation of double Δdcl12Δdcl2 mutant in the single mutant Δdcl2 background (hph resistance) (C). Colored arrows indicate primers used for amplification and generation of TGR constructs (S7 Table). (D-F) Integration analysis by Southern blot for (D) DCL1, (E) DCL2 and (F) DCL1 in Δdcl2 background. Four independent mutant and wild-type (WT) strains were analyzed. (D) A single 5.34 kb band was observed in BglII-digested genomic DNA of wild-type when using the 3kb DCL1 probe (deleted region in Δdcl1 mutants). A 5.44 kb unique band was detected in all the Δdcl1 mutant strains when using the 2kb HPH probe. (E) Two bands were observed in the XhoI-digested genomic DNA of the wild-type strain as expected; this was due to the initially designed probe, from the deleted region of DCL2, hybridizing across the digestion site. A single 5.58 kb band was detected in all the Δdcl2 mutant strains when using the 2 kb HPH probe. (F) A single 5.34 kb was observed in BglII-digested genomic DNA of wild-type when using the 3 kb DCL1 probe. A 6.41 kb unique band was detected in all the Δdcl1Δdcl2 mutant strains when using the 2.93 kb probe PHLE probe. Disruption of the DCL2 (CH063_02619) gene was re-confirmed using an improved probe that hybridized to a single, unique band in wild-type. (G-I) Confirmation of gene knock-out by expression analysis of DCL1 (G), DCL2 (H), and DCL1 and DCL2 (I) in the corresponding mutant backgrounds as determined by semi-quantitative RT-PCR. Black arrows in (A-C) denote primers located in exon junctions designed for specific amplification of the corresponding RNA transcripts (S7 Table). (TIF) [file ppat.1005640.s007.tif]

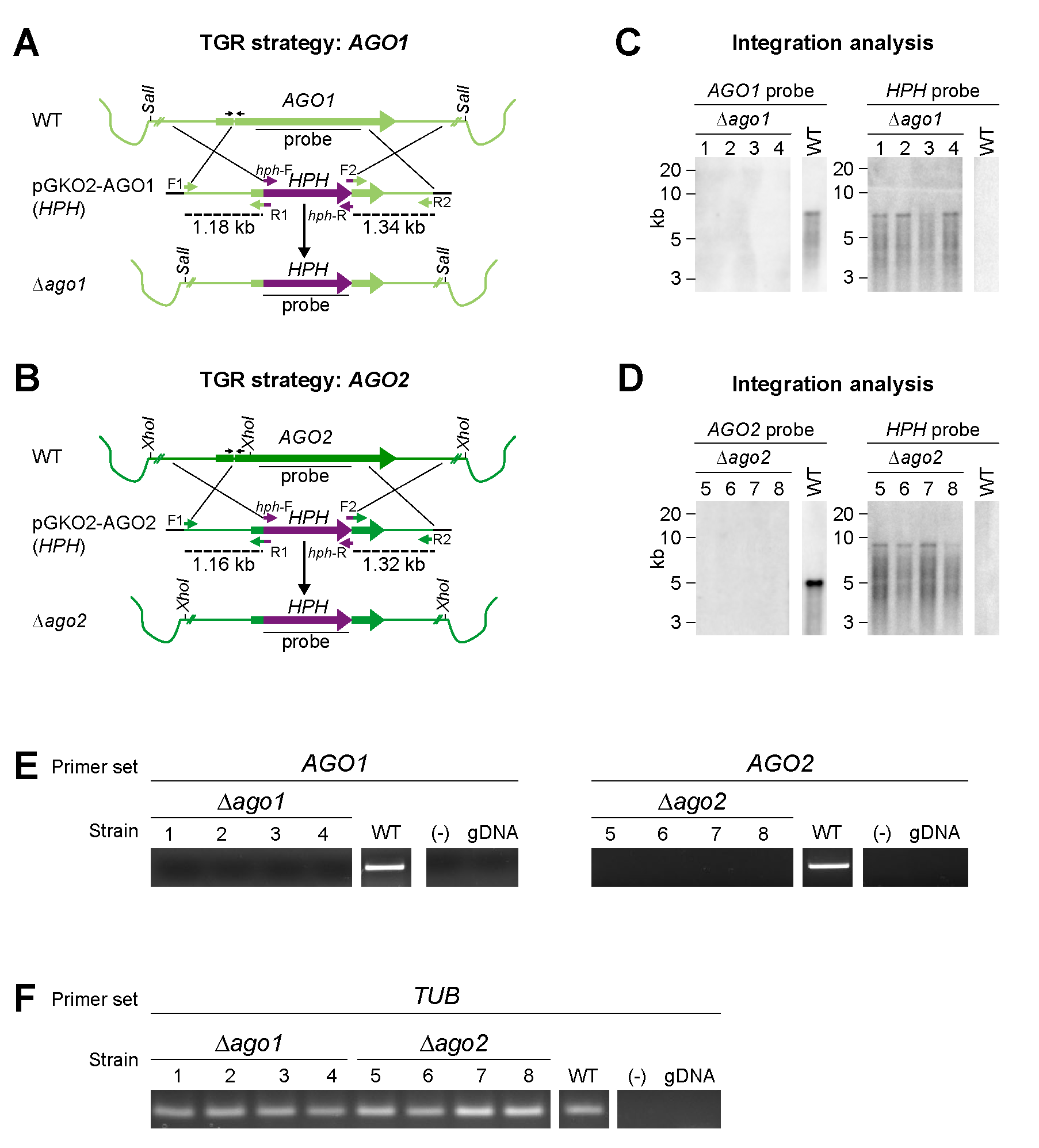

Supplement: S5 Fig — (A-B) Schematic diagram showing the target replacement strategy (TGR) for (A) AGO1 (CH063_04066) and (B) AGO2 (CH063_09722). Hygromycin (HPH) resistance was used as a selectable marker. Colored arrows indicate primers used for amplification and generation of TGR constructs (S7 Table). (C-D) Integration analysis by Southern blot for (D) AGO1 and (E) AGO2. Four independent mutants and one wild-type (WT) strain were analyzed. (D) A single 7.14 kb band was observed in SalI-digested genomic DNA of wild-type when using the 2 kb AGO1 probe (deleted region in Δago1 mutants). A 7.02 kb unique band was detected in all the Δago1 mutant strains when using the 2 kb HPH probe. (E) A single 4.94 kb band was observed in XhoI-digested genomic DNA of wild-type when using the 0.8 kb AGO2 probe. A larger, unique band was detected in all the Δago2 mutant strains when using the 2 kb HPH probe. (E-F) Confirmation of gene knock-out by expression analysis of (G) AGO1 and (F) AGO2 in the corresponding mutant backgrounds as determined by semi-quantitative RT-PCR. Black arrows in (A-B) denote primers located in exon junctions designed for specific amplification of the corresponding RNA transcripts (S7 Table). (TIF) [file ppat.1005640.s008.tif]

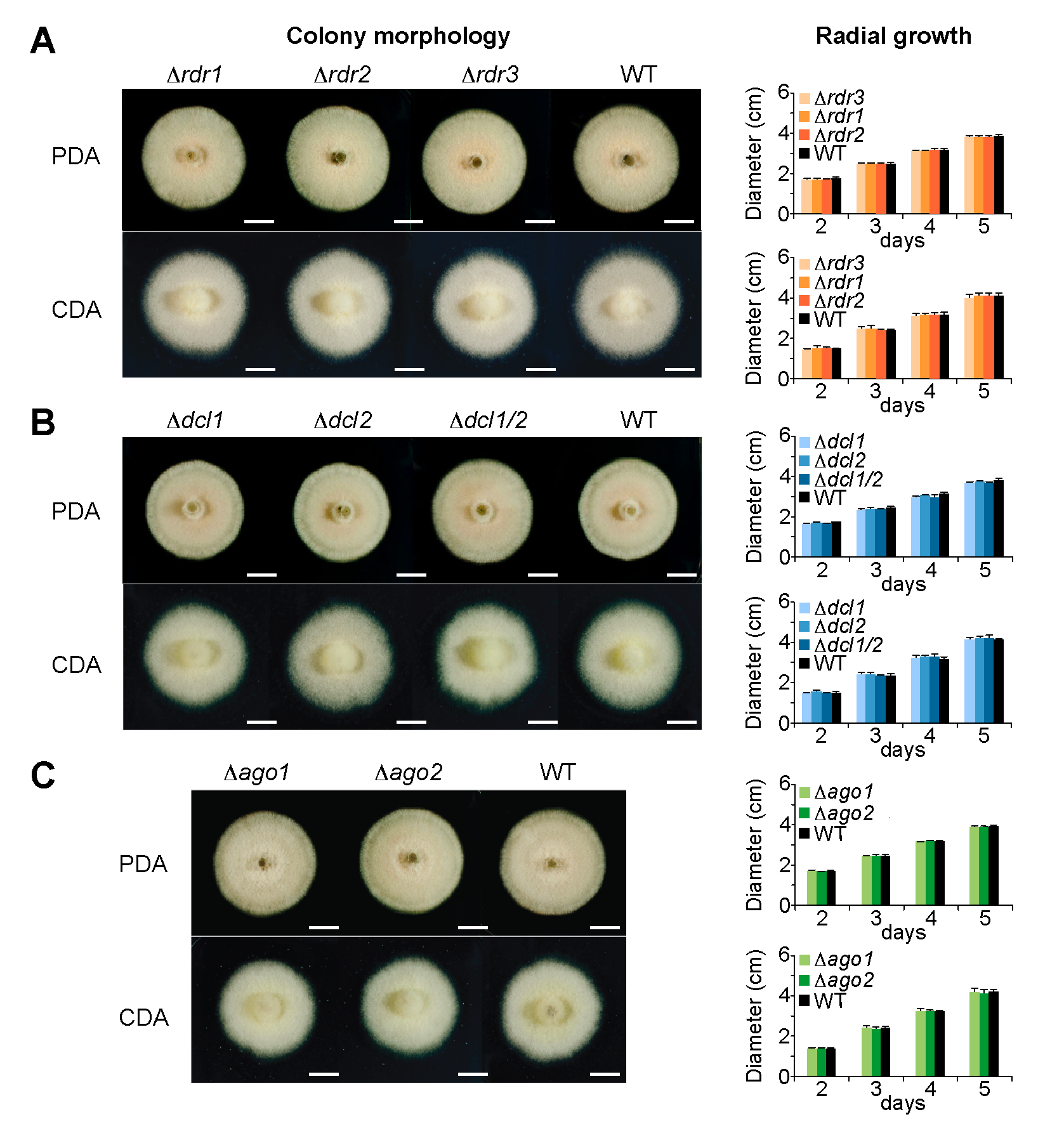

Supplement: S6 Fig — Vegetative growth in the RDRs (A), DCLs (B) and AGOs (C) mutant strains on PDA and CDA media. Representative images of colony morphology after six days of growth (left panel) and measurements of radial growth from 2 to 5 days (right panel) (mean +/- SE). Scale bar = 1 cm. PDA, Potato Dextrose Agar. CDA, Czapek Dox Agar. (TIF) [file ppat.1005640.s009.tif]

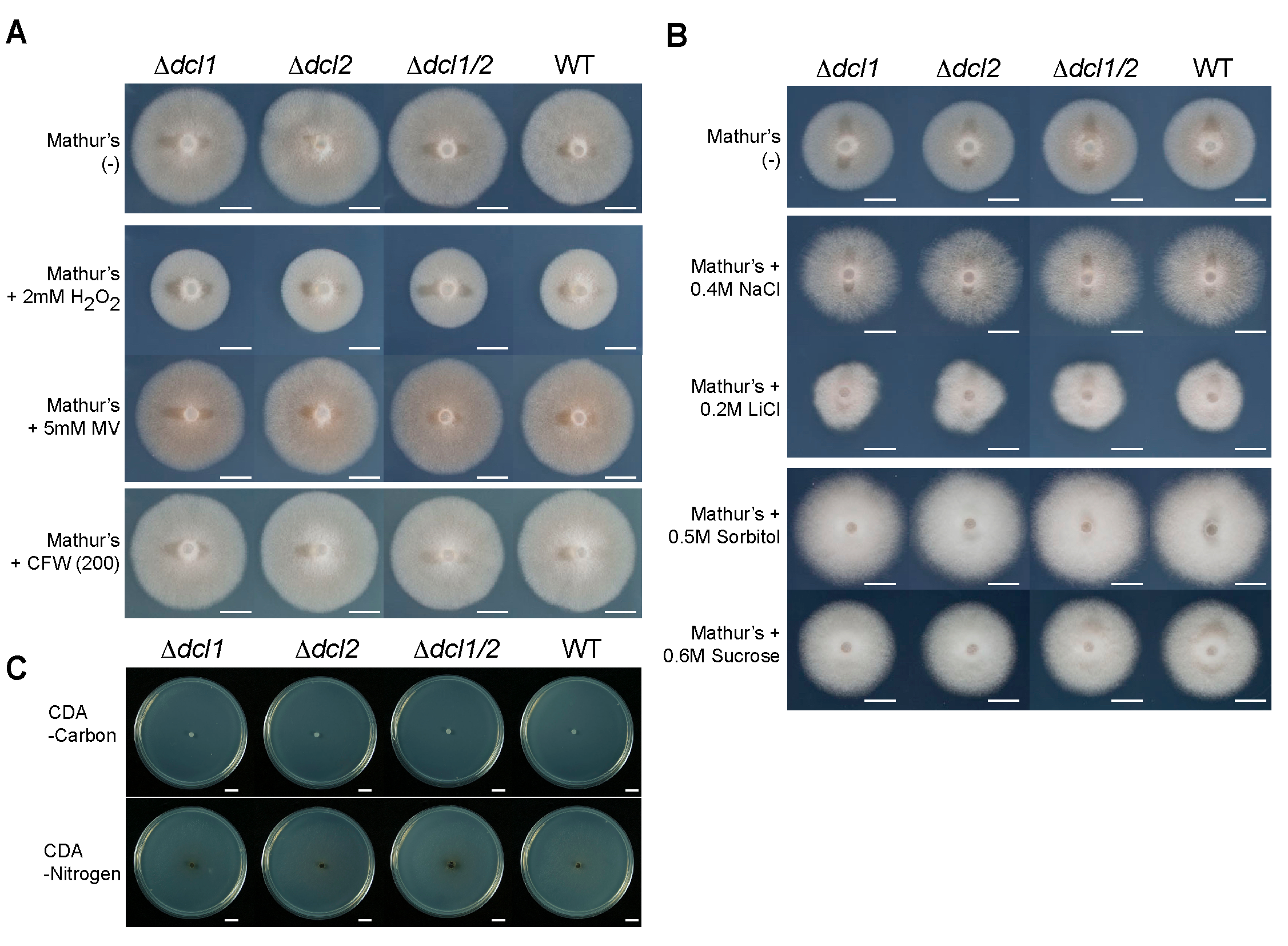

Supplement: S7 Fig — Colony morphology of Δdcl1, Δdcl2, Δdcl1Δdcl2 and wild-type (WT) under selected stress -related conditions. Cultures were grown at 25°C and dark conditions. Scale bar = 1 cm. (A) Strains were grown for four days on Mathur’s media alone (–) or supplemented with a stress component: 2 mM H2O2, 5 mM Methyl Viologen (MV), 200 mg/ml Calcofluor white (CFW). (B) Strains were grown for three days on Mathur’s media alone (–) or supplemented with an osmotic stress component: 0.4 M NaCl, 0.2 M LiCl, 0.5 M Sorbitol and 0.6 M Sucrose. (C) Strains were grown for seven days on CDA media without Carbon (CDA-C) or without Nitrogen (CDA-N). (TIF) [file ppat.1005640.s010.tif]

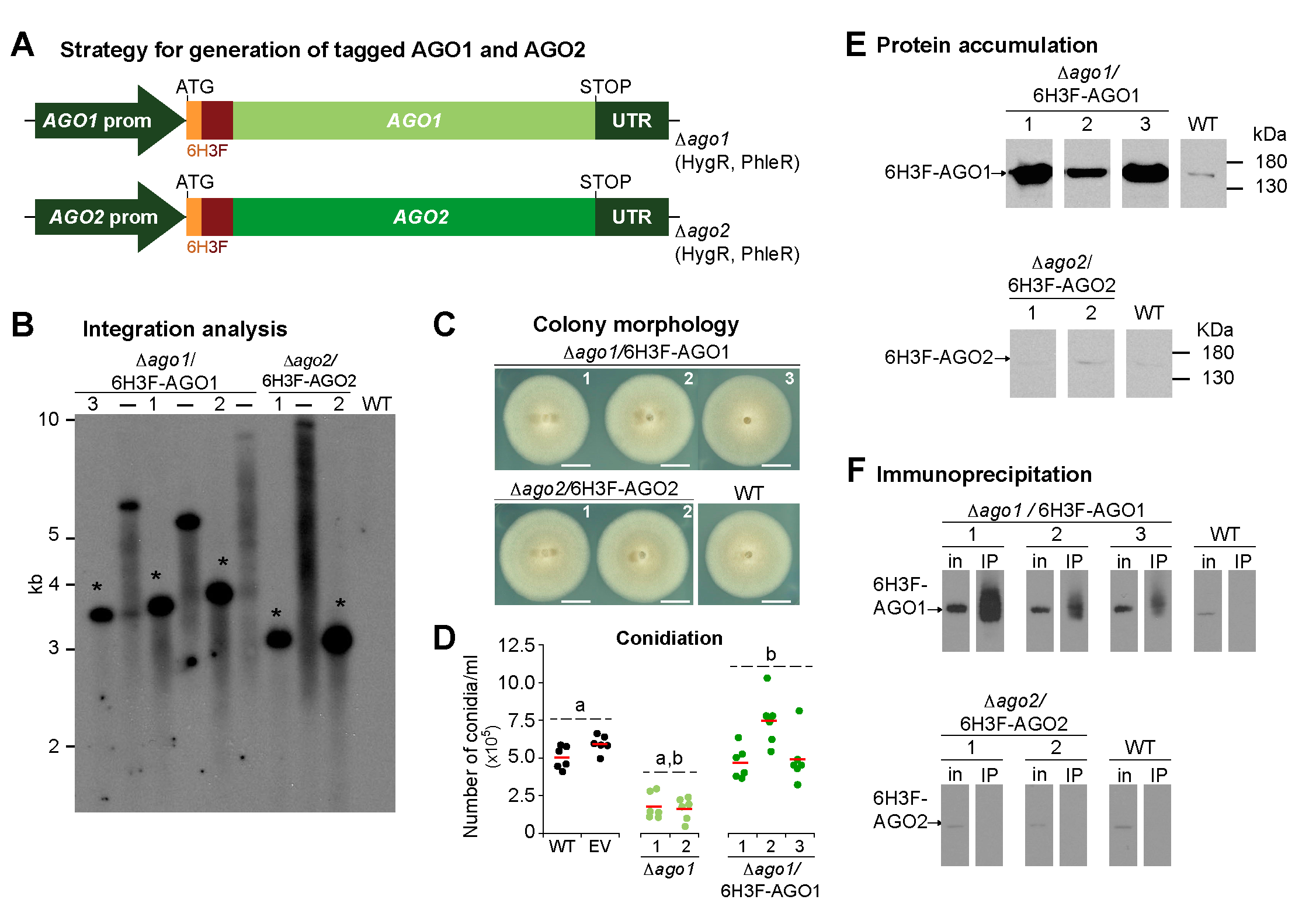

Supplement: S8 Fig — (A) Schematic diagram of the constructs. A 6His-3FLAG (6H3F) epitope was cloned in frame with AGO1 or AGO2 and expressed under the control of its own promoter (prom). C higginsianum Δago1 and Δago2 mutant strains expressing the Hygromycin resistance (HygR) were transformed with the corresponding tagged AGO1 and AGO2 constructs harboring the Phleomycin resistance (PhleR). UTR, Untranslated region. (B) Analysis of integration by Southern blot using the Phleomycin probe. Only those strains showing a single hybridization pattern with HindIII-digested genomic DNA, indicative of a single copy integration event, were selected for further analysis (indicated with an asterisks). Numbers above the blot refer to the ID of the three AGO1 and two AGO2 independent transformants selected. (C) Colony morphology after 3 days of growth for the selected C. higginsianum Δago1/6H3F-AGO1 (three independent transformants), Δago2/6H3F-AGO2 (two independent transformants), and control (wild-type) strains. Scale bar = 1 cm (D) Conidia production in controls strains (wild-type and empty vector), Δago1 mutant strains, and Δago1/6H3F-AGO1 strains. Conidia were collected after 7 days of growth in Mathur’s medium and counted with a hemocytomer. Significantly different pairwise comparisons are indicated by shared lowercase letters (p < 0.05). (E) Immunoblots of protein extracts from Δago1/6H3F-AGO1 (upper panel) and Δago2/6H3F-AGO2 (lower panel) with a wild-type (WT) control. (F) Immunoprecipitation of C. higginsianum tagged-AGOs. Immunoblots of protein extracts from input (in) and immunoprecipitated (IP) samples from Δago1/6H3F-AGO1 (upper panel) and Δago2/6H3F-AGO2 (lower panel) with a wild-type (WT) control. 6H3F-AGO2 was not detected in the IP samples, even with longer exposures (data not shown). (TIF) [file ppat.1005640.s011.tif]

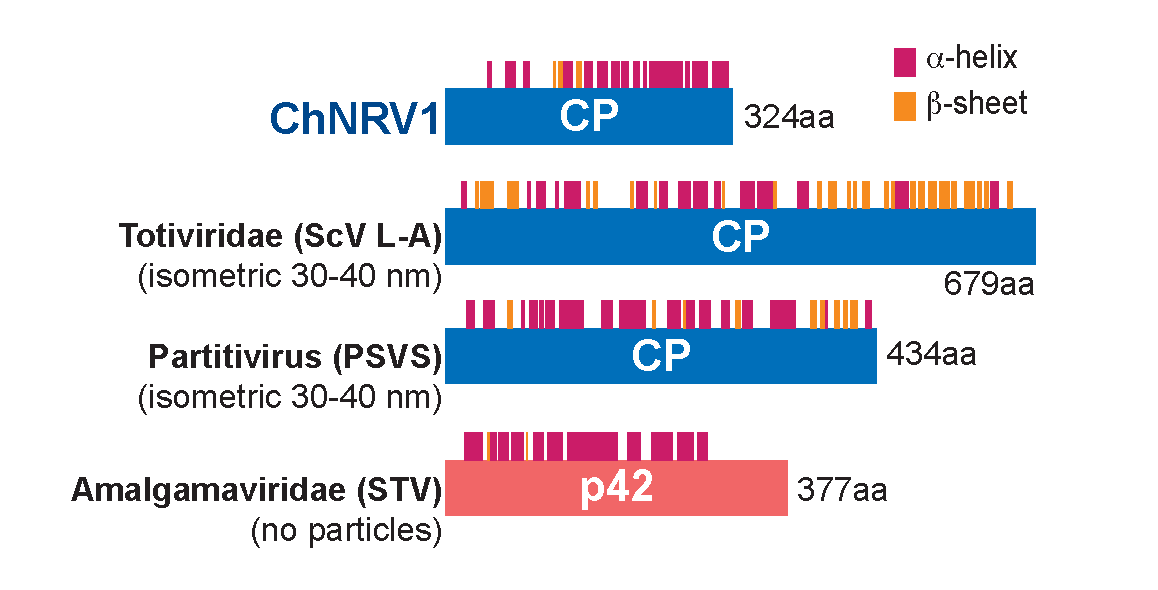

Supplement: S9 Fig — Capsid secondary structures α-helix and ß-sheets are shown in pink and orange, respectively. Colletotrichum higginsianum Non-segmented dsRNA virus 1 (ChNRV1), Saccharomyces cerevisiae virus L-A (ScV-L-A), Penicillium stoloniferum virus S (PsV-S), Southern tomato virus (STV). aa, amino acids. (TIF) [file ppat.1005640.s012.tif]

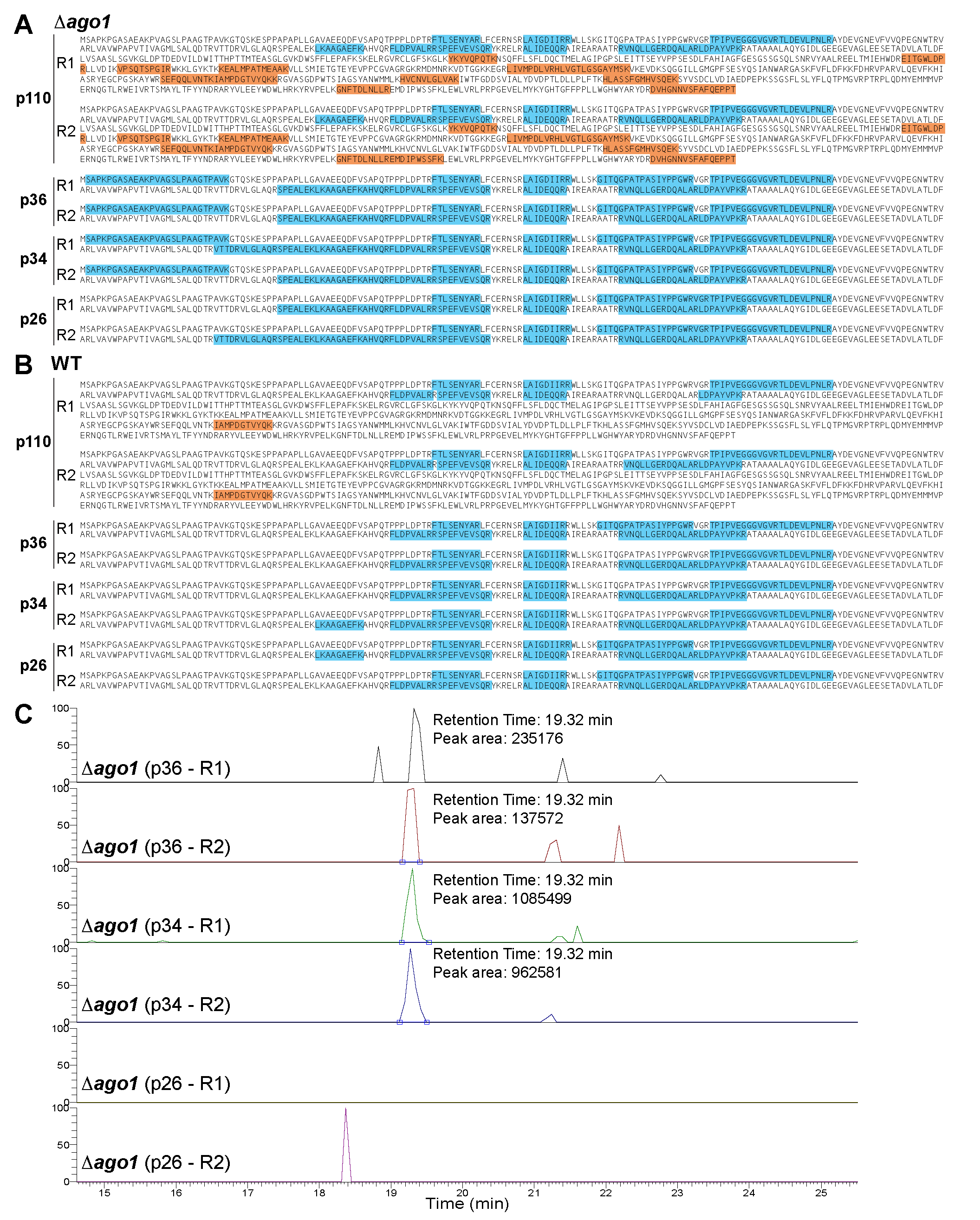

Supplement: S10 Fig — (A-B) Protein sequence coverage identified for ChNRV1 in samples p110, p36, p34, and p26 in both replicates (R1 and R2) in the Δago1 and wild-type (WT) samples. Highlighted in blue are the peptides matching to the capsid protein and in orange to RdRP protein in the Δago1 mutant (A) and wild-type (WT) (B) strains. (C) Extracted ion chromatograms (XIC) of the selected N-terminal peptide (detected at 810.78 m/z, z = 3) from p36, p34, and p26 samples from the Δago1 mutant strain. Retention time and integrated peak area are indicated next to the peak. (TIF) [file ppat.1005640.s013.tif]

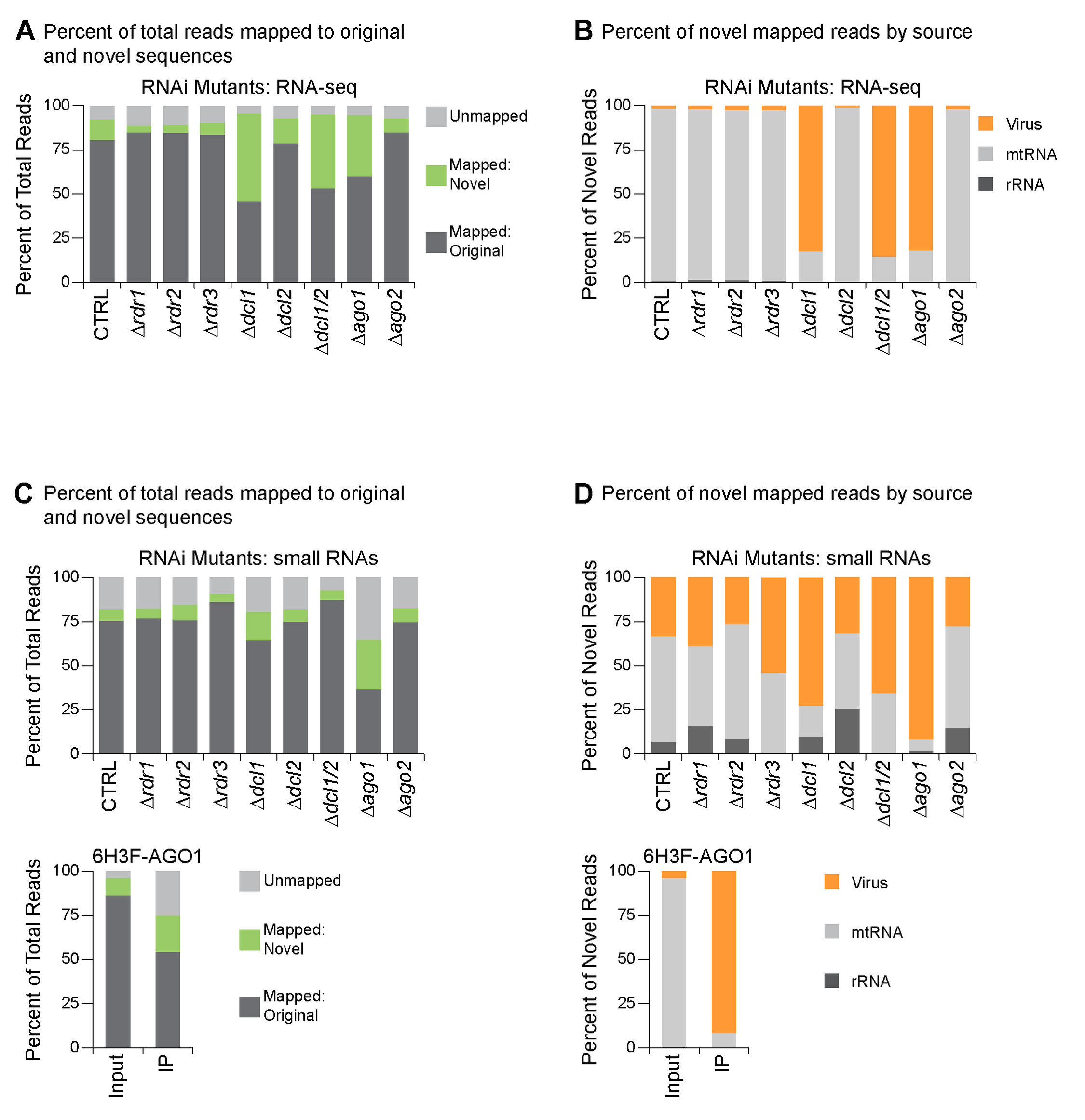

Supplement: S11 Fig — (A) Percent of total RNA-seq reads by category: mapped to original reference genome, mapped to new sequences, or remain unmapped. (B) Breakdown of the source of newly mapping RNA-seq reads. (C) Percent of total small RNA reads by category: mapped to original reference genome, mapped to new sequences, or remain unmapped. (D) Breakdown of the source of the newly mapping small RNA reads. (TIF) [file ppat.1005640.s014.tif]

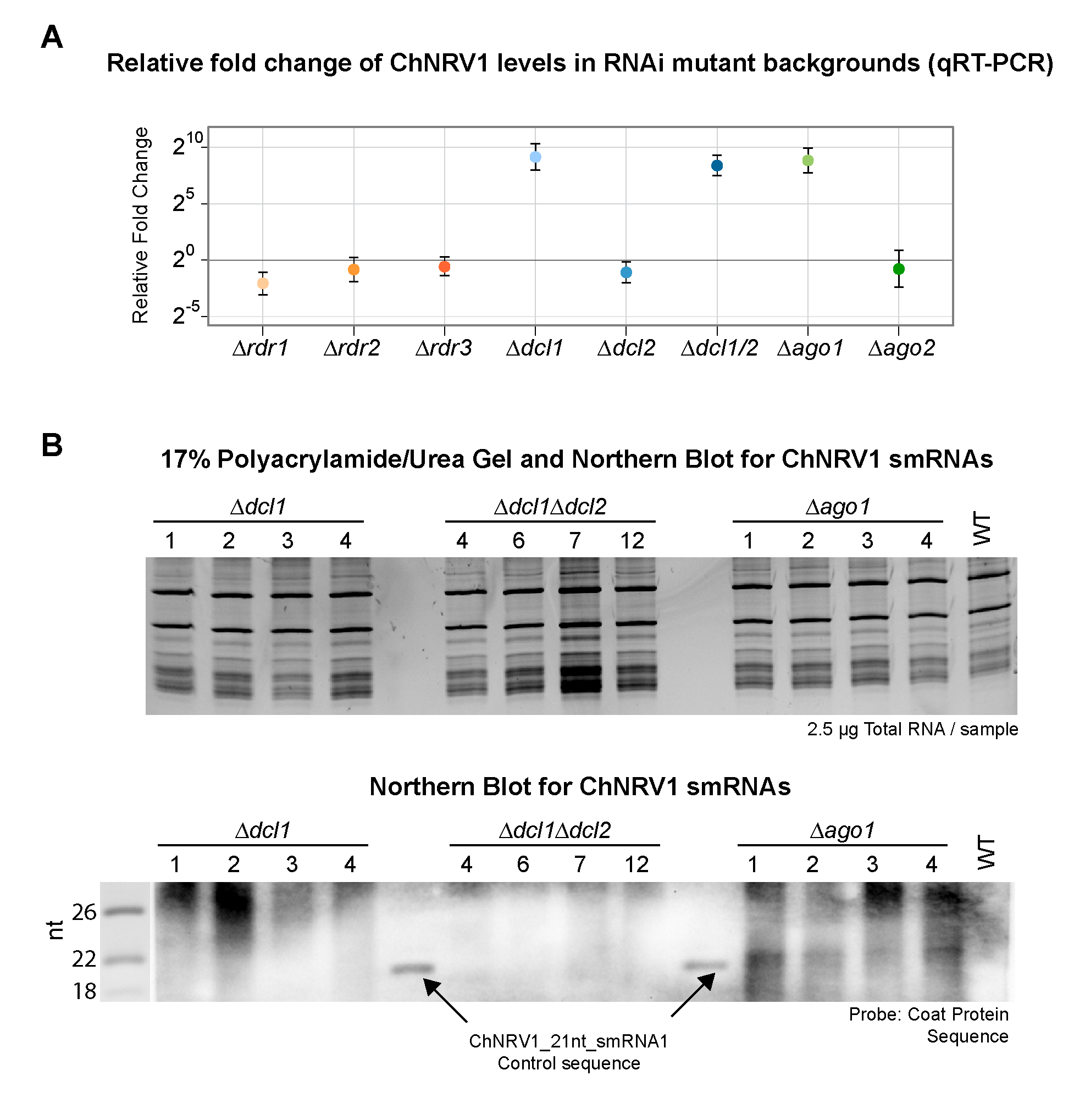

Supplement: S12 Fig — (A) qRT-PCR analysis of ChNRV1 levels in mutant and wild-type backgrounds using primers for the RDRP sequence. Mean fold change (2^-ΔΔCt) relative to ChNRV1 levels in wild-type (+/- standard deviation) is plotted. Four biological replicates were averaged and ACTIN and TUBULIN genes were used for normalization. (B) Small RNAs in total RNA were separated using a 17% polyacrylamide/urea gel, followed by transfer to a membrane and probed with a DIG-labeled probe generated from the coat protein sequence. Top panel is the ethidium bromide stain gel demonstrating equal loading and intactness of RNAs greater than 30 nt for each replicate. Lower panel is the blot of lower region of the gel. A 21 nt known sequence from the ChNRV1 coat protein sequence was included as a control for size and probe-specificity. A diffuse signal is visible in the Δdcl1 replicates, while no signal is apparent in either Δdcl1Δdcl2 or the WT sample. The Δago1 lanes show a band at 22 nt in each replicate. (TIF) [file ppat.1005640.s015.tif]

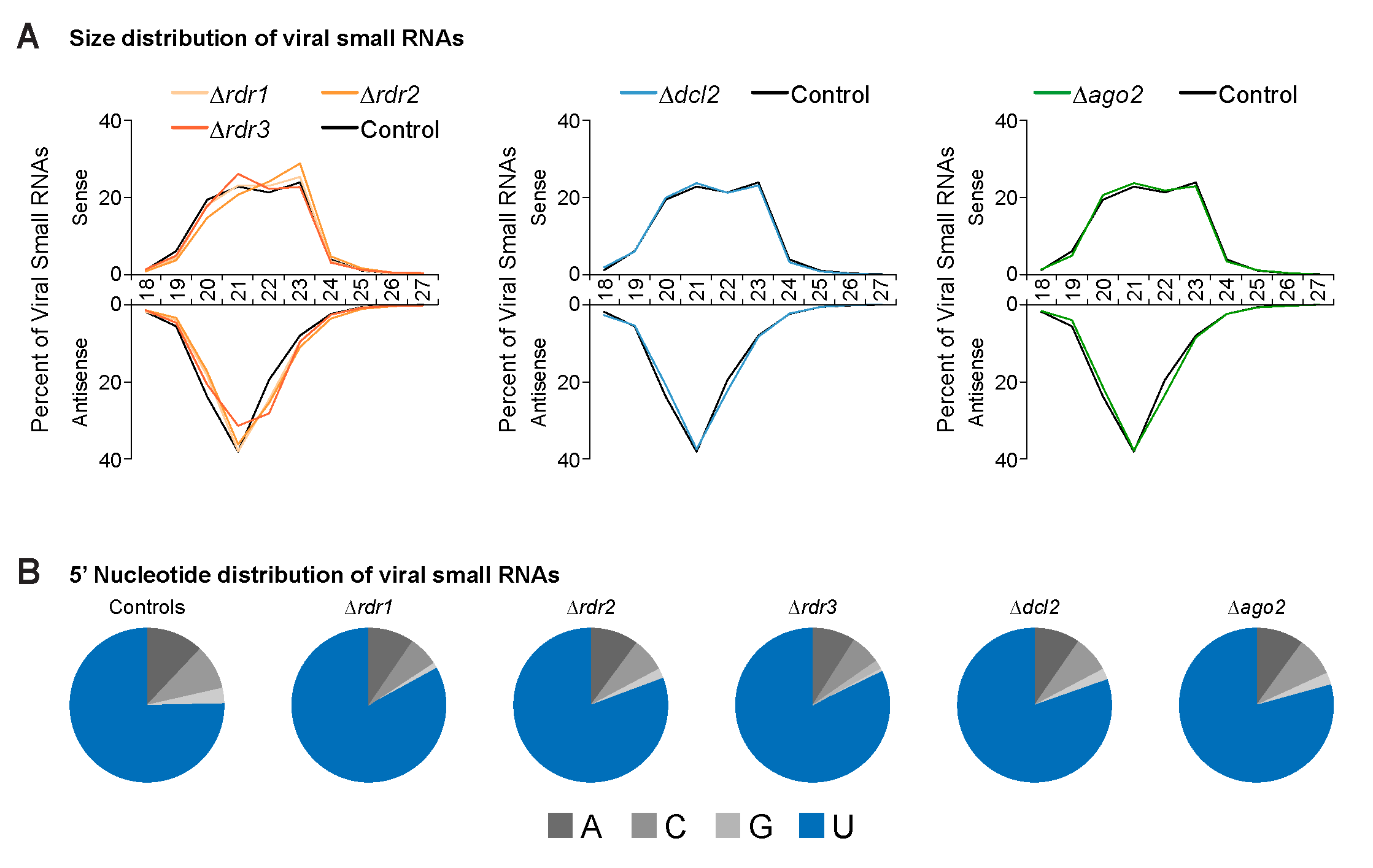

Supplement: S13 Fig — (A) Read size distribution as a percentage of total viral small RNAs by strand for Δrdr1, Δrdr2, Δrdr3, Δago2, and Δdcl2. The distribution of the control replicates, found also in Fig 8D, is included in each panel for reference. (B) 5’ nucleotide distribution for viral small RNAs from Δrdr1, Δrdr2, Δrdr3, Δago2, and Δdcl2. 5’ nucleotide distribution for control replicates, found also in Fig 8E, is included for reference. (TIF) [file ppat.1005640.s016.tif]

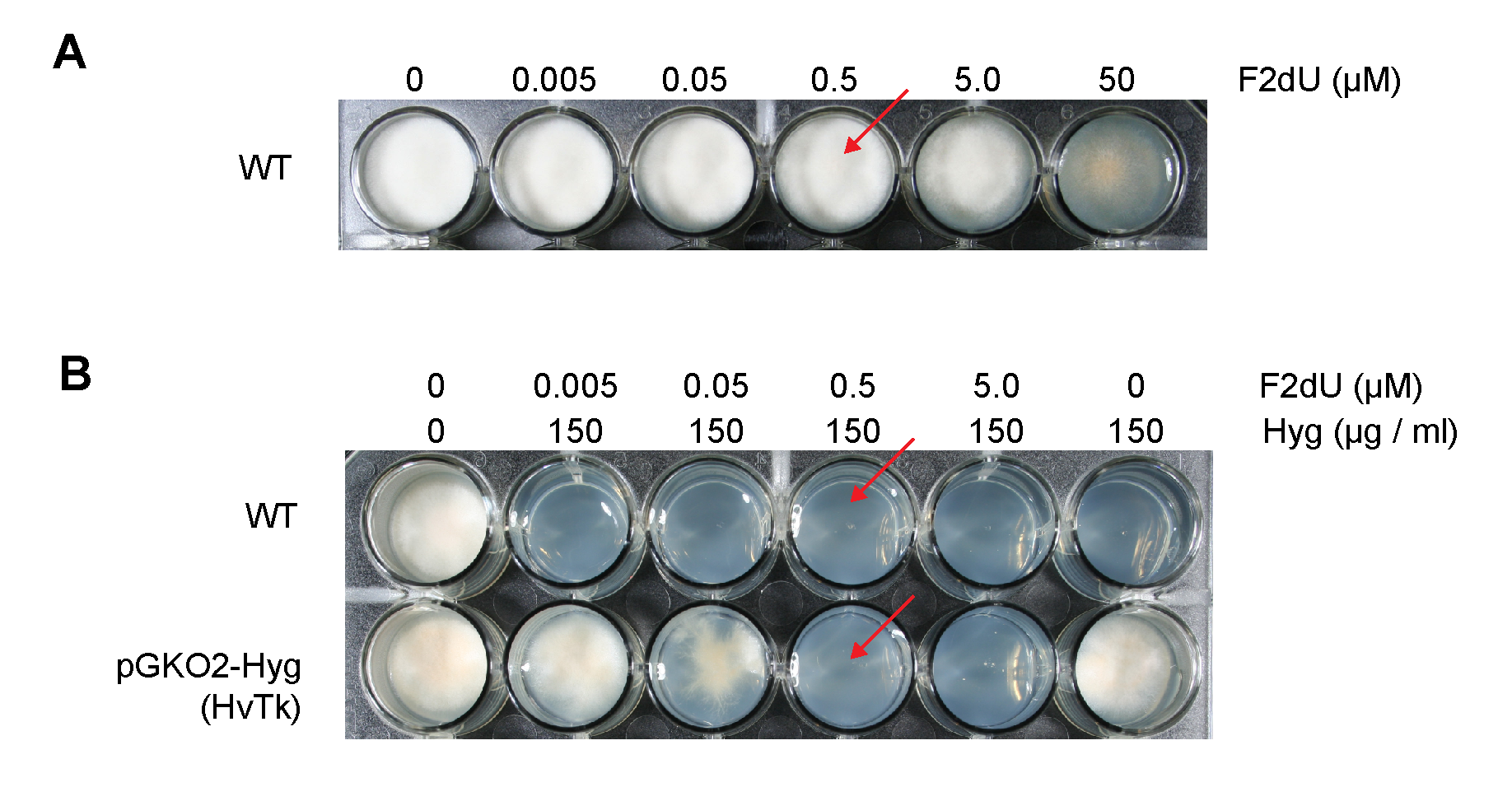

Supplement: S14 Fig — (A) Growth of C. higginsianum conidia in the presence of 5-fluoro-2’-deoxyuridine (F2dU) at concentrations ranging from 0.005–50 mM. Only the highest concentration of F2dU (50 μM) had a visible, negative, effect on C. higginsianum growth. (B) Growth of C. higginsianum conidia un-transformed (WT) and transformed with a vector containing the Hvtk and Hyg genes (pGKO2-Hyg) in the presence of Hygromycin (150 μg/mL) and F2dU at a range of concentration not toxic for C. higginsianum conidia (0.005–5 μM) as determined in (A). An optimal concentration of 150 μg/mL Hygromycin and 0.5 μM of F2dU was determined to guarantee an optimal growth of homologous recombinants (no presence Hvtk gene) and death for ectopic transformant (presence Hvtk gene). Hyg, Hygromycin. Hvtk, herpes Virus thymidine kinase. (TIF) [file ppat.1005640.s017.tif]
