# Supplementary material for: Small RNA-Based Antiviral Defense in the Phytopathogenic Fungus Colletotrichum higginsianum
Source: PLoS Pathog. 2016 Jun 2;12(6):e1005640. doi: 10.1371/journal.ppat.1005640 (PMC4890784; doi:10.1371/journal.ppat.1005640)
Supplement: S1 Table — (DOCX) [file ppat.1005640.s018.docx]

**S1 Table. Accession numbers used for generating the phylogenetic tree for the RDR, DCL and AGO in the Ascomycota clade.**

| **Genus / Species (strain)** | **RNA dependent RNA polymerase (RDR)** | **Dicer**  **(DCL)** | **Argonaute**  **(AGO)** |
| --- | --- | --- | --- |
| **Sordariomycetes** | | | |
| *Colletotrichum higginsianum* (IMI 349063) | ChRDR1: CH063_02767 | ChDCL1: CH063_06582 | ChAGO1: CH063_04066 |
|  | ChRDR2: CH063_05776 | ChDCL2: CH063_02619 | ChAGO2: CH063_09722 |
|  | ChRDR3: CH063_08349 | - | - |
| *Colletotrichum graminicola* (M1.001) | CG1: GLRG_09527 | CG1: GLRG_08364 | CG1: GLRG_05439 |
|  | CG2: GLRG_02515 | CG2: GLRG_05246 | CG2: GLRG_06683 |
|  | CG3: GLRG_06276 | - | - |
| *Cryphonectria parasitica* (EP155) | CP (RDR1): CCV01471 | CP (DCL1): Q2VF19 | AGL1: ACY36939 |
|  | CP (RDR2): CCV01472 | CP (DCL2): Q2VF18 | AGL2: ACY36940 |
|  | CP (RDR3): CCV01473 | - | AGL3: ACY36941 |
|  | - | - | AGL4: ACY36942 |
| *Magnaporthe oryzae* (70-15) | MO1: MGG_13453 | MO (MDL-1): MGG_01541 | MO1: MGG_01294 |
|  | MO2: MGG_02748 | MO (MDL-2): MGG_12357 | MO2: MGG_13617 |
|  | MO3: MGG_06205 | - | MO3: MGG_14873 |
| *Neurospora crassa* (OR74A) | NC (SAD-1): NCU02178 | NC (SMS-3): NCU08270 | NC (QDE-2): NCU04730 |
|  | NC (QDE-1): NCU07534 | NC (DCL-2): NCU06766 | NC (SMS-2): NCU09434 |
|  | NC (RRP3): NCU08435 | - | - |
| *Trichoderma atroviride* (IMI 206040) | TA (RDR1): TRIATDRAFT_321718 | TA (DCR1): TRIATDRAFT_292263 | TA (AGO1) TRIATDRAFT_245602 |
|  | TA (RDR2): TRIATDRAFT_225118 | TA (DCR2): TRIATDRAFT_291296 | TA (AGO2): TRIATDRAFT_20708 |
|  | TA (RDR3): TRIATDRAFT_317554 | - | TA (AGO3): TRIATDRAFT_36522 |
|  | TA (RDR4): TRIATDRAFT_294361 | - |  |
| **Eurotiomycetes** | | | |
| *Aspergillus niger* (ATCC 1015) | AN1: ASPNIDRAFT_40039 | AN1: ASPNIDRAFT_211065 | AN1: ASPNIDRAFT_45684 |
|  | AN2: ASPNIDRAFT_179412 | AN2: ASPNIDRAFT_119009 | AN2: ASPNIDRAFT_55081 |
|  | AN3: ASPNIDRAFT_176277 | AN3: ASPNIDRAFT_42773 | AN3: CAL00657 |
| *Penicillium chrysogenum* (Wisconsin 54-1255) | PC1: Pc21g18410 | PC1: Pc12g13700 | PC1: Pc12g03410 |
|  | PC2: Pc20g02950 | PC2: Pc21g06890 | PC2: Pc22g03240 |
|  | - | - | PC3: Pc13g09230 |
| *Penicillium marneffei* (ATCC 18224) | PM1: PMAA_079530 | PM1: PMAA_092010 | PM1: PMAA_009480 |
|  | PM (SAD-1): PMAA_003080 | PM2: PMAA_030960 | PM2: PMAA_102710 |
|  | PM2: PMAA_093500 | - | PM3: PMAA_023530 |
| *Coccidioides immitis* (RS) | CI1: CIMG_06658 | CI1: CIMG_05433 | CI1: CIMG_08295 |
|  | CI2: CIMG_00395 | CI2: CIMG_13589 | CI2: CIMG_03053 |
|  | CI3: CIMG_02715 | - | CI3: CIMG_03797 |
| **Dothideomycetes** | | | |
| *Alternaria brassicicola* | AB1: AB01807 | AB1: AB06823 | AB1: AB02557 |
|  | AB2: AB08841 | AB2: AB00535 | AB2: AB02263 |
|  | AB3: AB01608 | - | - |
| *Cercospora zeae-maydis* | CM1: 23160 | CM1: 43176 | CM1: 56848 |
|  | CM2: 71603 | CM2: 109000 | - |
|  | CM3: 68227 | - | - |
| *Mycosphaerella graminicola* (IPO323) | MG1: MYCGRDRAFT_117718 | - | MG1: MYCGRDRAFT_38035 |
|  | MG2: MYCGRDRAFT_51407 | - | MG2: MYCGRDRAFT_90232 |
|  | MG3: MYCGRDRAFT_49833 | - | MG3: MYCGRDRAFT_10621 |
| *Pyrenophora tritici-repentis* (Pt-1C-BFP) | PT1: PTRG_10475 | PT1: PTRT_05720 | PT1: PTRG_08652 |
|  | PT2: PTRG_07015 | PT2: PTRT_08809 | PT2: PTRG_01413 |
|  | PT3: PTRG_06422 | - | PT3: PTRG_06348 |
| *Stagonospora nodorum* (SN15) | SN1: SNOG_01986 | SN1: SNOG_07542 | SN1: SNOG_06565 |
|  | SN2: SNOG_15784 | - | SN2: SNOG_10544 |
|  | SN3: SNOG_14810 | - | - |
| *Leptosphaeria maculans* (JN3) | LM1: LEMA_P088990 | LM1: Lema_T041660 | LM1: LEMA_P015760 |
|  | LM2: LEMA_P117350 | LM2: Lema_T036310 | LM2: LEMA_P079240 |
|  | LM3: LEMA_P095020 | - | LM3: LEMA_P097140 |
| **Leotiomycetes** | | | |
| *Botrytis cinerea* (BcT4) | BC1: BcT4_3181 | BC1: BcT4_9304 | BC1: BcT4_391 |
|  | BC2: BcT4_6211 | BC2: BcT4_9842 | BC2: BcT4_6575 |
|  | BC3: BcT4_3773 | - | BC3: BcT4_9701 |
|  | - | - | BC4: BcT4_9039 |
| *Sclerotinia borealis* (F-415) | SB1: SBOR_5459 | SB1: SBOR_2771 | SB1: SBOR_4936 |
|  | SB2: SBOR_2395 | SB2: SBOR_5784 | SB2: SBOR_6288 |
|  | SB3: SBOR_9633 | - | SB3: SBOR_7807 |
|  | - | - | SB4: SBOR_8346 |
| **Outgroups** | | | |
| *Arabidopsis thaliana* (Col-0) | AtRDR6: AF239718 | AtDCL1: Q9SP32 | AtAGO1: U91995 |
