# Supplementary material for: Small RNA-Based Antiviral Defense in the Phytopathogenic Fungus Colletotrichum higginsianum
Source: PLoS Pathog. 2016 Jun 2;12(6):e1005640. doi: 10.1371/journal.ppat.1005640 (PMC4890784; doi:10.1371/journal.ppat.1005640)
Supplement: S2 Table — (DOCX) [file ppat.1005640.s019.docx]

**S2 Table. Summary of RNA-seq reads sequenced from RNAi mutants and controls.**

| **Code** | **Description** | **Total Processed Reads^a^** | **Total Reads Mapped: Original Genome^b^** | **Total Reads Mapped: Modified Genome^c^** |
| --- | --- | --- | --- | --- |
| Controls | *C. higginsianum* IMI 349063 (WT) | 43,731,034 | 35,488,459 | 40,543,944 |
|  | WT + empty vector pGKO2 |  |  |  |
| ∆*rdr1* | Knockout mutant of ChRDR1 (CH063_02767) | 60,632,175 | 51,710,187 | 53,972,455 |
| ∆*rdr2* | Knockout mutant of ChRDR2 (CH063_05776) | 68,595,964 | 58,000,198 | 61,115,941 |
| ∆*rdr3* | Knockout mutant of ChRDR3 (CH063_08349) | 47,394,521 | 39,591,637 | 42,713,666 |
| ∆*dcl1* | Knockout mutant of ChDCL1 (CH063_06582) | 38,611,421 | 17,785,957 | 36,974,787 |
| ∆*dcl2* | Knockout mutant of ChDCL2 (CH063_02619) | 37,627,838 | 29,693,147 | 35,035,070 |
| ∆*dcl1*∆*dcl2* | Double knockout mutant of ChDCL1 and ChDCL2 | 58,169,356 | 31,194,205 | 55,342,264 |
| ∆*ago*1 | Knockout mutant of ChAGO1 (CH063_04066) | 51,879,794 | 31,183,083 | 49,202,055 |
| ∆*ago*2 | Knockout mutant of ChAGO2 (CH063_09722) | 63,916,831 | 54,337,695 | 59,505,245 |

^a^Sum of all replicates: single replicate for WT and three for plasmid (controls); three replicates for ∆*rdr3* and four replicates for all other RNAi mutant genotypes.

^b^Reads were mapped to the version 1 *C. higginsianum* reference sequence available at <http://www.broadinstitute.org/annotation/genome/colletotrichum_group>

^c^Reads were mapped to *C. higginsianum* reference sequence plus: ChNRV1 sequence, mtRNA contig, rRNA contig.
