# Supplementary material for: Small RNA-Based Antiviral Defense in the Phytopathogenic Fungus Colletotrichum higginsianum
Source: PLoS Pathog. 2016 Jun 2;12(6):e1005640. doi: 10.1371/journal.ppat.1005640 (PMC4890784; doi:10.1371/journal.ppat.1005640)
Supplement: S3 Table — (DOCX) [file ppat.1005640.s020.docx]

**S3 Table. Summary of small RNAs sequenced from RNAi mutant and AGO immunoprecipitation libraries.**

| **Code** | **Description** | **Total Processed Reads^ab^** | **Total Processed Sequences^ab^** | **Total**  **Reads Mapped: Original^c^** | **Total Sequences Mapped: Original^c^** | **Total**  **Reads Mapped: Modified^d^** | **Total Sequences Mapped: Modified^d^** |
| --- | --- | --- | --- | --- | --- | --- | --- |
| Controls | *C. higginsianum* IMI 349063 (WT) | 80,742,350 | 4,363,358 | 59,186,159 | 2,349,943 | 66,262,723 | 2,464,003 |
|  | WT + empty vector pGKO2 |  |  |  |  |  |  |
| ∆*rdr1* | Knockout mutant of ChRDR1 (CH063_02767) | 59,355,513 | 3,708,670 | 46,053,670 | 1,906,749 | 49,312,920 | 2,000,232 |
| ∆*rdr2* | Knockout mutant of ChRDR2 (CH063_05776) | 66,801,510 | 3,936,432 | 50,808,121 | 2,016,000 | 56,473,742 | 2,126,886 |
| ∆*rdr3* | Knockout mutant of ChRDR3 (CH063_08349) | 66,944,572 | 4,107,687 | 57,617,610 | 2,751,487 | 60,634,359 | 2,859,905 |
| ∆*dcl1* | Knockout mutant of ChDCL1 (CH063_06582) | 62,329,967 | 2,898,306 | 40,603,161 | 1,118,808 | 50,596,285 | 1,263,994 |
| ∆*dcl2* | Knockout mutant of ChDCL2 (CH063_02619) | 78,289,539 | 5,849,636 | 58,840,454 | 2,988,101 | 64,529,646 | 3,111,528 |
| ∆*dcl1*∆*dcl2* | Double knockout mutant of ChDCL1 and ChDCL2 | 67,369,900 | 3,004,423 | 58,932,547 | 2,048,416 | 62,505,062 | 2,175,581 |
| ∆*ago*1 | Knockout mutant of ChAGO1 (CH063_04066) | 79,995,045 | 3,466,395 | 29,211,104 | 1,370,461 | 52,077,290 | 1,528,018 |
| ∆*ago*2 | Knockout mutant of ChAGO2 (CH063_09722) | 75,710,973 | 4,143,232 | 56,544,326 | 2,131,361 | 62,616,053 | 2,245,084 |
| AGO1input | ∆*ago1/*6His3FLAG-AGO1 | 39,935,814 | 4,109,636 | 35,864,268 | 3,378,320 | 38,381,849 | 3,456,747 |
| AGO1 IP | ∆*ago1/*6His3FLAG-AGO1 | 38,779,948 | 3,036,745 | 21,127,825 | 1,763,263 | 29,119,542 | 1,819,890 |
| AGO2input | ∆*ago2/*6His3FLAG-AGO2 | 29,504,944 | 3,275,962 | 26,473,287 | 2,705,324 | 28,325,849 | 2,760,732 |
| AGO2 IP | ∆*ago2/*6His3FLAG-AGO2 | 22,798,286 | 1,011,418 | 21,122,633 | 771,299 | 22,212,561 | 792,669 |
| WT input | Wild-type *C. higginsianum;* without tagged AGO | 15,568,751 | 1,712,429 | 13,693,910 | 1,423,153 | 14,973,415 | 1,454,356 |
| WT IP | Wild-type *C. higginsianum;* without tagged AGO | 9,914,171 | 514,515 | 9,212,923 | 397,966 | 9,685,739 | 409,363 |

^a^Sum of all replicates: two replicates for WT and plasmid; four replicates for all RNAi mutant genotypes. Three replicates for ∆*ago1*/6His3FLAG-AGO1, two replicates for ∆*ago2/*6His3FLAG-AGO2, one replicate of WT (mock IP).

^b^Reads containing ambiguous nucleotide(s) and less than 18 nt in length have been removed.

^c^Reads were mapped to the original reference sequence available at <http://www.broadinstitute.org/annotation/genome/colletotrichum_group>.

^d^Reads were mapped to the original genome sequence plus: ChNRV1 sequence, mtRNA contig, rRNA contig.
