# Supplementary material for: Small RNA-Based Antiviral Defense in the Phytopathogenic Fungus Colletotrichum higginsianum
Source: PLoS Pathog. 2016 Jun 2;12(6):e1005640. doi: 10.1371/journal.ppat.1005640 (PMC4890784; doi:10.1371/journal.ppat.1005640)
Supplement: S4 Table — (DOCX) [file ppat.1005640.s021.docx]

**S4 Table. Accession numbers and geographical accessions for the *Colletotrichum destructivum/higginsianum* strains screened for ChNRV1 by RT-PCR.**

| **Accession** | **Species** | **Origin** | **Host** | **ChNRV1**  **RT-PCR Amplification** |
| --- | --- | --- | --- | --- |
| MAFF305635 | *Colletotrichum destructivum* | Japan (Tokyo) | *Brassica rapa* L. Perviridis Group (Spinach mustard, Tendergreen mustard) | – |
| MAFF238563 | *Colletotrichum destructivum* | Japan (Chiba) | *Matthiola incana* (L.) R.Br.  (Common stock) | – |
| MAFF239947 | *Colletotrichum destructivum* | Japan (Shizuoka) | *Antirrhinum majus* L.  (Common snapdragon) | – |
| MAFF305968 | *Colletotrichum destructivum* | Japan (Okinawa) | *Brassica rapa* L. *Chinensis* Group  (Pak-Choi) | – |
| MAFF305970 | *Colletotrichum destructivum* | Japan (Okinawa) | *Brassica rapa* L. *Pekinensis* Group (Chinese cabbage) | – |
| IMI 349063A | *Colletotrichum higginsianum* | Trinidad and Tobago | *Brassica rapa* L. *Chinensis* Group  (Pak-Choi) | + |
| IMI 349061 | *Colletotrichum higginsianum* | Trinidad and Tobago | *Brassica rapa* L. *Chinensis* Group  (Pak-Choi) | + |
| IMI 391904 | *Colletotrichum higginsianum* | Tunisia | *Raphanus raphanistrum* | – |

Presence (+) or Absence (-) of *ChNRV1* transcripts in the *C. higginsianum* wild-type strain IMI 349063A as determined by semi-quantitative RT-PCR analysis, using primers spanning both ORFs (S7 Table). Control reactions were done with *ACTIN genes*

MAFF (<http://www.gene.affrc.go.jp/about-micro_en.php>)

IMI (http://www.cabi.org/services/microbial-services/culture-collection/)
