# Supplementary material for: Small RNA-Based Antiviral Defense in the Phytopathogenic Fungus Colletotrichum higginsianum
Source: PLoS Pathog. 2016 Jun 2;12(6):e1005640. doi: 10.1371/journal.ppat.1005640 (PMC4890784; doi:10.1371/journal.ppat.1005640)
Supplement: S5 Table — (DOCX) [file ppat.1005640.s022.docx]

**S5 Table. Identification of ChNRV1 proteins in *C. higginsianum*** Δ***ago1* and wild type strains following 1-D SDS-PAGE and Mass Spectrometry.**

| Strain | Sample ID | Replicate | Spectral Counts | IDs | % IDs | Total Proteins Identified | Spectral Counts | | Normalized Counts | | % Coverage | | Unique peptides | |
| --- | --- | --- | --- | --- | --- | --- | --- | --- | --- | --- | --- | --- | --- | --- |
|  |  |  |  |  |  |  | CP | RdRp | CP | RdRp | CP | RdRp | CP | RdRp |
| ∆*ago1* | p110 | R1 | 4584 | 817 | 18 | 126 | 94 (1) | 84 (2) | 94 (1) | 84 (2) | 31 | 24 | 14 | 14 |
|  |  | R2 | 4020 | 634 | 16 | 119 | 46 (2) | 47 (1) | 46 (2) | 47 (1) | 31 | 23 | 13 | 13 |
|  | p36 | R1 | 6466 | 1650 | 26 | 240 | 873 (1) | 7 (33) | 820 (1) | 7 (33) | 49 | 14 | 22 | 7 |
|  |  | R2 | 5900 | 1663 | 28 | 266 | 867 (1) | 11 (13) | 807 (1) | 10 (9) | 40 | 15 | 21 | 8 |
|  | p34 | R1 | 6283 | 1568 | 25 | 284 | 727 (1) | 12 (12) | 719 (1) | 12 (10) | 52 | 16 | 22 | 8 |
|  |  | R2 | 6662 | 1709 | 26 | 279 | 855 (1) | 18 (4) | 775 (1) | 16 (4) | 49 | 17 | 21 | 9 |
|  | p26 | R1 | 8116 | 1799 | 22 | 242 | 1056 (1) | 6 (37) | 910 (1) | 5 (34) | 41 | 11 | 22 | 6 |
|  |  | R2 | 7235 | 1653 | 23 | 260 | 892 (1) | 6 (34) | 835 (1) | 6 (31) | 45 | 11 | 22 | 6 |
| WT | p110 | R1 | 3276 | 262 | 8 | 73 | 10 | 1 | 10 | 1 | 23 | 1.90 | 9 | 1 |
|  |  | R2 | 3528 | 439 | 12 | 117 | 11 | 1 | 11 | 1 | 27 | 1.90 | 10 | 1 |
|  | p36 | R1 | 4641 | 1687 | 36 | 341 | 18 | - | 16 | - | 33 | - | 14 | - |
|  |  | R2 | 4605 | 1256 | 27 | 345 | 30 | - | 37 | - | 28 | - | 14 | - |
|  | p34 | R1 | 4467 | 1404 | 31 | 352 | 19 | - | 21 | - | 28 | - | 14 | - |
|  |  | R2 | 4408 | 1271 | 29 | 338 | 17 | 3 | 21 | 4 | 31 | 5.40 | 13 | 3 |
|  | p26 | R1 | 5674 | 1523 | 27 | 366 | 33 | - | 33 | - | 36 | - | 17 | - |
|  |  | R2 | 4902 | 1372 | 28 | 370 | 33 | - | 26 | - | 33 | - | 13 | - |

In ∆*ago1*, number in parenthesis is the ranking of CP and RdRP with respect to the other proteins identified. ID, total of spectral counts identified; % ID, percentage of spectral counts identified
