# Supplementary material for: Small RNA-Based Antiviral Defense in the Phytopathogenic Fungus Colletotrichum higginsianum
Source: PLoS Pathog. 2016 Jun 2;12(6):e1005640. doi: 10.1371/journal.ppat.1005640 (PMC4890784; doi:10.1371/journal.ppat.1005640)
Supplement: S6 Table — (DOCX) [file ppat.1005640.s023.docx]

**S6 Table. Accession numbers used for generating the phylogenetic tree for the RDR from selected mycoviruses.**

| **Genus** | **Species (strain abbreviation)** | **RNA dependent RNA polymerase (RDRP)** |
| --- | --- | --- |
| *Totiviridae* | *Scheffersomyces segobiensis* virus L (SSVL) | AGG68771 |
|  | *Xanthophyllomyces dendrorhous* virus L1b (XDV1b) | AFH09414 |
|  | *Saccharomyces cerevisiae* virus L-A-lus (SCVLA) | NP_620495 |
|  | *Black raspberry* virus F (BRVF) | YP_001497151 |
|  | *Ustilaginoidea virens* RNA virus 1 (UVV1) | AGO04407 |
|  | *Sphaeropsis sapinea* RNA virus 1 (SSV1) | NP_047558 |
|  | *Rosellinia necatrix* victorivirus 1 (RNV1) | YP_008130308 |
|  | *Helicobasidium mompa* totivirus 1-17 (HM17V) | BAC81754 |
|  | *Magnaporthe oryzae* virus 1 (MOV1) | BAD60833 |
| *Partitiviridae* | *Aspergillus fumigatus* partitivirus-1 (AFPV1) | CAY25801 |
|  | *Penicillium stoloniferum* virus S (PSVS) | AAN86834 |
|  | *Gremmeniella abietina* RNA virus MS1 (GAV-MS1) | AAM12240 |
|  | *Ophiostoma* partitivirus 1 (OPV1) | CAJ31886 |
|  | *Botryotinia fuckeliana* partitivirus 1 (BFPV1) | CAM33266 |
|  | *Discula destructiva* virus 1 (DDV1) | AAG59816 |
|  | *Discula destructiva* virus 2 (DDV2) | AAK59379 |
|  | *Ustilaginoidea virens* partitivirus (UVPV) | AGO04402 |
|  | *Verticillium dahliae* partitivirus 1 (VDPV1) | AGI52210 |
| *Amalgamaviridae* | *Zygosaccharomyces bailii* virus Z | NP_624325 |
|  | *Alternaria longipes* dsRNA virus 1 (ALRV1*)* | YP_009052469 |
|  | *Colletotrichum higginsianum* Non-segmented dsRNA Virus 1 (ChNRV1) | AIW81424 |
|  | *Beauveria bassiana* RNA virus 1 (BBV1-A24) | AKC57301 |
|  | *Beauveria_bassiana*_RNA_virus_1 (BBNV-1) | YP_009154711 |
|  | *Penicillium_janczewskii_Beauveria*_bassiana-like_virus_1 (PJBBV1) | ALO50135 |
|  | *Ustilaginoidea_virens_unassigned_RNA_virus_HNND-1* | YP_009154709 |
| Unclassified dsRNA viruses | *Ustilaginoidea virens* RNA virus M | YP_009094186 |
|  | *Rhizoctonia solani* dsRNA virus 1 (RSV1) | AFZ85210 |
|  | *Fusarium graminearum* dsRNA mycovirus-4 (FGMV4) | YP_003288790 |
|  | *Curvularia thermal tolerance* virus *(CThTV)* | YP_001976145 |
|  |  | YP_001976146 |
|  | *Cryphonectria parasitica* bipartite mycovirus 1 (CPMV1) | YP_007985675 |
|  | Heterobasidion RNA virus 6 (HBV6) | AHA82557 |
| *Reoviridae* | Human rotavirus A (ROTHA) | ACR22792 |
