# Supplementary material for: Small RNA-Based Antiviral Defense in the Phytopathogenic Fungus Colletotrichum higginsianum
Source: PLoS Pathog. 2016 Jun 2;12(6):e1005640. doi: 10.1371/journal.ppat.1005640 (PMC4890784; doi:10.1371/journal.ppat.1005640)
Supplement: S7 Table — (DOCX) [file ppat.1005640.s024.docx]

**S7 Table. Primers used in this study**

| **Primer name** | **Sequence** |
| --- | --- |
| **Primers for generation of binary plasmids with Phleomycin resistance** | |
| *gdp.prom-HindIII-F* | CCAAGCTTGCGGTTCTATAGTGTCACCT |
| *cyc1.ter-SpeI-R* | GGACTAGTGGATCTCAACAGCGGTAAGA |
|  | |
| **Primers for deletion of *C. higginsianum* RNAi genes** | |
| *ChAGO1-TGR-F1* | CACCCCTATTGCATCCAAACGCCT |
| *ChAGO1-TGR-R1* | TTCCCTTTAGTGAGGGAAGCTTGGCCATTAGTTCATTCGACAGA |
| *ChAGO1-TGR-F2* | ACTAGTGGATCCCCCTCCTAGGCCCACTACCATGGTAGTTGGTT |
| *ChAGO1-TGR-R2* | AGCAATGGGTTCATGCAGCA |
| *ChAGO2-TGR-F1* | CACCCTCATATCCGACGGCCAGTT |
| *ChAGO2-TGR-R1* | TTCCCTTTAGTGAGGGAAGCTTGGCAGATGTTCAACATAACCTT |
| *ChAGO2-TGR-F2* | ACTAGTGGATCCCCCTCCTAGGCCCAATACTCCCAGGTCCTCGA |
| *ChAGO2-TGR-R2* | GTGTGAAGCAAATCCCTCGA |
| *ChDCL1-TGR-F1* | CACCCACACGCCATAGAGTCCAGA |
| *ChDCL1-TGR-R1* | TCCCTTTAGTGAGGGAAGCTTGGCAGCCAACCGCTTCTGCATA |
| *ChDCL1-TGR-R1(Phle)* | TCGAAAGATCGAGCTCAAGCTTGGCAGCCAACCGCTTCTGCATA |
| *ChDCL1-TGR-F2* | ACTAGTGGATCCCCCTCCTAGGCCCCAAACCATGCATCTCCTCA |
| *ChDCL1-TGR-F2 (Phle)* | ACCGCTGTTGAGATCCACTAGTCCCCAAACCATGCATCTCCTCA |
| *ChDCL1-TGR-R2* | CGAGAGTTTGTCGAGATGCT |
| *ChDCL2-TGR-F1* | CACCGCAGACATGAGGTATCTCCA |
| *ChDCL2-TGR-R1* | TCCCTTTAGTGAGGGAAGCTTGGCTCCGAGTCGATGGGATGAA |
| *ChDCL2-TGR-F2* | ACTAGTGGATCCCCCTCCTAGGCCGGGACATTCACGCAAGCTTA |
| *ChDCL2-TGR-R2* | CTGTTTACACGCAACACGGA |
| *ChRDR1-TGR-F1* | CACCCACAGAAGGACGAGTACGCA |
| *ChRDR1-TGR-R1* | TCCCTTTAGTGAGGGAAGCTTGGGCCGATCTCGAGCAGTGAAA |
| *ChRDR1-TGR-F2* | ACTAGTGGATCCCCCTCCTAGGCCGAAGGAGTACGAAGACGAGGA |
| *ChRDR1-TGR-R2* | CCATCGCGTGTGTGCCAAGT |
| *ChRDR2-TGR-F1* | CACCCACTGAACGACTCCAACTCT |
| *ChRDR2-TGR-R1* | TCCCTTTAGTGAGGGAAGCTTGGCCATCCGAGCCTATTGTCTT |
| *ChRDR2-TGR-F2* | ACTAGTGGATCCCCCTCCTAGGCCCTCATGCAGCGCAAGATCAA |
| *ChRDR2-TGR-R2* | CTGTAGCTGATGCAGAGGAT |
| *ChRDR3-TGR-F1* | CACCGACGGTGGCTAGTGGCTAGT |
| *ChRDR3-TGR-R1* | TCCCTTTAGTGAGGGAAGCTTGGGAAGCTGTCGCACAATCTCCT |
| *ChRDR3-TGR-F2* | ACTAGTGGATCCCCCTCCTAGGCCGGCTTTGCTATCACCTGAACA |
| *ChRDR3-TGR-R2* | CCTATGGCTCGTAACCATCGT |
| *hph-F* | CCAAGCTTCCCTCACTAAAGGGAACAAAAGCT |
| *hph-R* | GGCCTAGGAGGGGGATCCACTAGTTCTAGA |
| *Phle-F* | CCAAGCTTGAGCTCGATCTTTCGACACTGAAATACGT |
| *Phle-R* | GGACTAGTGGATCTCAACAGCGGTAAGA |
|  | |
| **Primers for generation of *C. higginsianum* tagged AGO strains** | |
| *AGO1-1F-ERV-F* | ccgatatcgattacaaggatgacgatgacaaggaggactatggacgtggaggccgtggt |
| *AGO1-Pst-R* | ccCTGCAGtggttgtaaactggctggttcgt |
| *AGO1.PROM-F* | caccggcgactatctgcggcttttgtt |
| *AGO1.PROM-6H2F-ERV/Pst-R* | cCTGCAGTTGATATCATGATCTTTATAATCACCGTCATGGTCTTTGTAGTCGTGATGGTGGTGGTGATGCATtggatggatgttagtccggtagg |
| *AGO2.prom-HindIII-F* | caccaagcttCCCTTGCCCTCGCAGCTGCTTGT |
| *AGO2.prom-EcoRI/AvrII-R* | ggcctagggaattcCATCTTGTAGATTGAATTGAAGATGAT |
| *AGO2-6H3F-EcoRI-F* | caccgaattcCATCACCACCACCATCACgactacaaagaccatgacggtgattataaagatcatgatatcgattacaaggatgacgatgacaagTCTTCCACCGGTCCTCCCCAGCAGA |
| *AGO2-AvrII-R* | ggcctaggCTCGACCCGTCCCAGACGTTA |
|  | |
| **Primers used for Southern blot hybridization:** | |
| *ChAGO1-probe-F* | ACACTCCCTCAATTCCTCAAC |
| *ChAGO1-probe-R* | CGTAAGTCTGTGGACTATCCTTC |
| *ChAGO2-probe-F* | GGTCGATCTTGATGAGGGAAAG |
| *ChAGO2-probe-R* | CGTCACGGAAGTAGATGATGTG |
| *ChDCL1-probe-F* | TCAGGGCAGAAGTAGAGAGAA |
| *ChDCL1-probe-R* | TCCAAGGGTGTTACCCAAAG |
| *ChDCL2-probe-F* | AACCCTCTCCGACTGACTTA |
| *ChDCL2-probe-R1* | CGGGAACTTGTGGAAGAGAAA |
| *ChDCL2-probe-R2* | GTTGACACTCTCATAGGCTCCA |
| *ChRDR1-probe-F* | CTTCCAGAGCCCACACTATTAAG |
| *ChRDR1-probe-R* | CCAACACGTGGTTTGGAGA |
| *ChRDR2-probe-F* | GGAGGATGCCAACAAGTTCT |
| *ChRDR2-probe-R* | CACAACCCGACTGATGTCTT |
| *ChRDR3-probe-F* | CTCGCTCTTCTCCGTGGTCGA |
| *ChRDR3-probe-R* | GCAGCCGTGGAGCATCGAAT |
| *hph-F* | ccaagcttCCCTCACTAAAGGGAACAAAAGCT |
| *hph-R* | ggcctaggaGGGGGATCCACTAGTTCTAGA |
| *Phle-F* | ccaagcttgagctcGATCTTTCGACACTGAAATACGT |
| *Phle-R* | ggactagtGGATCTCAACAGCGGTAAGA |
|  | |
| **Primers used for semiquantitative RT-PCR analysis:** | |
| *ChAGO1-ex-F* | GTGGTGGTTACAGAGGCGAT |
| *ChAGO1-ex-R* | GAGGGAGTGTTTTGACCGTAGA |
| *ChAGO2-ex-F* | CCGTGTCAAAGAGTGGAACGACAA |
| *ChAGO2-ex-R* | GATGGGAACCGAGGACCCAAG |
| *ChDCL1-ex-F* | CTGCCGAGCCCACCAACTATGA |
| *ChDCL1-ex-R* | GTTCTGGCCAGTCTGGGTTCAGT |
| *ChDCL2-ex-F* | GTGGAGAGACACTCAGCCTGT |
| *ChDCL2-ex-R* | CCGTGTCGATTGGGAGAGTT |
| *ChRDR1-ex-F* | GTTCGGTACATCCAGAACGACT |
| *ChRDR1-ex-R* | GCCAGCTTCAGGCATCTGT |
| *ChRDR2-ex-F* | CAGTTCGTCGGGCTCATGCA |
| *ChRDR2-ex-R* | GACAGTGTCCTCGTAGACAT |
| *ChRDR3-ex-F* | CCCGCATACAGCTCGGC |
| *ChRDR3--ex-R* | CATGTCAATTGGCGAGCG |
|  | |
| **Primers used for quantitative RT-PCR (qRT-PCR) analysis:** | |
| *ChAGO1-qRT-F* | GAGCTGCCCATGATCCGTAA |
| *ChAGO1-qRT-R* | AATCGAGTCTGGTGACGCTTG |
| *ChAGO2-qRT-F* | AACGCCAAAGGATGAGCAAT |
| *ChAGO2-qRT-R* | ATATCCGCCCCCAGTATCG |
| *ChDCL1-qRT-F* | TCTGCTCAATGCCTGGATCA |
| *ChDCL1-qRT-R* | CCTGAATAGCTCCAGCCGAAT |
| *ChDCL2-qRT-F* | GCCTCGGACATGAACGACTT |
| *ChDCL2-qRT-R* | ATCTGGTCGATGGCAGGTG |
| *ChRDR1-qRT-F* | GATGCCATGGACCGACTGAT |
| *ChRDR1-qRT-R* | CAGCGGGAAAAGCTACCGT |
| *ChRDR2-qRT-F* | GGCTTTGTTGGCGTGAGTTC |
| *ChRDR2-qRT-R* | AAACTGTCCGACATCGACGTC |
| *ChRDR3-qRT-F* | GCGCGAGCATGTTGTTTAAG |
| *ChRDR3-qRT-R* | CGCCATGGTCATCGACTTG |
| *ChTUB-qRT-F* | GGGTCTTGAGCGCCCTAACTA |
| *ChTUB-qRT-R* | GTGATGGAGGAAACGACCTGA |
| *ChActin-qRT-F* | CCCCAAGTCCAACAGAGAGA |
| *ChActin-qRT-R* | CATCAGGTAGTCGGTCAAGTCA |
|  | |
| **Primers used for viral amplification:** | |
| *ORF2-forward* | CGTCTGTAACGTTCTTGGTCTCGTA |
| *ORF1-reverse* | CTCGCGGGTGTGGCAGGCCC |
| *virus-F* | GGCGTTCGTTGCCTTGGGTTT |
| *virus-R* | CCCCTGGTTGCGTTCTGGGA |
| *virus-qRTPCR-1059-F* | TCGACTGGATTACCACCCATC |
| *virus-qRTPCR-1059-R* | CTCCTTGCTTTTAAACGCCG |
|  | |
| **Primers used RNAseq libraries:** | |
| PE Primer-F | AATGATACGGCGACCACCGAGATCTACACTCTTTCCCTACACGACGCTCTTCCGATCT |
| PE-Primer-R-N701 | CAAGCAGAAGACGGCATACGAGATTCGCCTTAGTGACTGGAGTTCAGACGTGT |
| PE-Primer-R-N702 | CAAGCAGAAGACGGCATACGAGATCTAGTACGGTGACTGGAGTTCAGACGTGT |
| PE-Primer-R-N703 | CAAGCAGAAGACGGCATACGAGATTTCTGCCTGTGACTGGAGTTCAGACGTGT |
| PE-Primer-R-N704 | CAAGCAGAAGACGGCATACGAGATGCTCAGGAGTGACTGGAGTTCAGACGTGT |
| PE-Primer-R-N705 | CAAGCAGAAGACGGCATACGAGATGGACTCCTGTGACTGGAGTTCAGACGTGT |
| PE-Primer-R-N706 | CAAGCAGAAGACGGCATACGAGATTAGGCATGGTGACTGGAGTTCAGACGTGT |
| PE-Primer-R-N707 | CAAGCAGAAGACGGCATACGAGATCTCTCTACGTGACTGGAGTTCAGACGTGT |
| PE-Primer-R-N708 | CAAGCAGAAGACGGCATACGAGATCAGAGAGGGTGACTGGAGTTCAGACGTGT |
| PE-Primer-R-N709 | CAAGCAGAAGACGGCATACGAGATGCTACGCTGTGACTGGAGTTCAGACGTGT |
| PE-Primer-R-N710 | CAAGCAGAAGACGGCATACGAGATCGAGGCTGGTGACTGGAGTTCAGACGTGT |
| Adaptor 1 | ACACTCTTTCCCTACACGACGCTCTTCCGATC*T |
| Adaptor 2 | /5Phos/G*ATCGGAAGAGCGGTTCAGCAGGAATGCCGAG |
|  |  |
| **Oligo used for Northern Blot analysis:** | |
| ChNRV1_21nt_smRNA1 | /5Phos/rUrArArGrGrArCrGrUrUrGrGrArCrGrArGrGrUrUrC [‘r’ indicates a ribonucleic acid] |
| *Virus-CP-probe-F* | GTTATGTCCGCACCGAAGCC |
| *Virus-CP-probe-R* | CTAAAAATCCAGAGTCGCCAGGA |
